# Synthesis of triple-decker sandwich compounds featuring a M–M bond through cyclo-Bi_5_ and cyclo-Sb_5_ rings

**DOI:** 10.1038/s41557-025-01765-4

**Published:** 2025-03-18

**Authors:** Yu-He Xu, Xing Yang, Ya-Nan Yang, Lili Zhao, Gernot Frenking, Zhong-Ming Sun

**Affiliations:** 1 State Key Laboratory of Elemento-Organic Chemistry, Tianjin Key Lab of Rare Earth Materials and Applications, School of Material Science and Engineering, Nankai University, Tianjin, China; 2 State Key Laboratory of Materials-Oriented Chemical Engineering, School of Chemistry and Molecular Engineering, Nanjing Tech University, Nanjing, China; 3 College of Chemistry and Molecular Sciences, Henan University, Kaifeng, China; 4 Fachbereich Chemie, Philipps-Universität Marburg, Marburg, Germany; 5 Donostia International Physics Center (DIPC), Donostia, Spain

**Keywords:** Chemical bonding, Synthetic chemistry methodology

## Abstract

The cyclopentadienyl anion is a *π*-aromatic five-membered ring ligand that is widely used in organometallic chemistry. By replacing the CH groups in cyclopentadiene with isoelectronic group-15 elements, an inorganic analogue can be obtained. In this line, Pn_5_ (Pn = P, Sb) rings have been stabilized in a triple-decker sandwich structure, prepared via high-temperature reactions, and an example of a Bi_5_^−^ ring stabilized in a cobalt-based inverse-sandwich-type complex has been reported. Here we report the synthesis and structural characterization of two complexes, [Cp–V(cyclo-Sb_5_)V–Cp]^2−^ and [Cp–Nb(cyclo-Bi_5_)Nb–Cp]^2−^, which are stabilized by [K(18-crown-6)]^+^ or [K(2.2.2-crypt)]^+^ cations at room temperature under mild conditions. Our bonding analysis through various quantum-chemical methods reveals that V‒V and Nb‒Nb bonds pass through the centre of the E_5_ rings (E = Sb, Bi). In contrast to free cyclo-E_5_ (E = Sb, Bi) the cyclo-E_5_ moieties between Cp–E units do not possess any aromatic character because the M‒M (M = V, Nb) bond passes through the centre of the ring.

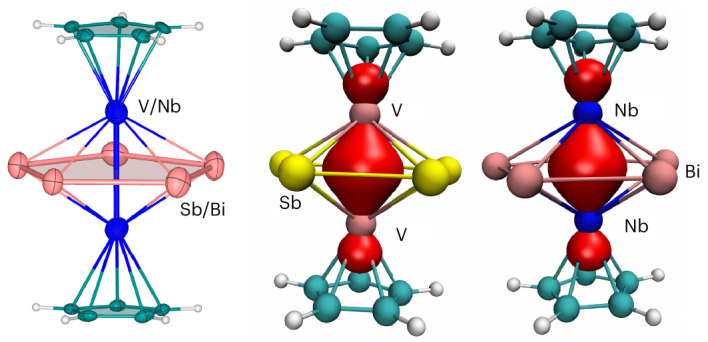

## Abstract (web summary)

Although several inorganic analogues of cyclopentadiene have been reported with group-15 elements, examples featuring the heavier elements Sb and Bi remain scarce. Now, a Bi_5_ ring has been stabilized between two Nb(cyclopentadienyl) units, forming a triple-decker sandwich structure; the bonding analysis indicates that a Nb–Nb bond goes through the centre of the Bi_5_ plane.

## Main

**Fig. 1:**
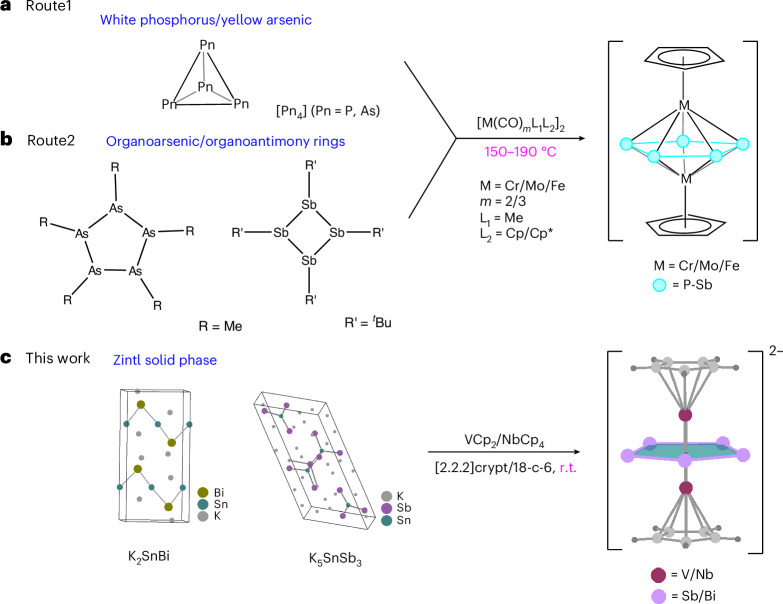
Synthesis routes of the reported triple-decker sandwich compounds and this work. **a**, Synthesis route starting from white phosphorus and yellow arsenic. **b**, Synthesis route starting from organoarsenic and organoantimony. **c**, Synthesis route starting from the Zintl solid phase (this work).

Replacing the CH groups in cyclopentadiene with isoelectronic P atoms results in the phosphorus analogue P_5_, demonstrating an impressive example of the diagonal relationship between carbon and phosphorus^1,2^. The As_5_ ring can subsequently be isolated^3^, and this type of cyclopentadiene-isovalent ligand is typically stabilized in a triple-decker sandwich structure^4,5^. Research on the heavier congeners, Sb and Bi, is limited^6^. One reason for this is that most compounds of this type are prepared using yellow arsenic or white phosphorus as starting materials, which lack suitable sources of antimony and bismuth (Fig. 1a)^7,8^. In addition, organophosphorus and organoarsenic rings can also be applied to form corresponding triple-decker sandwich clusters^3^. A central interlayer cyclo-(*η*^5^-Sb_5_) ligand has been synthesized from the starting material of the organoantimony ring cyclo-^*t*^Bu_4_Sb_4_ (Fig. 1b)^9^. Examples featuring a Bi_5_ ring identified in the solid phase remain elusive, with one example being a [Bi_5_]^−^ pentagon cluster detected by photoelectron spectroscopy (PES)^3,10^, and another being the recently reported Bi_5_^−^ ring stabilized within a mixed-valence inverse-sandwich-type complex (see ‘Quantum-chemical results and bonding analysis’). On the basis of the results of PES and theoretical calculations, the ground state of all the [Pn_5_]^−^ (Pn = P–Bi) species was found to be the aromatic cyclic *D*_5h_ structure. Molecular orbital (MO) analyses revealed that the occupied orbitals in the [Pn_5_]^−^ anions closely resemble those of the isoelectronic [C_5_H_5_]^−^ with the same set of *π* orbitals. The coordinating transition metals of the reported [Pn_5_]^−^ complex consisted of Cr, Mo and Fe, with the corresponding organometallic precursors containing two or three carbonyl groups along with cyclic organic ligands. The compounds of this kind reported thus far are synthesized at high temperatures (at least 150 °C or above), which may be due to the presence of carbonyl groups on the metals, which require high temperatures for their decoordination.

In this Article we present a rare example of a Bi_5_ ring, stabilized by two NbCp groups in the triple-decker sandwich compound [Cp–Nb(cyclo-Bi_5_)Nb–Cp]^2−^. Its lighter homologue, cyclo-Sb_5_, is also stabilized in the isostructural cluster [Cp–V(cyclo-Sb_5_)V–Cp]^2−^. These two clusters can be synthesized under mild conditions at room temperature (Fig. 1c). Consequently, this one-pot approach offers substantial advantages over previous synthetic methods. The core structure of both clusters is pentagonal bipyramidal, with unique V–V and Nb–Nb bonding significantly affecting the electronic structure of the clusters, which distinguishes them from previously reported neutral triple-decker sandwich compounds. Note that there have been previous reports on the stabilization of the five-membered ring of group-14 heavy metals by two metal carbonyl ligands ([Pb_5_{Mo(CO)_3_}_2_]^4−^ and [Sn_5_{Cr(CO)_3_}_2_]^4−^)^11,12^. In these examples, there is no direct bonding between the transition metals, so the V–V and Nb–Nb bonding through the central cyclo-E_5_ unit in this work is a rare case among such pentagonal bipyramidal all-metal clusters. Theoretical calculations further confirmed the existence of one M‒M bond (M = V, Nb) and 10 M‒E bonds (M = V, Nb; E = Sb, Bi). Further analysis of the nucleus-independent chemical shift (NICS) values indicated that the two five-membered rings no longer possess any aromaticity, as is observed in the free Sb_5_^−^ and Bi_5_^−^ rings. While the present work was being carried out, a study by Weigend, Dehnen and co-workers about a related compound reported the synthesis of a mixed-valence complex [IMes–Co(cyclo-Bi_5_)Co–IMes], where the IMes ligand is an N-heterocyclic carbene with bulky substituents (bis(1,3-(2,4,6-trimethylphenyl)imidazole-2-ylidene)^13^. The neutral compound has an electronic doublet ground state. The following sections include a discussion of both compounds from a theoretical perspective.

## Results and discussion

### Experimental results

The compound [K(18-crown-6)]_2.5_[Cp–V(cyclo-Sb_5_)V–Cp]·0.5Cp·3.5Py (**1**) was synthesized by the reaction of K_5_SnSb_3_ and VCp_2_ in the presence of 18-crown-6. After stirring at room temperature (r.t.) for 3 h, the ethylenediamine (en) solvent was dried under vacuum, and the resulting brown solid was dissolved in pyridine (Py). After an additional hour of stirring, the reaction mixture was filtered, layered with 3 ml of toluene, and allowed to stand for one week to obtain black plate-shaped crystals. Single-crystal X-ray diffraction (XRD) determination and refinement revealed that **1** crystallizes in the monoclinic space group *C*_2/*m*_, with the additional inclusion of two [K(18-crown-6)]^+^ cations, three Py solvate molecules and half [K(18-crown-6)]Cp molecules in the asymmetric unit. Reacting the Zintl-phase K_2_SnBi with the organometallic precursor NbCp_4_ in a mixture of en and [2.2.2]crypt solutions for 6 h at r.t. led to the formation of the complex [K(2.2.2-crypt)]_2_[Cp–Nb(cyclo-Bi_5_)Nb–Cp]·0.5en·1.5tol (**2**). After being layered with 4 ml of toluene, black block crystals were obtained after storage for one week. Compound **2** crystallizes in the triclinic space group *P*–1 and comprises one anionic cluster, two [K(2,2,2-crypt)]^+^ charge-balancing cations, one toluene molecule and half an en solvate molecule in each asymmetric unit.

**Fig. 2:**
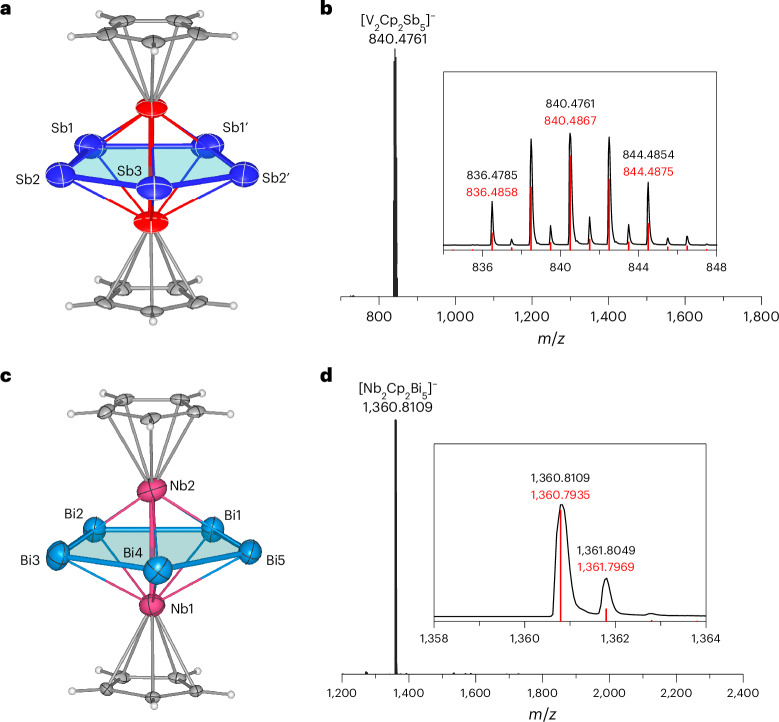
Molecular structure and MS analysis of [Cp–V(cyclo-Sb_5_) V–Cp]^2−^ and [Cp–Nb(cyclo-Bi_5_) Nb–Cp]^2−^. **a**, X-ray structure of the [Cp–V(cyclo-Sb_5_) V–Cp]^2−^ anion drawn with thermal ellipsoids at the 50% level. **b**, Negative-ion-mode ESI mass peak corresponding to [Cp–V(cyclo-Sb_5_) V–Cp]^−^. **c**, X-ray structure of the [Cp–Nb(cyclo-Bi_5_) Nb–Cp]^2−^ anion drawn with thermal ellipsoids at the 50% level. **d**, Negative-ion-mode ESI mass peak corresponding to [Cp–Nb(cyclo-Bi_5_) Nb–Cp]^−^. Experimental mass distributions are depicted in black, and theoretical masses of the isotope distribution are in red.

As shown in Fig. 2a, the main structure of cluster **1** is a pentagonal bipyramidal configuration in which two V atoms cap the Sb_5_ ring. With the two Cp ligands connected to the two V atoms, the molecule can also be viewed as a typical triple-decker sandwich. The Sb‒Sb bond lengths range from 2.7776(7) to 2.8671(10) Å, with two relatively shorter bonds of 2.7776(7) Å and three longer Sb‒Sb single bonds ranging from 2.8237(8) to 2.8671(10) Å). When compared with the Sb planar pentagon found in the neutral cluster [(*η*^5^-1,2,4-^*t*^Bu_3_C_5_H_2_)_2_Mo_2_(*μ*,*η*^5^-Sb_5_)]^9^, the distribution of Sb‒Sb bond lengths on the Sb_5_ rings in the two clusters is similar, there also being two shorter Sb‒Sb bonds (2.7559(11) Å and 2.7656(9) Å) and three typical Sb‒Sb single bonds of 2.7978(8)–2.8504(9) Å. All V‒Sb bonds span a range of 2.7659(16)–2.8453(16) Å. There have been no previous reports on V‒Sb bonds as reported in this work. The V–V bond is 2.9028(24) Å in length, which is substantially shorter than the nonbonding V–V contact (3.403(1) Å) in a similar triple-decker sandwich with a benzene ring (CpV)_2_[*μ*-(*η*^6^-C_6_H_6_)], comparable to the V–V bond (2.978(3) Å) in [V_2_(*μ*-Cl)_3_(THF)_6_] BPh_4_, and longer than that of vanadaborane [(CpV)_2_(B_2_H_6_)_2_] (2.787(2) Å)^14–16^. Cluster **2** is isostructural with cluster **1**, with different metal elements making up the main structural framework, as shown in Fig. 2c. The Bi_5_ ring was first identified in the solid phase and has one relatively short Bi–Bi bond (2.9201(7) Å) that is comparable to the Bi–Bi^−^ (2.9164(14)–2.9489(17) Å) bonds observed in [Bi_11_]^3−^, whereas the others exist in a narrow bond distance range of 3.0040(5)–3.1358(7) Å (ref. ^17^). All Nb–Bi bonds are between 2.9030(8) and 3.0262(8) Å long, comparable to those in the anionic cluster [Ga@Bi_10_(NbMes)_2_]^3−^ (2.9421(10)–3.0104(11) Å)^18^. The Nb–Nb contact is 2.9542(4) Å, close to that of [(CpNb)_2_(B_2_H_6_)_2_] (2.9477(16) Å)^16^. Electrospray ionization mass spectrometry (ESI–MS) spectra of clusters **1** and **2** in dimethylformamide (DMF) solutions show peaks at *m*/*z* = 840.4761 and 1,360.8109 that are identified as [Cp–V(cyclo-Sb_5_) V–Cp]^−^ and [Cp–Nb(cyclo-Bi_5_) Nb–Cp]^−^, respectively (Fig. 2b,d). No additional fragment peaks were detected, suggesting that under MS conditions, compounds **1** and **2** are relatively stable in DMF solution, even though their overall charge is substantially lower than that of the anions found in the crystal.

### Quantum-chemical results and bonding analysis

DFT calculations were performed at the BP86-D3(BJ)/def2-TZVPP level to elucidate the electronic structure and bonding nature of compounds [V_2_Cp_2_Sb_5_]^2−^ and [Nb_2_Cp_2_Bi_5_]^2−^. Supplementary Fig. 12 shows the calculated geometries and the most important bond lengths and angles, which agree very well with the experimental values. Both [V_2_Cp_2_Sb_5_]^2–^ and [Nb_2_Cp_2_Bi_5_]^2–^ exhibit doublet (^2^B_1_) ground states with *C*_2*V*_ symmetry, which are 17.5 and 21.0 kcal mol^−1^ lower in energy than the respective quartet (^4^A_1_) states. The spin density distributions of the two compounds indicate that the unpaired electron is localized mainly at the V and Nb centres (Supplementary Fig. 12).

A pivotal question concerns the electronic structure and bonding situation of the M(cyclo-E_5_)M (M = V, Nb; E = Sb, Bi) core moieties in the two dianions. The [M_2_Cp_2_E_5_]^2−^ molecules may be considered triple-decker sandwich complexes where a cyclic E_5_^−^ ring, which is valence-isoelectronic to Cp^−^, is *η*^5^-bonded to the metal atoms above and below the ring. This is a formally correct view, but the results of the bonding analysis using several methods suggest that an alternative view of the M(cyclo-E_5_)M core moiety is more reasonable. Extended Data Table 1 presents the results of the NICS calculations for free E_5_^−^ and Cp^−^ via the optimized geometry and frozen geometry of the complex. The data for the ring currents are typical for aromatic systems and support the notion that cyclic Sb_5_^−^ and Bi_5_^−^ are aromatic systems that are valence-isoelectronic to Cp_5_^−^. Extended Data Table 1 also lists the NICS values of E_5_^−^ and Cp^−^ in the complexes. The Cp^−^ ring retains its negative value of diamagnetic current in the ring (NICS(0)) and outward region (NICS(1)_zz_ and NICS(−1)_zz_) of the complexes. In contrast, the cyclic Sb_5_^−^ and Bi_5_^−^ moieties not only have large positive NICS(1)_zz_ and NICS(−1)_zz_ values, but the NICS(0) value at the centre of the rings even becomes slightly positive, which indicates the absence of aromatic character.

**Fig. 3:**
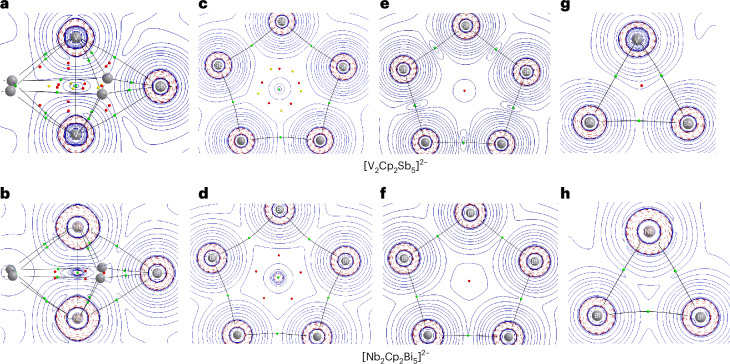
Laplacian distributions of [V_2_Cp_2_Sb_5_]^2−^ and [Nb_2_Cp_2_Bi_5_]^2−^ at the BP86-D3(BJ)/def2-TZVPP level in different planes. **a**, The V–V–Sb plane, showing all critical points in V(cyclo-Sb_5_)V. **b**, The Nb–Nb–Bi plane, showing all critical points in Nb(cyclo-Bi_5_)Nb. **c**, The Sb_5_ plane in [V_2_Cp_2_Sb_5_]2−. **d**,The Bi_5_ plane in [Nb_2_Cp_2_Bi_5_]^2−^. **e**, The free Sb_5_^−^ plane in frozen geometry. **f**, The free Bi_5_^−^ plane in frozen geometry. **g**, The V–Sb–Sb plane. **h**, The Nb–Bi–Bi plane. Red lines indicate areas of charge concentration (∇^2^*ρ*(*r*) < 0), and blue lines represent areas of charge depletion (∇^2^*ρ*(*r*) > 0). Solid lines connecting the atomic nuclei are bond paths. Green dots are bond critical points, red dots are ring critical points, and yellow dots are cage critical points.

Useful information about the electronic structure of the molecules was obtained by analysis using the quantum theory of atoms in molecules (QTAIM). Figure 3 shows the Laplacian distribution ∇^2^*ρ*(**r**) of the electronic charge, with selected moieties of the complexes displayed. Figure 3a,b shows the M(cyclo-E_5_)M core moieties of [V_2_Cp_2_Sb_5_]^2−^ and [Nb_2_Cp_2_Bi_5_]^2−^, and Fig. 3c,d presents the E_5_ rings in the complexes. For comparison, Fig. 3e,f shows the Laplacian distribution ∇^2^*ρ*(**r**) of the naked E^−^ moieties. Figure 3g,h displays the E_2_M planes of the complexes.

Crucial information comes from the M(cyclo-E_5_)M core moieties. Figure 3a,b shows that there are bond critical points (bcps, green dots) for all ten M–E bonds and five E‒E bonds. However, there is also a bcp for the V–V and Nb–Nb interactions instead of a ring critical point (rcp) in the centre of the E_5_ ring. The area of charge concentration at the midpoint of the V‒V and Nb‒Nb axes suggests the appearance of V‒V and Nb‒Nb bonds. This is supported by the numerical values at the M–M bcps, which are shown in Supplementary Table 2. In particular, the energy value at bcp H_**r**_, which has been shown to be a very sensitive indicator of the nature of a bond^19^, suggests that the M–M bonds have even greater covalent character than the E‒E bonds. Notably, the calculated Nb‒Nb distance in [Nb_2_Cp_2_Bi_5_]^2−^ is 2.949 Å, which agrees with the standard Nb‒Nb single bond value of 2.94 Å, and the computed V‒V distance in [V_2_Cp_2_Sb_5_]^2−^ of 2.822 Å is only slightly longer than the average value of 2.68 Å for a single bond^16^. This introduces a different new perspective for complexes [V_2_Cp_2_Sb_5_]^2−^ and [Nb_2_Cp_2_Bi_5_]^2−^, where the neutral core moieties M(cyclo-E_5_)M are cage units with central M‒M bonds that are capped by Cp^−^ ligands.

The QTAIM results revealed subtle differences between the two complexes. Figure 3a,b shows that there are 15 rcps and five cage critical points (ccps, yellow dots) in V(cyclo-Sb_5_)V, but only five rcps and no ccp in Nb(cyclo-Bi_5_)Nb. Topological analysis of the electron density yields five M_2_E_2_ segments resembling ‘melon chunks’ with associated rcps in V(cyclo-Sb_5_)V, which are not found in Nb(cyclo-Bi_5_)Nb. Accordingly, the Laplacian distribution in the E_5_ rings (Fig. 3c,d) is five rcps and five ccps in addition to the five Sb‒Sb bcps and the single V‒V bcp in the Sb_5_ cycle, but there are only five rcps and five Bi‒Bi bcps in the Nb‒Nb bcp in the Bi_5_ ring.

The appearance of the M–M bond in the complexes is clearly visible by comparing the Laplacian distribution of the E_5_ rings in the complexes (Fig. 3c,d) with that of free E_5_^−^ rings with the same geometry (Fig. 3e,f). The naked E_5_^−^ rings possess a single rcp in the centre of the ring, but there is a bcp in the centre of the E_5_ fragments of the complexes, which is surrounded by an area of relative charge concentration. Figure 3a,b reveals that the charge concentration comes from the formation of the M‒M bond. There is a hole at the centre of the naked E_5_^−^ rings that is filled by the M‒M bond. The hole has been reported previously. An earlier study by one group showed that the [Fe(cyclo-E_5_)]^+^ cations have a pyramidal geometry (*C*_5*v*_) when E = N, P, As, but heavier homologues with E = Sb, Bi have a planar *D*_5*h*_ geometry where the Fe atom fills the hole in the ring^20^. In complexes [Nb_2_Cp_2_Bi_5_]^2−^ and [V_2_Cp_2_Sb_5_]^2−^, the M‒M bond fills the hole in the E_5_ ring.

We calculated the charge distributions of [V_2_Cp_2_Sb_5_]^2−^ and [Nb_2_Cp_2_Bi_5_]^2–^ with the NBO method and the Wiberg bond order. Extended Data Table 2 shows that most of the negative charge in the dianions rests on the metal atoms V_2_ (−1.20*e*) or Nb_2_ (−1.18*e*) and the Cp_2_ rings (−1.00*e* in the V complex and −0.68*e* in the Nb species). In contrast, the Bi_5_ ring has only a small negative charge (−0.14*e*), and the Sb_5_ ring even has a positive partial charge (0.20*e*). More interesting are the bond orders. Extended Data Table 2 also shows the bond orders for the V–V (0.63) and Nb–Nb bonds (0.68), which have a magnitude similar to those of the Sb–Sb bonds (0.52–0.75) and Bi–Bi bonds (0.50–0.73) of the E_5_ rings. Note that the calculations give much smaller bond-order values for the C–V and C–Nb bonds (0.26).

**Fig. 4:**
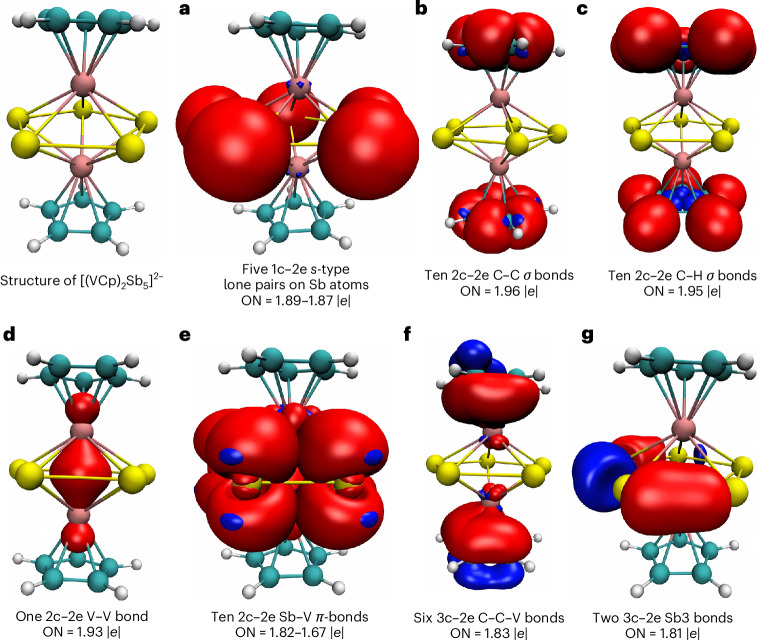
Results of the AdNDP calculations for [V_2_Cp_2_Sb_5_]^2−^. ON denotes occupation numbers. Sb atoms are shown in dark yellow, V atoms in pink, Catoms in cyan and H atoms in white.

To find the best description of the chemical bonds in the [M_2_Cp_2_E_5_]^2−^ complexes we used the adaptive natural density partitioning (AdNDP) method^21–23^, which was developed by Boldyrev as an extension of the NBO approach, to include multicentre bonds in addition to the classic 2-centre–2-electron bonds (2c‒2e) and 1-centre–2-electron moieties (1c‒2e, lone pair). Figure 4 shows the most important results. In [V_2_Cp_2_Sb_5_]^2−^ there are only two 3c‒2e bonds with occupation numbers (ON) of 1.81*e* for the Sb_5_ ring (Fig. 4g), but there are ten 2c‒2e V–Sb bonds (ON = 1.67–1.82; Fig. 4e) along with five 1c‒2e lone pairs (ON 1.87–1.89; Fig. 4a). However, there is also a V‒V bond with a high ON angle of 1.93 (Fig. 4d). Furthermore, six 3c–2e bonds exist for the Cp‒V moiety (ON = 1.83; Fig. 4f), where the contribution of V appears very small. The six bonds can be assigned to the aromatic *π* bonds of the Cp ring, which are polarized towards V. There are also ten 2c‒2e bonds for the C‒C *σ* bonds (ON = 1.96; Fig. 4b) and ten 2c‒2e bonds for the C‒H bonds (ON = 1.95; Fig. 4c). The bond orbital found with AdNDP for the V‒V bond is also found in the canonical MOs of the molecule. Supplementary Fig. 15 shows the singly occupied SOMO-1 of the α and β electrons of [V_2_Cp_2_Sb_5_]^2−^, which are a bonding combination of the *d*_*z*_2 atomic orbitals (AOs) of the V atom (Supplementary Fig. 15).

The AdNDP results for [Nb_2_Cp_2_Bi_5_]^2−^ are very similar to those for [V_2_Cp_2_Sb_5_]^2−^, as shown in Supplementary Fig. 16. The bonding patterns suggested by the AdNDP results for both dianions agree well with the findings of the QTAIM analysis, the calculated charge distribution and bond orders, as well as the NICS calculations. The complexes should not be considered triple-decker sandwich complexes containing a Sb_5_^−^ or Bi_5_^−^ moiety. The combined results of the bonding analysis suggest that [Nb_2_Cp_2_Bi_5_]^2−^ and [V_2_Cp_2_Sb_5_]^2−^ have an M(cyclo-E_5_)M core moiety that has one M‒M bond and ten M‒E bonds.

The finding of a M–M bond in the M(cyclo-E_5_)M core moiety through the centre of the E_5_ ring was surprising. We wanted to know whether it actually was a 2c–2e bond, as suggested by the AdNDP method, or whether it was rather a multicentre bond in which the E_5_ ring was involved to some extent in bond formation. To this end, we carried out energy decomposition analysis coupled with natural orbitals for chemical valence (EDA-NOCV) calculations of [V_2_Cp_2_Sb_5_]^2−^ and [Nb_2_Cp_2_Bi_5_]^2−^ using cyclo-Sb_5_/Bi_5_ and (VCp)_2_/(NbCp)_2_ with various charges and electronic states as the interacting fragments. The best fragments were [cyclo-E_5_]^−^ in the electronic singlet state and [(VCp)_2_]^−^/[(NbCp)_2_]^−^ in the doublet (^2^B_1_) state, because they change the least during bond formation, as indicated by their smallest Δ*E*_orb_ values^24^. Table 1 shows the numerical results of the EDA-NOCV calculations with these fragments. The calculations using other charges and electronic states are shown in Supplementary Tables 3 and 4.

**Table 1:** EDA-NOCV results of [V_2_Cp_2_Sb_5_]^2−^ and [Nb_2_Cp_2_Bi_5_]^2−^ at the BP86-D3(BJ)/TZ2P level of theory

	[V_2_Cp_2_Sb_5_]^2−^		[Nb_2_Cp_2_Bi_5_]^2−^	
	Orbital interaction	Fragments [cyclo-Sb_5_]^−^ (S) + [(VCp)_2_]^−^ (D)	Orbital interaction	Fragments [cyclo-Bi_5_]^−^ (S) + [(NbCp)_2_]^−^ (D)
Δ*E*_int_		−308.6		−283.4
Δ*E*_Pauli_		682.7		1,020.1
Δ*E*_elstat_^a^		−503.8 (50.8%)		−775.9 (59.5%)
Δ*E*_disp_^a^		−48.9 (4.9%)		−50.0 (3.8%)
Δ*E*_orb_^a^		−438.6 (44.3%)		−477.6 (36.7%)
Δ*E*_orb(1)_^b^	Sb_5_^1−^ ← (VCp)_2_^1−^ donation	−89.3 (20.4%)	Nb–Nb bond	−90.2 (18.9%)
Δ*E*_orb(2)_^b^	Sb_5_^1−^ ← (VCp)_2_^1−^ donation	−85.8 (19.6%)	Bi_5_^1−^ ← (NbCp)_2_^1−^ donation	−88.0 (18.4%)
Δ*E*_orb(3)_^b^	Sb_5_^1−^ ← (VCp)_2_^1−^ donation	−80.7 (18.4%)	Bi_5_^1−^ ← (NbCp)_2_^1−^ donation	−74.8 (15.7%)
Δ*E*_orb(4)_^b^	Sb–Sb bond	−65.8 (15.0%)	Bi_5_^1−^ ← (NbCp)_2_^1−^ donation	−70.2 (14.7%)
Δ*E*_orb(5)_^b^	Sb_5_^1−^ ← (VCp)_2_^1−^ donation	−31.8 (7.3%)	Bi_5_^1−^ ← (NbCp)_2_^1−^ donation	−59.9 (12.5%)
Δ*E*_orb(6)_^b^	Sb_5_^1−^ → (VCp)_2_^1−^ donation	−21.7 (4.9%)	Bi_5_^1−^ → (NbCp)_2_^1−^ donation	−22.5 (4.7%)
Δ*E*_orb(7)_^b^	Sb_5_^1−^ → (VCp)_2_^1−^ donation	−21.4 (4.9%)	Bi_5_^1−^ → (NbCp)_2_^1−^ donation	−19.1 (4.0%)
Δ*E*_orb(8)_^b^	Sb_5_^1−^ → (VCp)_2_^1−^ donation	−12.5 (2.8%)	Bi_5_^1−^ → (NbCp)_2_^1−^ donation	−18.4 (3.9%)
Δ*E*_orb(9)_^b^	Sb_5_^1−^ → (VCp)_2_^1−^ donation	−6.2 (1.4%)	Bi_5_^1−^ → (NbCp)_2_^1−^ donation	−6.6 (1.4%)
Δ*E*_orb(10)_^b^	Sb_5_^1−^ → (VCp)_2_^1−^ donation	−6.2 (1.4%)	Bi_5_^1−^ → (NbCp)_2_^1−^ donation	−6.3 (1.3%)
Δ*E*_orb(11)_^b^	Sb_5_^1−^ → (VCp)_2_^1−^ donation	−2.9 (0.7%)	Bi_5_^1−^ → (NbCp)_2_^1−^ donation	−5.0 (1.0%)
Δ*E*_orb(rest)_^b^		−14.3 (3.3%)		−16.6 (3.5%)

^a^Values in parentheses give the percentage contribution to the total attractive interactions Δ*E*_elstat_ + Δ*E*_orb_ + Δ*E*_disp_.

^b^Values in parentheses give the percentage contribution to the total orbital interactions Δ*E*_orb_.

Fragments are given on the table in singlet (S) or doublet (D) electronic states. Energy values are given in kcal mol^−1^.

**Fig. 5:**
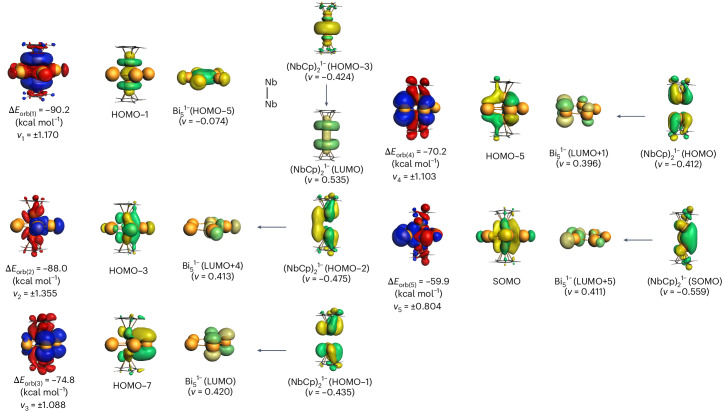
Plot of the associated deformation densities Δ*ρ* of the pairwise orbital interactions of the α and β electrons of Δ*E*_orb(1)_–Δ*E*_orb(5)_ and the most important orbitals in [Nb_2_Cp_2_Bi_5_]^2−^. The direction of charge flow is from red to blue. The deformation density Δ*ρ* represents the flow of the total electronic charge (α + β electrons) in the red→blue direction. The orbital figures show only the orbitals that are occupied by α electrons. The figures for the β electrons are very similar and are provided in the Supplementary Information.

The numerical results in Table 1 suggest that the covalent (orbital) interactions contribute 44% ([V_2_Cp_2_Sb_5_]^2−^) and 37% ([Nb_2_Cp_2_Bi_5_]^2−^) to the total interactions, Δ*E*_int_. The most important information pertinent for our question comes from the breakdown of the Δ*E*_orb_ term into pairwise orbital contributions, which deserve particular attention. Table 1 lists 11 pairwise orbital interactions Δ*E*_orb(1)_–Δ*E*_orb(11)_ that contribute to the bonding interactions between the fragments. The remaining 3%, termed Δ*E*_orb(rest)_, comes from intrafragment relaxation. An examination of the associated deformation densities and the connected orbitals (Fig. 5) revealed that ten orbital interactions arise from the formation of the M–E_5_ bonds of the cage, and one orbital interaction can clearly be associated with the formation of the M–M bond with a negligible contribution from the E_5_^−^ ring. This is Δ*E*_orb(4)_ for [V_2_Cp_2_Sb_5_]^2−^ and Δ*E*_orb(1)_ for [Nb_2_Cp_2_Bi_5_]^2−^, which is the strongest orbital contribution for the molecule. The shapes of HOMO-3 (HOMO, highest occupied molecular orbital) and the lowest unoccupied molecular orbital (LUMO) of the (NbCp)_2_^1−^ fragments and HOMO-1 of [Nb_2_Cp_2_Bi_5_]^2−^ reveal that Nb–Nb bond formation is associated with substantial hybridization at Nb, where the large 5*s* contribution decreases and the bond comes mainly from the *d*_z_2 AOs of Nb. Figure 5 shows the deformation density Δ*ρ*, given by the sum of the α and β electrons and the connected orbitals that are associated with Δ*E*_orb(1)_ for [Nb_2_Cp_2_Bi_5_]^2−^. The figure also shows the deformation density Δ*ρ* and the connected orbitals associated with the Δ*E*_orb(2)_–Δ*E*_orb(5)_ of [Nb_2_Cp_2_Bi_5_]^2−^. They can easily be identified via the formation of Nb–Bi_5_ bonds. The deformation density Δ*ρ* and the connected orbitals of the other orbital interactions ΔE_orb(6)_–ΔE_orb(11)_ of [Nb_2_Cp_2_Bi_5_]^2−^, along with those of ΔE_orb(1)_–ΔE_orb(11)_ of [V_2_Cp_2_Sb_5_]^2−^, are shown for the α and β electrons in Supplementary Figs. 17–20.

Figure 5 shows that the formation of the Nb‒Nb bond via the orbital interaction Δ*E*_orb(1)_ of [Nb_2_Cp_2_Bi_5_]^2−^ involves nearly exclusively the HOMO-3 and LUMO of the [(NbCp)_2_]^−^ species. The eigenvalues (*v*) of the associated charge flow indicate that the contribution of the [cyclo-Bi_5_]^−^ fragment is very small (eigenvalue of 0.074). The same holds true for the orbital interaction Δ*E*_orb(4)_ of [V_2_Cp_2_Sb_5_]^2−^ (Supplementary Fig. 18). Accordingly, there are genuine V–V and Nb–Nb bonds passing through the centre of the E_5_ rings.

The complex [IMes–Co(cyclo-Bi_5_)Co–IMes] reported by Weigend, Dehnen and co-workers was introduced as a landmark in the chemistry of all-metal aromatic molecules^13^. Their extended bonding analysis showed that the aromatic character of naked cyclo-Bi_5_^−^ obtained using the GIMIC^25,26^ method is similar to that of Cp^−^, which agrees with our NICS calculations. However, the GIMIC value of the cyclo-Bi_5_ unit in their sandwich complex [IMes–Co(cyclo-Bi5)Co–IMes] was not provided. The authors reported that HOMO–4 is the bonding combination of the Co(*d*) orbitals along the Co‒Co axis and that the Pipek‒Mezey-localized MOs form a Co‒Co bond. These findings suggest that the bonding situation in the Co(cyclo-Bi_5_)Co core is similar to the bonding situation in the V(cyclo-Sb_5_)V and Nb(cyclo-Bi_5_)Nb moieties of our dianions. This would imply that the neutral complex [IMes–Co(cyclo-Bi_5_)Co–IMes] reported by Weigend et al.^13^ and our dianions, [Cp–M(cyclo-E_5_)M–Cp]^2−^, do not possess aromatic E_5_^−^ (E = Sb, Bi) ligands and that they are not all-metal aromatic molecules. Instead, they feature a M(cyclo-E_5_)M species with a central M‒M bond, which, to the best of our knowledge, indeed represents a unique type of molecular structure. It may be possible that such M(cyclo-E_5_)M species with M‒M bonds are only observed for heavier atoms E or with larger rings E_*n*_. As noted above, the cations [Fe(cyclo-E_5_)]^+^ have a pyramidal geometry (*C*_5*v*_) when E = N, P, As, but the heavier homologues with E = Sb, Bi have a planar *D*_5*h*_ geometry^27^.

Molecules with atomic rings penetrated by a chemical bond have been observed previously. Examples include catenanes and molecular machines, where several rings are interlocked^28^. The distinctive new feature of the complexes reported here, [Cp–M(cyclo-E_5_)M–Cp]^2−^, are the bonds between the metal atoms M and the ring atoms E, which establish a cage structure with a central M‒M bond.

Numerous neutral and charged complexes have been reported with the general formula [L–M(cyclo-E_5_)M–L]^*q*^ where E = P, As, Sb and M = Fe, Cr, Ni, Mo, which are stabilized by various ligands L (refs. ^3–7,28–31^). All of these complexes were labelled triple-decker complexes containing an E_5_^−^ moiety. Investigating the homologues [L–M(cyclo-E_5_)M–L]^*q*^ with heavier cyclo-Sb_5_^−^ and cyclo-Bi_5_^−^ groups is useful. It is possible that they possess a core unit M(cyclo-E_5_)M that has not been recognized until now. It appears from this work and from the results of the earlier study of [Fe(cyclo-E_5_)]^+^^27^ that cyclo-Sb_5_^−^ and cyclo-Bi_5_^−^ have an electronic hole at the centre, which may accommodate a single atom or a M–M bond that penetrates the ring. We will continue our research in this direction.

## Conclusion

In summary, we have employed the Zintl solid phase as the source of antimony and bismuth to synthesize two complexes containing planar Sb_5_ and Bi_5_ rings under mild reaction conditions. The core structure of the cluster is a pentagonal bipyramidal structure, with the distinctive feature that the V–V and Nb–Nb bonds cross the central pentagonal plane, providing examples of inverse-sandwich compounds featuring transition-metal atoms at both ends of the Pn_5_ ring. Analysis of the electronic structure with several quantum-chemical methods revealed the existence of V–V and Nb–Nb bonds and showed that the interlayered cyclo-E_5_ (E = Sb, Bi) rings do not possess any aromatic character. This work not only demonstrates an alternative synthetic strategy but also provides a research model for planar clusters of heavier group-15 elements, highlighting the coordination versatility in elemental Sb and Bi. The results reported here and in earlier theoretical studies demonstrate that the variation of the molecular structure of group-15 atoms for the heavier atoms Sb and Bi may cause not only quantitative but also qualitative changes, which lead to hitherto unknown bonding situations.

## Methods

### General methods

All manipulations and reactions were conducted under a nitrogen atmosphere using standard Schlenk or glovebox techniques. Ethylenediamine (en; Aldrich, 99%) and pyridine (Py; Aldrich, 99.8%) were freshly distilled with CaH_2_ before use, then stored in N_2_. Toluene (Aldrich, 99.8%) was distilled from sodium/benzophenone under nitrogen and stored under nitrogen. 2.2.2-Crypt (4,7,13,16,21,24-hexaoxa-1,10-diazabicyclo (8.8.8) hexacosane; Sigma-Aldrich, 98%) and 18-crown-6 (1,4,7,10,13,16-hexaoxacyclooctadecane) were dried in vacuum for one day before use. K_2_SnBi and K_5_SnSb_3_ were prepared by heating a stoichiometric mixture of the corresponding elements in Nb tubes^32,33^. For K_2_SnBi, the Nb tube was sealed by arc-welding, placed in an oven and kept at 850 °C for 36 h. The same steps were applied to synthesize a phase with the nominal composition ‘K_5_SnSb_3_’. VCp_2_ and NbCp_4_ were synthesized according to methods in the literature^34,35^. Details of the synthetic procedures are described in the following sections.

### Synthesis of VCp_2_

VCl_3_ (15.73 g) was dissolved in 150 ml of anhydrous and oxygen-free THF. The mixture was refluxed for 12 h, after which 16.9 g of zinc powder (3 equiv.) was added to the reaction flask. Refluxing was continued overnight, resulting in the formation of a dark-green slurry. NaCp (17.617 g, 2 equiv.) was dissolved in 75 ml of THF and added dropwise to the VCl_3_ solution via a dropping funnel. The mixture was refluxed for 4 h, then allowed to return to r.t. and left to react overnight. The reaction mixture was filtered through a column packed with diatomaceous earth, and the reaction vessel was rinsed several times with THF. The filtrate was collected, and the solvent was removed under vacuum. The residue was extracted with 75 ml of *n*-hexane, and insoluble materials were removed by filtration. The solvent was evaporated under vacuum, yielding 10.74 g (59.29%) of dark-purple solid.

### Synthesis of NbCp_4_

NaCp (22.6 g) was dissolved in 400 ml of toluene to form a greyish-white suspension. Specifically, NbCl_5_ (6.6 g) was dissolved in 100 ml of toluene. Under stirring, the NbCl_5_ solution was added dropwise to the NaCp suspension, during which the colour of the suspension visibly changed from greyish-white to purple and then to brown. The reaction mixture was stirred for an additional 2 h. On completion of the reaction, the mixture was filtered through diatomaceous earth, and the residue was washed with toluene until the filtrate became colourless. The resulting filtrate was evaporated to dryness to obtain a purple-brown solid. This solid was extracted with 100 ml of *n*-heptane in small portions multiple times. Filtration of the extract yielded a dark-purple solid, identified as NbCp_4_. The product was thoroughly dried, resulting in a final weight of 8.7 g, corresponding to a yield of 87%.

### Synthesis of [K(18-crown-6)]_2.5_[V_2_Cp_2_Sb_5_]·0.5Cp·3.5Py (1)

K_5_SnSb_3_ (68 mg, 0.1 mmol) and 18-crown-6 (79 mg, 0.3 mmol) were dissolved in 2.5 ml of each mixture and stirred for 0.5 h to yield a dark-brown solution. VCp_2_ (17 mg, 0.094 mmol) was added to the reaction mixture, resulting in a red-brown suspension, which was stirred for 3 h at r.t. The mixture was dried, 3 ml of pyridine was added, and the mixture was stirred for 3 h. The resulting solution was filtered through glass wool, transferred to a test tube, then carefully layered with toluene (3 ml) to allow for crystallization. After one week, black block crystals of [K(18-crown-6)]_2.5_[V_2_Cp_2_Sb_5_]·0.5Cp·3.5Py (**1**) were obtained in ~19% crystal yield (based on VCp_2_). The collected crystals of (**1**) were characterized by XRD, energy-dispersive X-ray spectroscopy (EDX) and MS. The resulting compound compositions were consistent, maximally ensuring the purity of the crystals obtained from the reaction.

### Synthesis of [K(2.2.2-crypt)]_2_[Nb_2_Cp_2_Bi_5_]·0.5en·1.5tol (2)

K_2_SnBi (60 mg, 0.148 mmol) and 2.2.2-crypt (107 mg, 0.296 mmol) were dissolved in 2.5 ml of en and stirred for 5 min to yield a dark-green solution. NbCp_4_ (30 mg, 0.0489 mmol) was added to the reaction mixture, resulting in a dark-brown suspension, which was stirred for 2 h at 50 °C. The resulting solution was filtered through glass wool, transferred to a test tube, and then carefully layered with toluene (3 ml) to allow for crystallization. After one week, black block crystals of [K(2.2.2-crypt)]_2_[Nb_2_Cp_2_Bi_5_]·0.5en·1.5tol (**2**) were obtained in ~16% crystal yield (based on NbCp_4_). The characterizations for compound **2** were the same as those for the crystals of **1**.

### XRD

Suitable single crystals of compounds **1** and **2** were selected for XRD analyses. Crystallographic data were collected on a Rigaku XtalAB Pro MM007 DW diffractometer with graphite monochromated Cu Kα radiation (*λ* = 1.54184 Å). Structures were solved using direct methods and then refined with SHELXL-2014 and Olex2 for convergence^36–38^, where all the nonhydrogen atoms were refined anisotropically during the final cycles, and all hydrogen atoms of the organic molecule were placed according to geometrical considerations. The disorder in the heavy atoms of cluster [Nb_2_Cp_2_Bi_5_]^2−^ was solved using the split SAME process in Olex 2.

### ESI–MS investigations

Negative-ion-mode ESI–MS of the DMF solutions of crystals of **1** and **2** were measured on an LTQ linear ion trap spectrometer (Agilent Technologies ESI-TOF-MS 6230). The spray voltage was 5.48 kV, and the capillary temperature was maintained at 300 °C. The capillary voltage was 30 V. The samples were made inside a glovebox under a nitrogen atmosphere, then rapidly transferred to the spectrometer in an airtight syringe by direct infusion with a Harvard syringe pump at 0.2 ml min^−1^.

### EDX spectroscopy analysis

EDX analyses of compounds **1** and **2** were performed using a scanning electron microscope (FE-SEM, JEOL JSM-7800F). Data acquisition was performed with an acceleration voltage of 15 kV and an accumulation time of 60 s.

### Computational details

Geometry optimizations were performed with the Gaussian 16 program^39^. The calculations were carried out for all molecules via the BP86^40,41^ functional with the def2-TZVPP^42^ basis set. Vibrational frequency calculations were performed for all stationary points to identify whether they were local minima (no imaginary frequencies). The natural charges were computed using the NBO6 program^43,44^. The Laplacian of the electron density was estimated via Bader’s QTAIM^45^ method with the AIMALL^46^ program. To understand the chemical bonding of [V_2_Cp_2_Sb_5_]^2−^ and [Nb_2_Cp_2_Bi_5_]^2−^ species, we carried out electron localization analysis at the same level of theory using the AdNDP method as implemented in the AdNDP 2.0 code. AdNDP has been shown to be insensitive to the level of theory or the basis set used^47^. The AdNDP analysis figures were prepared using Multiwfn^48^. Graphical structures were visualized with VMD software^45^. The NICS analysis^49^ was completed using the continuous set of gauge transformation method^50^.

The bonding situation was analysed via EDA^51,52^ together with the NOCV^53,54^ method via the ADF 2019.103 program package^55,56^. The EDA-NOCV calculations were carried out at the BP86-D3(BJ)/TZ2P level^57^ with BP86/def2-TZVPP optimized geometries. In this analysis, the intrinsic interaction energy (Δ*Ε*_int_) between two fragments can be divided into four energy components as follows:

1 $${\Delta E}_{\mathrm{int}}={\Delta E}_{{\rm{elstat}}}+{\Delta E}_{{\rm{Pauli}}}+{\Delta E}_{{\rm{orb}}}+{\Delta E}_{{\rm{disp}}}$$

The electrostatic Δ*E*_elstat_ term represents the quasiclassical electrostatic interaction between the unperturbed charge distributions of the prepared fragments, and the Pauli repulsion Δ*E*_Pauli_ corresponds to the energy change associated with the transformation from the superposition of the unperturbed electron densities of the isolated fragments to the wavefunction^58^, which properly obeys the Pauli principle through explicit antisymmetrization and renormalization of the production wavefunction. The orbital term Δ*E*_orb_ can be further decomposed into contributions from each irreducible representation of the point group of the interacting system as follows:

2 $${\Delta E}_{{\rm{orb}}}=\sum _{r}\Delta {E}_{{{r}}}$$

The addition of Δ*E*_prep_ to the intrinsic interaction energy Δ*E*_int_ gives the total energy Δ*E*, which has the opposite sign to the bond dissociation energy *D*_e_ (equation (3)):

3 $${\Delta {E}(-{{{D}}}_{{\rm{e}}})}={\Delta {E}_{\mathrm{int}}+{\Delta E}_{{\rm{prep}}}}$$

The combination of the EDA with NOCV enables the partition of the total orbital interactions into pairwise contributions of the orbital interactions, which is vital for obtaining a complete picture of the bonding. The charge deformation Δ*ρ*_*k*_(*r*), resulting from the mixing of the orbital pairs *ψ*_*k*_(*r*) and *ψ*_−*k*_(*r*) of the interacting fragments, represents the amount and shape of the charge flow due to orbital interactions (equation (4)), and the associated energy term Δ*E*_orb_ represents the amount of stabilizing orbital energy originating from such interactions (equation (5)):

4 $${\Delta {\rho }_{{\rm{orb}}}({{r}})}={\sum _{{{k}}}\Delta {\rho }_{{{k}}}({{r}})={v}_{{{k}}}\left[-{\psi }_{-k}^{2}({{r}})+{\psi }_{k}^{2}({{r}})\right]}$$

5 $${\Delta {E}_{{\rm{orb}}}}={\sum _{{{k}}}\varDelta {{E}_{{{k}}}^{{\rm{orb}}}}}={\mathop{\sum }\limits_{{{k}}=1}^{{{N}}/2}{\nu }_{{{k}}}\left[-{F}_{-{{k}},\,{{k}}}^{{\rm{TS}}}+{F}_{{{k}},\,{{k}}}^{{\rm{TS}}}\right]}$$

More details about the EDA-NOCV method and its application are given in recent review articles^24,59^.

## Online content

Any methods, additional references, Nature Portfolio reporting summaries, source data, extended data, supplementary information, acknowledgements, peer review information; details of author contributions and competing interests; and statements of data and code availability are available at 10.1038/s41557-025-01765-4.

## Supplementary information

Supplementary Information Supplementary Figs. 1–20, Discussion and Tables 1–6.

Supplementary Data 1 Crystallographic data for compound **1** (CCDC 2342591).

Supplementary Data 2 Crystallographic data for compound **2** (CCDC 2342488).

Supplementary Data 3 Coordinates for compound **1**.

Supplementary Data 4 Coordinates for compound **2**.

## Data Availability

All the data produced or analysed in this study are available within the main Article ot its Supplementary files. X-ray data are available free of charge from the Cambridge Crystallographic Data Centre under reference nos. CCDC 2342591 (**1**) and 2342488 (**2**). All other experimental, spectroscopic, crystallographic and computational data are included in the Supplementary Information.

## Extended data

**Extended Data Table 1:** Calculated NICS values at BP86-D3(BJ)/def2-TZVPP

	NICS(0)	NICS(1)_ZZ_	NICS(-1)_ZZ_
**Free rings**			
Cp^−^ opt	-13.9	-33.9	-33.9
Cp^−^ frozen^a^	-13.8	-34.1	-33.3
Sb_5_^−^ opt	-13.5	-26.0	-26.0
Sb_5_^−^ frozen	-10.2	-17.3	-17.3
Bi_5_^−^ opt	-10.7	-7.2	-7.2
Bi_5_^−^ frozen	-6.1	-6.8	-6.8
**[Cp-V(cyclo-Sb**_**5**_ **)V-Cp]** ^**2-**^			
Cp^−^	-14.5	-14.3	315.7
Sb_5_^−^	18.1	1714.2	1714.2
Cp^−^	-14.5	315.7	-14.3
**[Cp-Nb(cyclo-Bi**_**5**_ **)Nb-Cp]** ^**2-**^			
Cp^−^	-14.9	-18.9	104.2
Bi_5_^−^	12.3	461.5	461.5
Cp^−^	-14.9	104.2	-18.9

^a^Using the frozen geometry in [Cp-V(*cyclo*-Sb_5_)V-Cp]^2-^. The Cp^-^ fragments of the Bi_5_ complex gives similar values.

**Extended Data Table 2:** The calculated NBO charges (*q*) Wiberg bond orders WBO and Mayer bond orders MBO of [V_2_Cp_2_Sb_5_]^2-^ and (in parentheses) [Nb_2_Cp_2_Bi_5_]^2–^ at the BP86-D3(BJ)/def2-TZVPP level

C_2v_	q		WBO	MBO
[V_2_Cp_2_Sb_5_]^2-^ ([Nb_2_Cp_2_Bi_5_]^2–^)				
Sb_5_ (Bi_5_)	0.200 (-0.142)	Sb1-Sb2 (Bi1-Bi2)	0.52 (0.50)	0.75 (0.47)
		Sb2-Sb3 (Bi2-Bi3)	0.52 (0.50)	0.75 (0.47)
		Sb3-Sb2' (Bi3-Bi3’)	0.66 (0.63)	0.87 (0.60)
		Sb2'-Sb1'(Bi2’-Bi1’)	0.75 (0.73)	0.94 (0.69)
		Sb1'-Sb1(Bi1’-Bi1)	0.66 (0.63)	0.87 (0.60)
V_2_ (Nb_2_)	-1.200 (-1.176)	V1/V2-Sb1 (Nb1/Nb2-Bi1)	0.66 (0.65)	0.41 (0.75)
		V1/V2-Sb2 (Nb1/Nb2-Bi2)	0.71 (0.70)	0.46 (0.81)
		V1/V2-Sb3 (Nb1/Nb2-Bi3)	0.66 (0.65)	0.41 (0.75)
		V1/V2-Sb1' (Nb1/Nb2-Bi1‘)	0.58 (0.56)	0.34 (0.65)
		V1/V2-Sb2' (Nb1/Nb2-Bi2‘)	0.58 (0.56)	0.34 (0.65)
		V1-V2 (Nb1-Nb2)	0.63 (0.68)	0.57 (0.98)
Cp_2_	-1.000 (-0.682)	C1-H1	0.90 (0.90)	0.98 (0.97)
		C-V (C-Bi)	0.26 (0.26)	0.30 (0.37)
