# Author Correction: Synthesis of triple-decker sandwich compounds featuring a M–M bond through cyclo-Bi_5_ and cyclo-Sb_5_ rings

**DOI:** 10.1038/s41557-025-01825-9

**Published:** 2025-04-15

**Authors:** Yu-He Xu, Xing Yang, Ya-Nan Yang, Lili Zhao, Gernot Frenking, Zhong-Ming Sun

**Affiliations:** 1 State Key Laboratory of Elemento-Organic Chemistry, Tianjin Key Lab of Rare Earth Materials and Applications, School of Material Science and Engineering, Nankai University, Tianjin, China; 2 State Key Laboratory of Materials-Oriented Chemical Engineering, School of Chemistry and Molecular Engineering, Nanjing Tech University, Nanjing, China; 3 College of Chemistry and Molecular Sciences, Henan University, Kaifeng, China; 4 Fachbereich Chemie, Philipps-Universität Marburg, Marburg, Germany; 5 Donostia International Physics Center (DIPC), Donostia, Spain

**Keywords:** Chemical bonding, Synthetic chemistry methodology

Correction to: *Nature Chemistry* 10.1038/s41557-025-01765-4, published online 18 March 2025.

In the version of the article initially published, in the Abstract, the phrase “E_5_ rings (E = Sb, Nb)” should have been “E_5_ rings (E = Sb, Bi)”. Additionally, the author names were incorrect in ref. 11 which has now been amended to “Yong, L., Hoffmann, S. D., Fässler, T. F., Riedel, S. & Kaupp, M. [Pb_5_{Mo(CO)_3_}_2_]^4−^: a complex containing a planar Pb5 unit. *Angew. Chem. Int. Ed*. **44**, 2092–2096 (2005)” in the HTML and PDF versions of the article.

