# Supplementary material for: Synthesis of triple-decker sandwich compounds featuring a M–M bond through cyclo-Bi5 and cyclo-Sb5 rings
Source: Nat Chem. 2025 Mar 18;17(4):556–63. doi: 10.1038/s41557-025-01765-4 (PMC11964928; doi:10.1038/s41557-025-01765-4)
Supplement: Supplementary file 1 — Supplementary Figs. 1–20, Discussion and Tables 1–6. [file 41557_2025_1765_MOESM1_ESM.pdf]

nature chemistry

Supplementary information https://doi.org/10.1038/s41557-025-01765-4

Synthesis of triple-decker sandwich compounds featuring a M–M bond through cyclo-Bi_5 and cyclo-Sb_5 rings

In the format provided by the authors and unedited

Table of Contents

1. Crystallographic Supplementation 2
2. ESI-MS Studies 5
3. Energy Dispersive X-ray (EDX) Spectroscopic Analysis 7
4. Computational Details 8

1. Crystallographic Supplementation

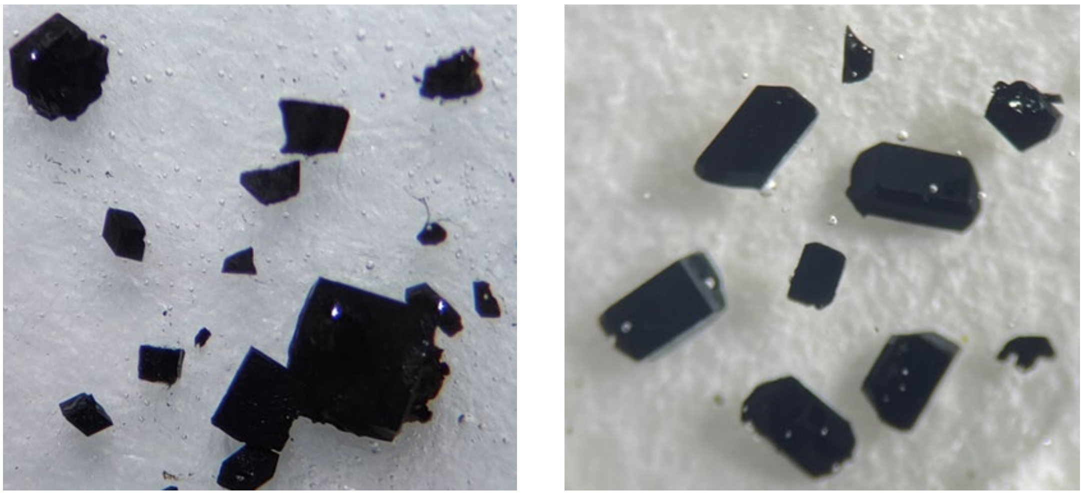

(1) (2)

Supplementary Figure 1. Crystals of (**1**) [K(18-crown-6)]$_{2.5}$[$V_2Cp_2Sb_5$] ·0.5Cp·3Py, (**2**) [K(2.2.2-crypt)]$_2$[$Nb_2Cp_2Bi_5$] ·0.5en·tol dispersed in silicon oil, respectively.

Supplementary Table 1. X-ray measurements and structure solutions of [K(18-crown-6)]$_{2.5}$[$V_2Cp_2Sb_5$]·0.5Cp·3Py (**1**) and [K(2.2.2-crypt)]$_2$[$Nb_2Cp_2Bi_5$]·0.5en·tol (**2**).

Compound	**1**	**2**
Empirical formula	$C_{125}H_{185}K_5N_7O_{30}Sb_{10}V_4$	$C_{54}H_{94}O_{12}N_5K_2Nb_2Bi_5$
Formula weight	3882.55	2314.26
Crystal system	Monoclinic	Triclinic
Space group	*C*2/m	*P*-1
a /Å	18.5786(2)	13.2882(2)
b /Å	17.5274(2)	16.4289(2)
c /Å	24.1019(2)	18.0263(2)
α/°	90	88.2740(10)
β/°	94.2120(10)	89.5600(10)
γ/°	90	68.8940(10)
V /Å3	7827.21(14)	3669.65(9)
Z	2	2
ρ_{calc} /g·cm-3	1.647	2.094
μ(Cu*K*α) /mm-1	17.061	12.411
F(000)	3842.0	2168.0
2*Θ* range /°	7.692 to 151.614	6.944 to 49.994
Reflections collected / unique	21333/15919	76330/46796
Data / restraints / parameters	8109/777/772	12905/28/730
R_1/wR_2 (I>2σ(I))a	0.0914; 0.2512	0.0218; 0.0455
R_1/wR_2 (all data)	0.0946; 0.2549	0.0237; 0.0462
GooF (all data)b	1.058	1.025
Data completeness	0.962	0.997
Max. peak/hole /e-·Å-3	1.92/-2.18	2.21/-1.41
CCDC	2342591	2342488

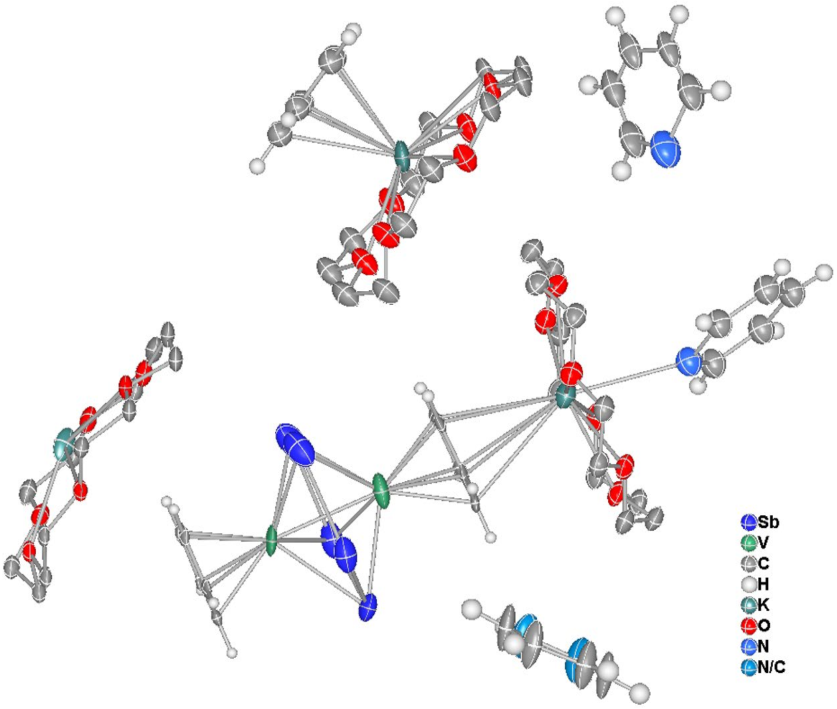

Supplementary Figure 2. Asymmetric unit of compound **1**. Thermal ellipsoids are set at 50%.

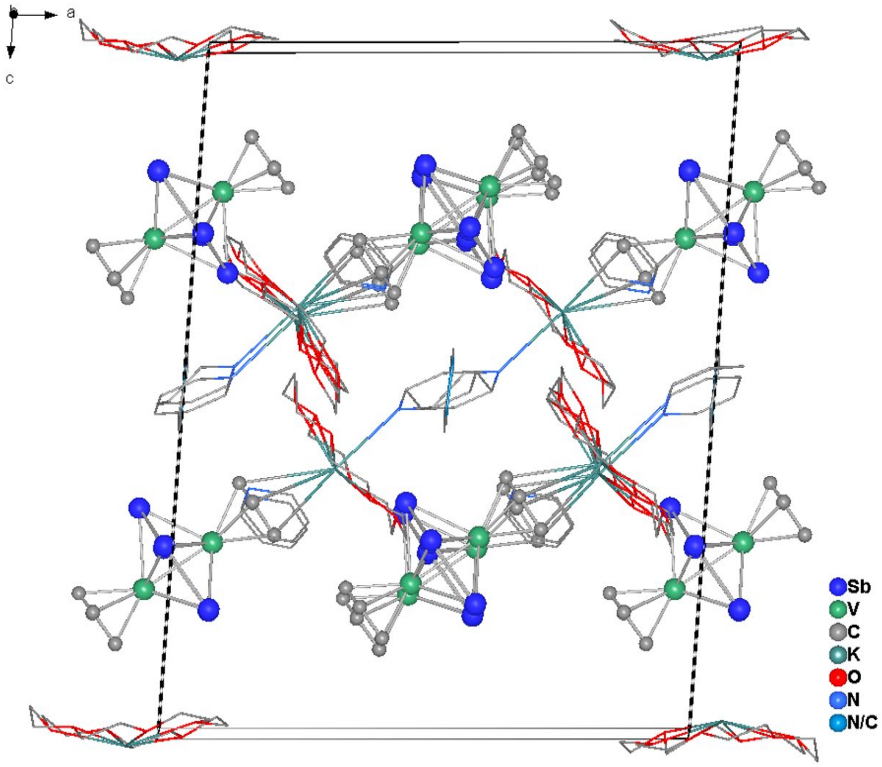

Supplementary Figure 3. Unit cell of compound **1**.

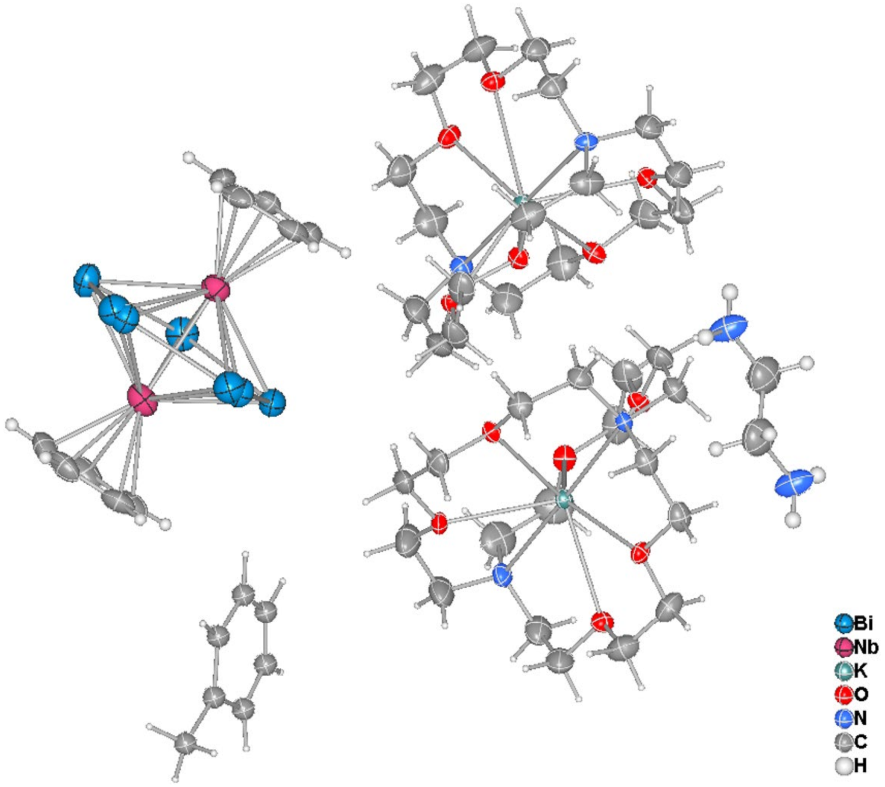

Supplementary Figure 4. Asymmetric unit of compound **2**. Thermal ellipsoids are set at 50%.

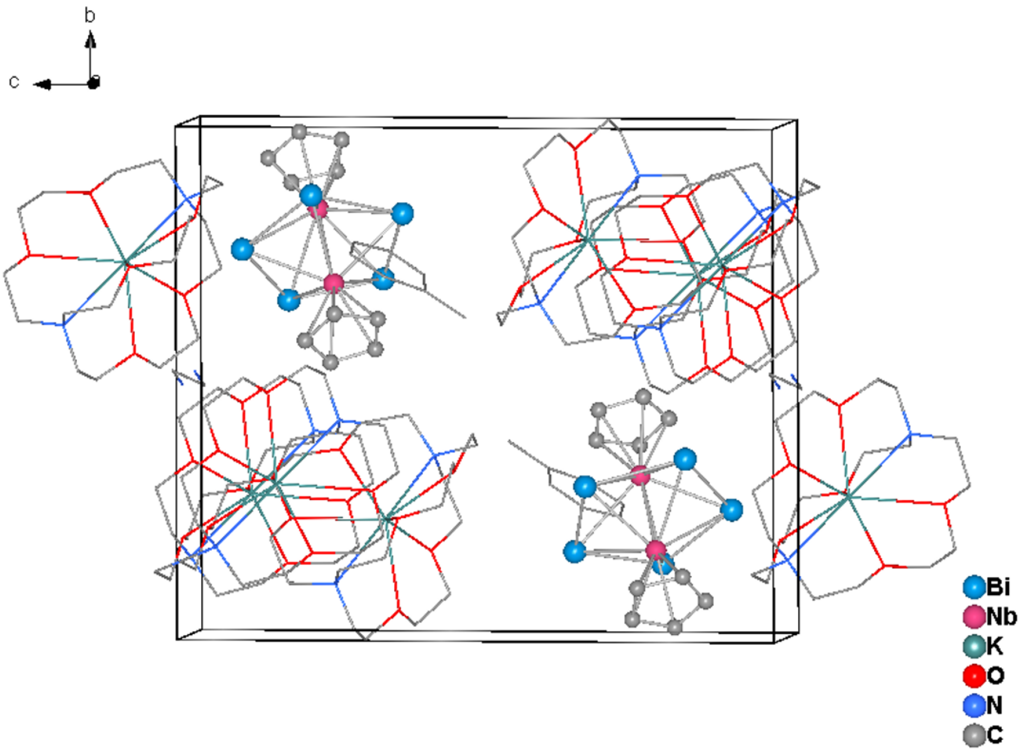

Supplementary Figure 5. Unit cell of compound **2**.

Responses to B-level alerts in the CIF of compound $[K(2.2.2\text{-crypt})]_2[Nb_2Cp_2Bi_5]\cdot0.5en\cdot tol$ (**2**)
PROBLEM: Missing # of FCF Reflection(s) Below Theta(Min). 36 Note
RESPONSE: Low-angle reflections rejected because of beam stop and high background scattering.

2. ESI-MS Studies

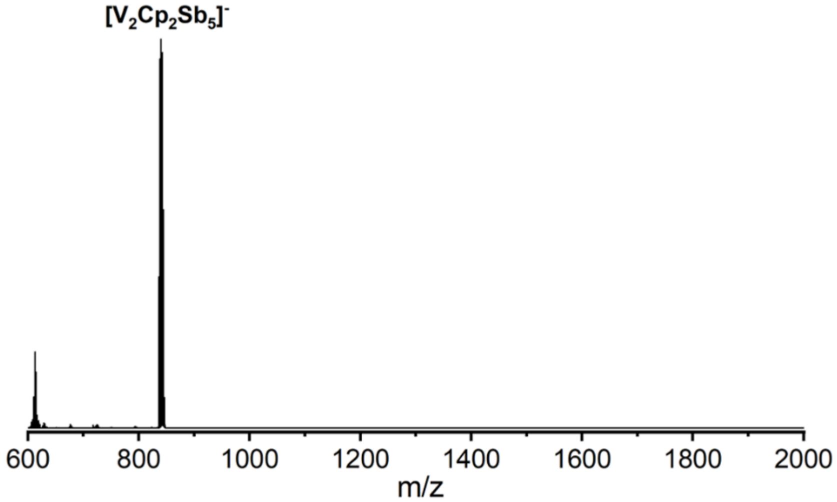

Supplementary Figure 6. Overview ESI (-) mass spectrum for compound **1**. The sample was obtained from a freshly dissolved crystalline compound **1** in MeCN.

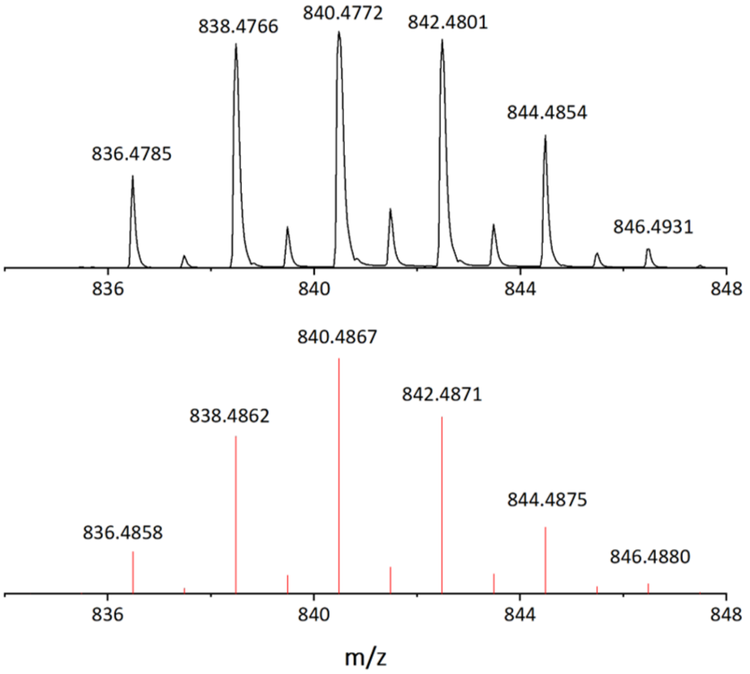

Supplementary Figure 7. Measured (top) and simulated (bottom) spectrum of the fragment [$V_2Cp_2Sb_5$]$^-$.

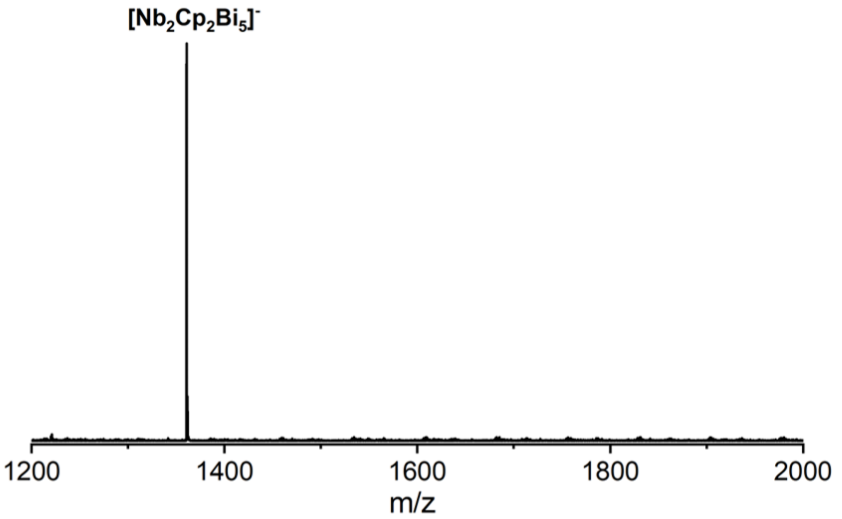

Supplementary Figure 8. Overview ESI (-) mass spectrum for compound **2**. The sample was obtained from a freshly dissolved crystalline compound **2** in MeCN.

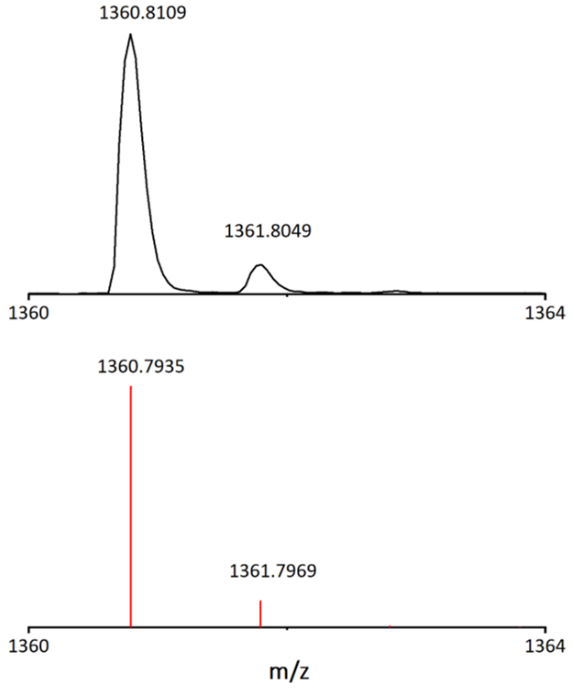

Supplementary Figure 9. Measured (top) and simulated (bottom) spectrum of the fragment $[Nb_2Cp_2Bi_5]^-$.

3. Energy Dispersive X-ray (EDX) Spectroscopic Analysis

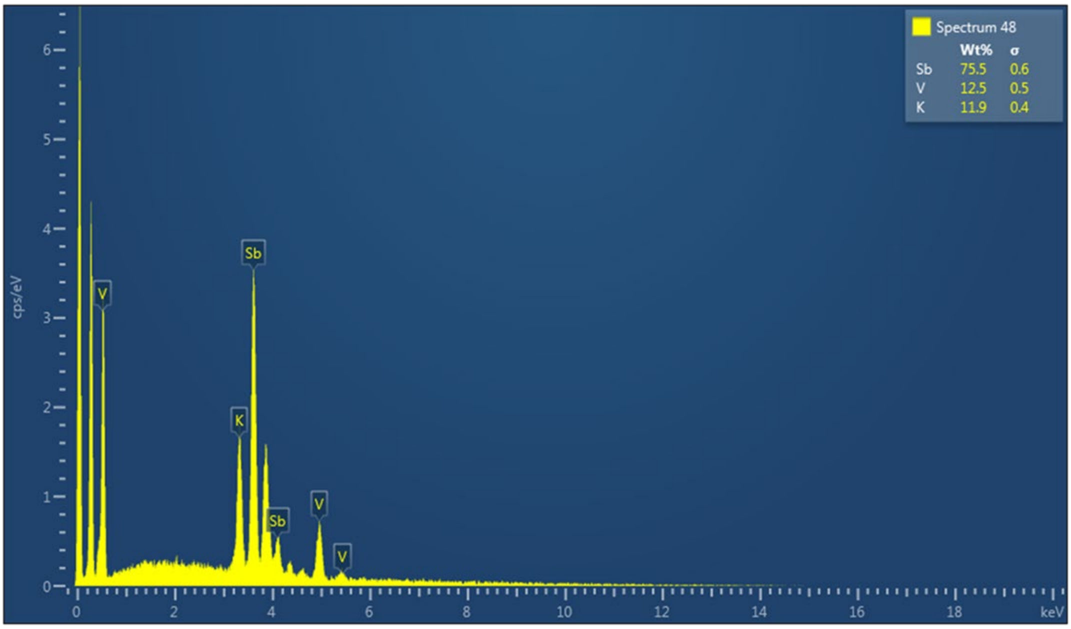

Element	Line type	wt%	σ	Experimental / Calculated Atom %
K	K series	11.9	0.4	26.01/22.22
V	L series	12.5	0.5	20.97/22.22
Sb	L series	75.5	0.6	53.02/55.56

Supplementary Figure 10. EDX analysis of compound **1**.

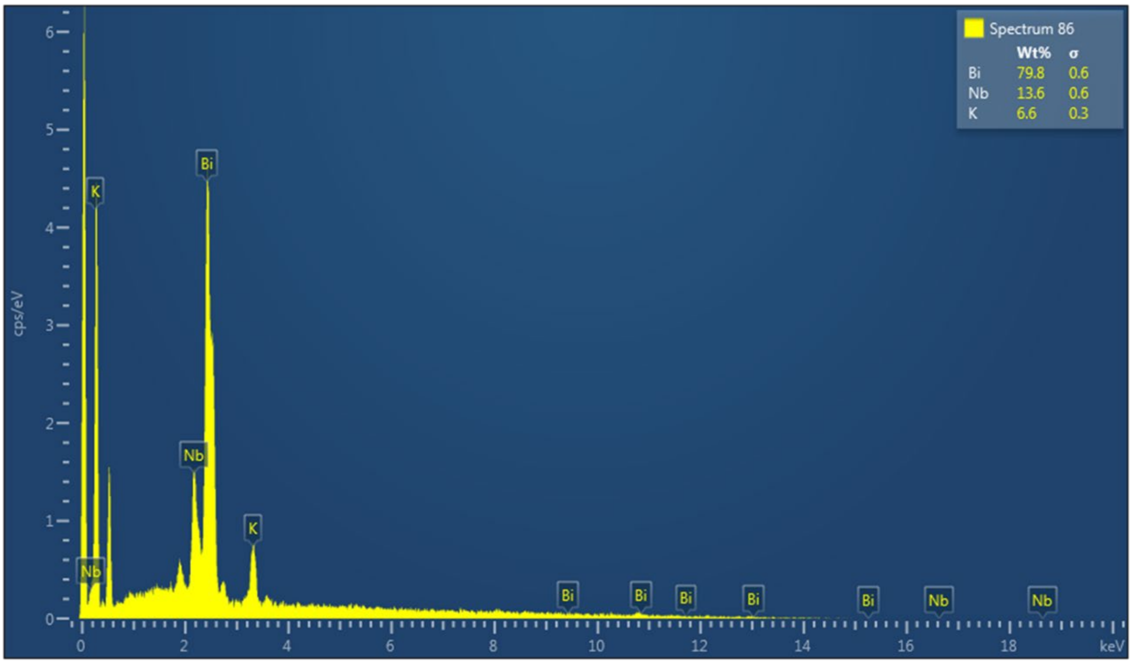

Element	Line type	wt%	σ	Experimental / Calculated Atom %
K	K series	6.6	0.3	24.21/22.22
Nb	L series	13.6	0.6	21.00/22.22
Bi	L series	79.8	0.6	54.78/55.56

Supplementary Figure 11. EDX analysis of compound **2**.

4. Computational Details

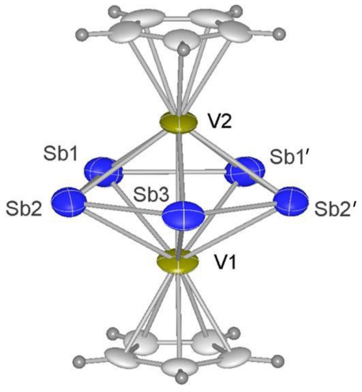

BP86-D3(BJ)/def2-TZVPP//[crystal]

Sb1-Sb1' = 2.789[2.777]
Sb1-Sb2 = 2.822[2.823]
Sb2-Sb3 = 2.888[2.867]
V1-Sb1 = 2.847[2.845]
V1-Sb2 = 2.781[2.793]
V1-Sb3 = 2.745[2.775]
V1-V2 = 2.822[2.900]
∠Sb1'-Sb1-Sb2 = 109.0[109.0]
∠Sb2-Sb3-Sb2' = 106.4[106.4]
∠V2-Sb1-V1 = 59.4[61.5]
∠V2-Sb2-V1 = 61.0[62.1]
∠V2-Sb3-V1 = 61.9[63.1]

Spin density

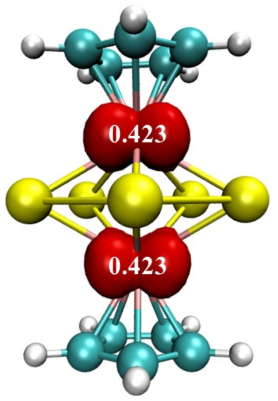

$[V_2Cp2Sb5]^{2-}$ (C2v)

$\Delta E(^2B_1)$ = 0.0 kcal/mol $\Delta E(^4A1)$ = 17.5 kcal/mol

(a)

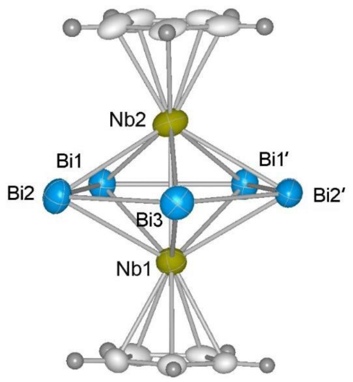

BP86-D3(BJ)/def2-TZVPP//[crystal]

Bi1'-Bi1 = 2.979[3.004]
Bi1-Bi2 = 3.026[3.007]
Bi2-Bi3 = 3.114[3.136]
Nb1-Bi1 = 3.034[2.974]
Nb1-Bi2 = 2.966[2.958]
Nb1-Bi3 = 2.935[2.954]
Nb1-Nb2 = 2.949[2.954]
∠Bi1'-Bi1-Bi2 = 109.2[109.1]
∠Bi2-Bi3-Bi2' = 106.0[105.1]
∠Bi1-Bi2-Bi3 = 107.8[108.0]
∠Nb2-Bi1-Nb1 = 58.2[69.6]
∠Nb2-Bi2-Nb1 = 59.6[59.8]
∠Nb2-Bi3-Nb1 = 60.3[61.0]

Spin density

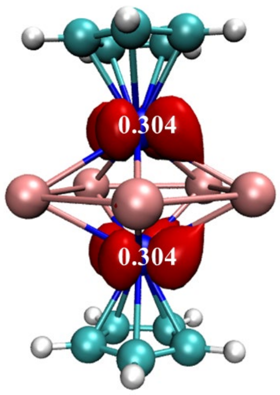

$[Nb_2Cp2Bi5]^{2-}$ (C2v)

ΔE(doublet) = 0.0 kcal/mol $\Delta E(^4A1)$ = 21.0 kcal/mol

(b)

Supplementary Figure 12. Optimized geometry and spin density distribution of (a) $[V_2Cp_2Sb_5]^{2-}$ and (b) $[Nb_2Cp_2Bi_5]^{2-}$ at the BP86-D3(BJ)/def2-TZVPP level. The most important calculated [experimental] distances and angles are given in Å and in degrees, respectively.

Supplementary Table 2. Electron density ($\rho_{(r)}$), Laplacian ($\nabla^2\rho_{(r)}$), Total energy ($H_{(r)}$) values from QTAIM analysis of $[V_2Cp_2Sb_5]^{2-}$ and $[Nb_2Cp_2Bi_5]^{2-}$ at the BP86-D3(BJ)/def2-TZVPP level.

	ρ(BCP)	$\nabla^2\rho$(BCP)	H(BCP)
	$[V_2Cp_2Sb_5]^{2-}$		
Sb1-Sb2	0.047	0.035	-0.010
Sb1-Sb3	0.047	0.035	-0.010
V4-V5	0.045	0.003	-0.012
Sb1-V4	0.048	0.035	-0.014
Sb2-V4	0.044	0.038	-0.012
Sb3-V4	0.044	0.038	-0.012
Sb1-V5	0.048	0.035	-0.014
Sb2-V5	0.044	0.038	-0.012
Sb3-V5	0.044	0.038	-0.012
Sb3-Sb6	0.051	0.033	-0.013
V4-Sb6	0.038	0.046	-0.009
V5-Sb6	0.038	0.046	-0.009
Sb2-Sb23	0.051	0.033	-0.013
V4-Sb23	0.038	0.046	-0.009
V5-Sb23	0.038	0.046	-0.009
Sb6-Sb23	0.053	0.032	-0.014
	$[Nb_2Cp_2Bi_5]^{2-}$		
Bi1-Bi2	0.036	0.046	-0.005
Bi1-Bi3	0.036	0.046	-0.005
Nb4-Nb5	0.056	-0.004	-0.017
Bi1-Nb4	0.046	0.043	-0.010
Bi2-Nb4	0.042	0.044	-0.009
Bi3-Nb4	0.042	0.044	-0.009
Bi1-Nb5	0.046	0.043	-0.010
Bi2-Nb5	0.042	0.044	-0.009
Bi3-Nb5	0.042	0.044	-0.009
Bi2-Bi6	0.040	0.050	-0.006
Nb4-Bi6	0.037	0.046	-0.007
Nb5-Bi6	0.037	0.046	-0.007
Bi3-Bi7	0.040	0.050	-0.006
Nb4-Bi7	0.037	0.046	-0.007
Nb5-Bi7	0.037	0.046	-0.007
Bi6-Bi7	0.043	0.053	-0.007

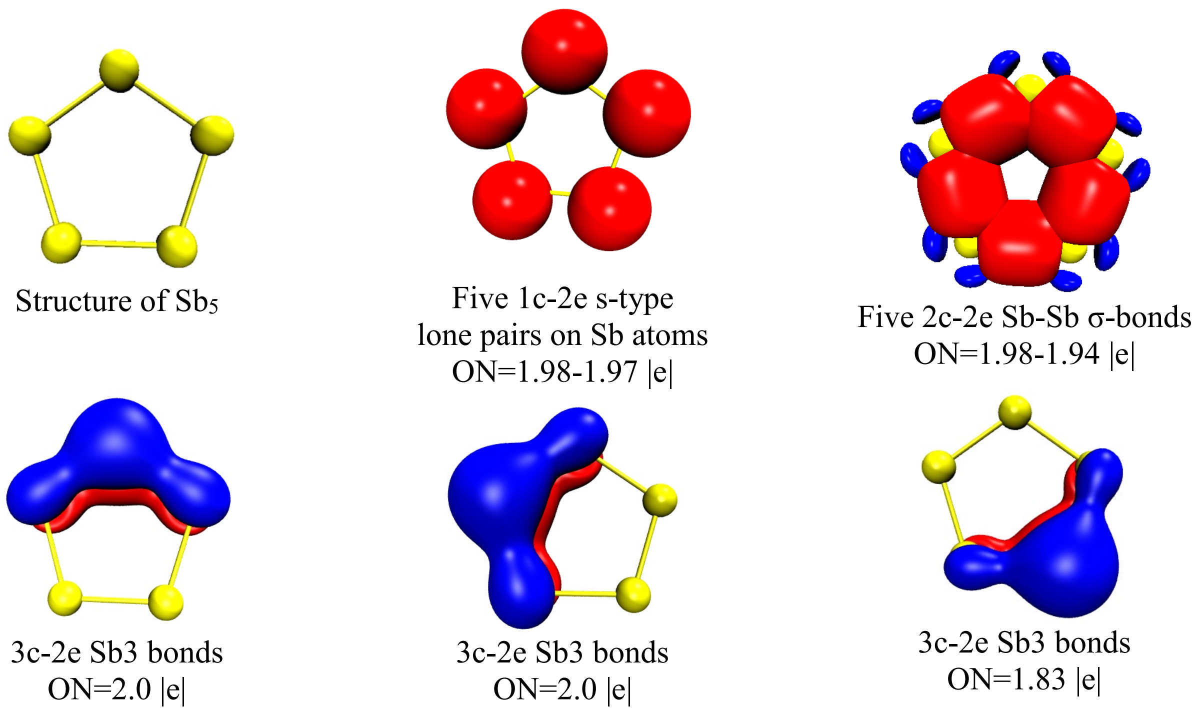

Supplementary Figure 13. Chemical bonding pattern of Sb_5^- from AdNDP analysis. ON denotes occupation numbers (equal to 2.00 |e| in an ideal case). Lines between atoms are presented for visualization and do not necessarily correspond to 2c–2e bonds. Sb-atoms are dark yellow.

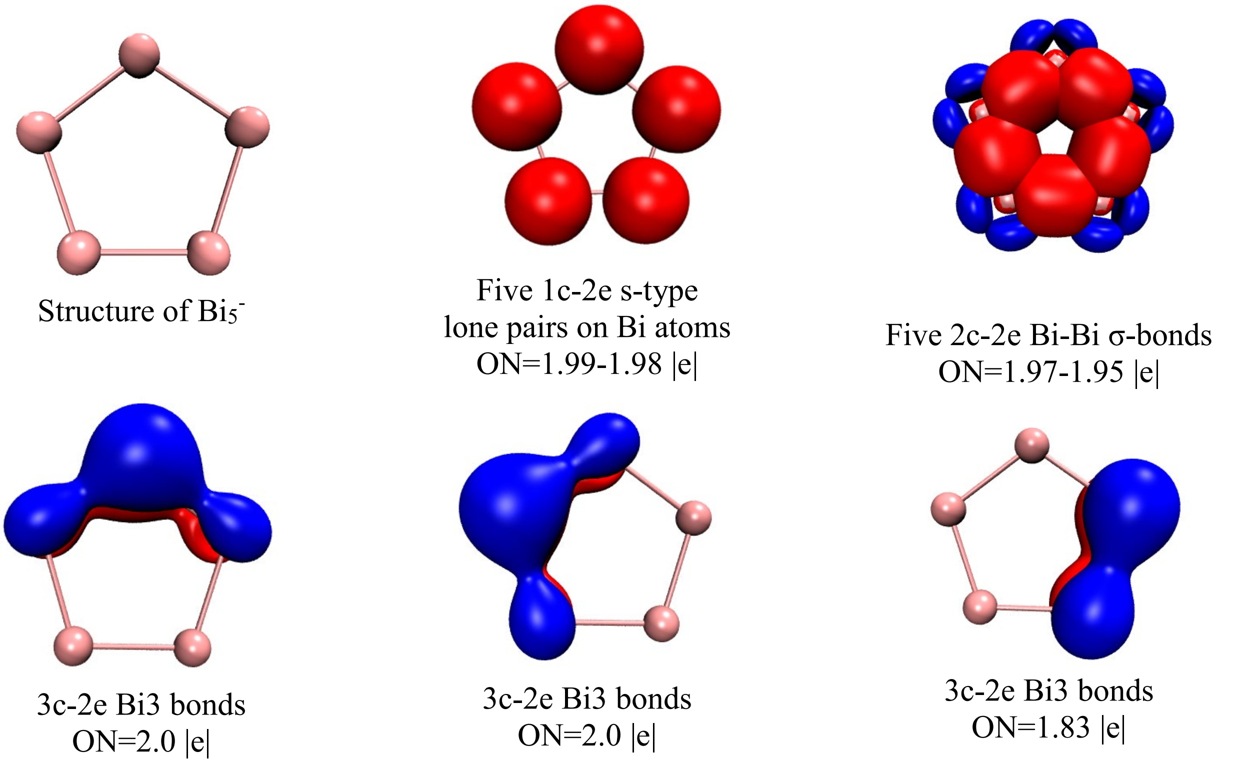

Supplementary Figure 14. Chemical bonding pattern of Bi_5^- from AdNDP analysis. ON denotes occupation numbers (equal to 2.00 |e| in an ideal case). Lines between atoms are presented for visualization and do not necessarily correspond to 2c–2e bonds. Bi-atoms are pink.

$[V_2Cp_2Sb_5]^{2-}$		$[Nb_2Cp_2Bi_5]^{2-}$	
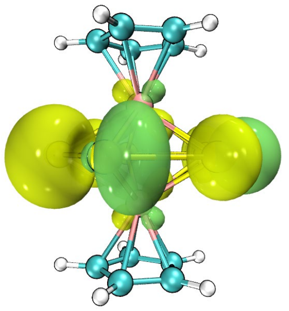<br>LUMO+1α<br>(ε=4.189 eV)	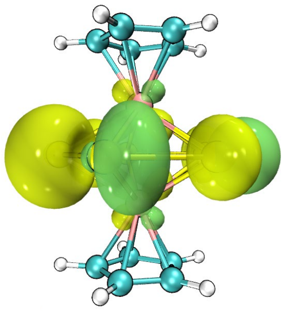<br>LUMO+1β<br>(ε=4.268 eV)	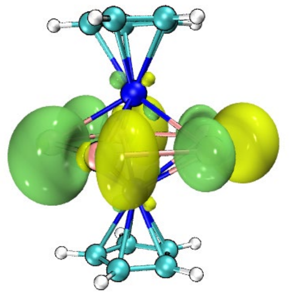<br>LUMO+1α<br>(ε=3.955 eV)	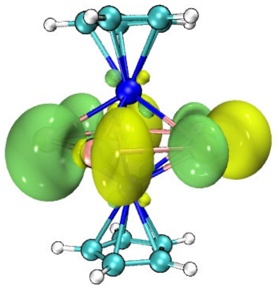<br>LUMO+1β<br>(ε=3.866 eV)
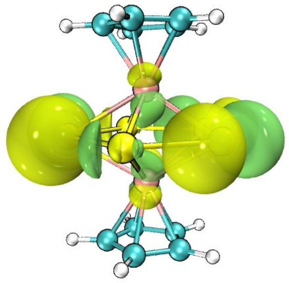<br>LUMO-α<br>(ε=4.167 eV)	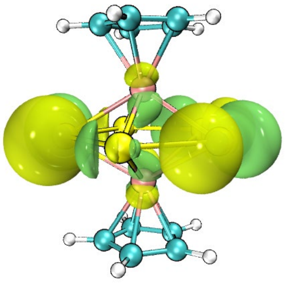<br>LUMO-β<br>(ε=4.244 eV)	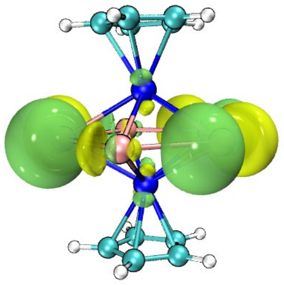<br>LUMO-α<br>(ε=3.840 eV)	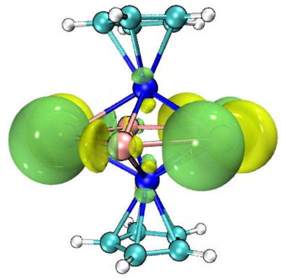<br>LUMO-β<br>(ε=3.926 eV)
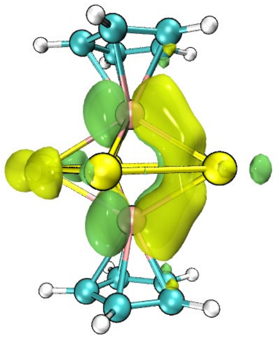<br>SOMO-α<br>(ε=2.904 eV)	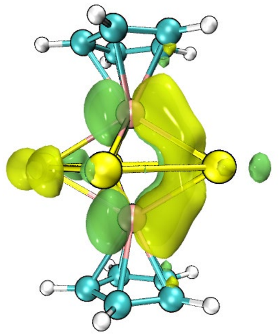<br>SOMO-β<br>(ε=3.350 eV)	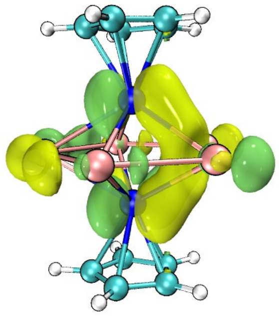<br>SOMO-α<br>(ε=2.706 eV)	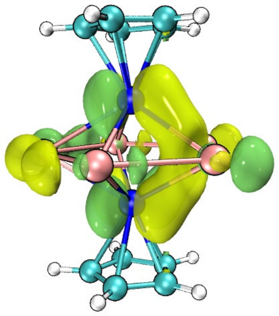<br>SOMO-β<br>(ε=2.983 eV)
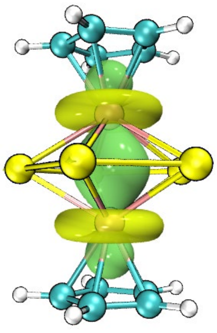<br>HOMO-α<br>(ε=2.861 eV)	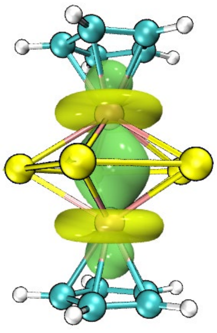<br>HOMO-β<br>(ε=3.047 eV)	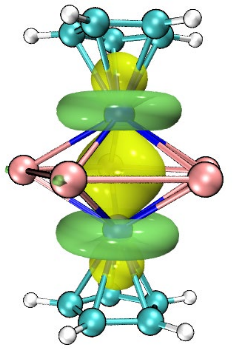<br>HOMO-α<br>(ε=2.697 eV)	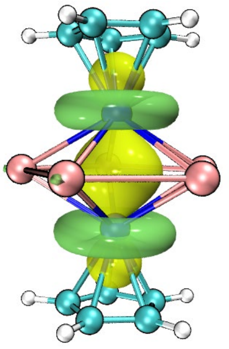<br>HOMO-β<br>(ε=2.797 eV)

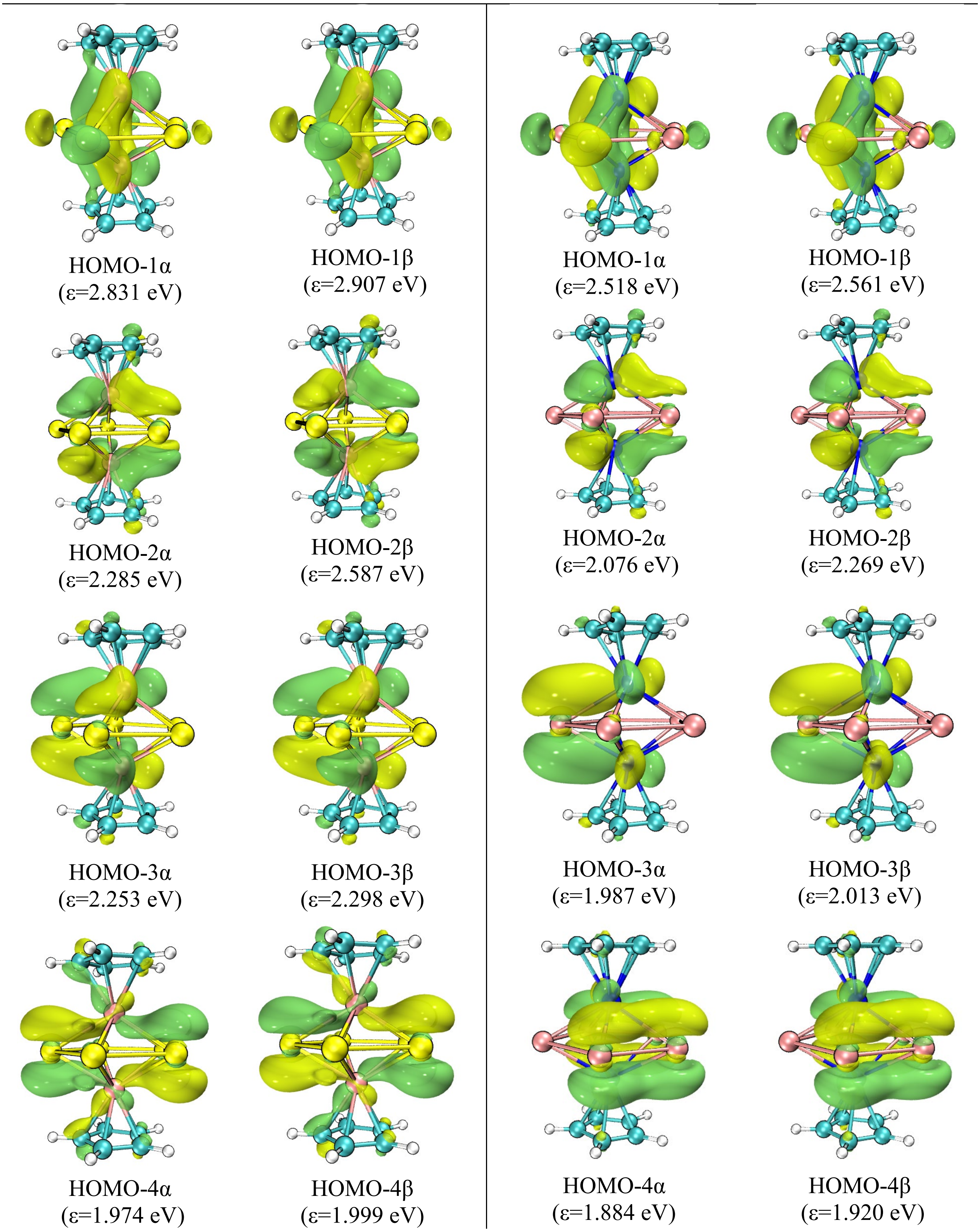
HOMO-1α
(ε=2.831 eV)
HOMO-1β
(ε=2.907 eV)
HOMO-1α
(ε=2.518 eV)
HOMO-1β
(ε=2.561 eV)
HOMO-2α
(ε=2.285 eV)
HOMO-2β
(ε=2.587 eV)
HOMO-2α
(ε=2.076 eV)
HOMO-2β
(ε=2.269 eV)
HOMO-3α
(ε=2.253 eV)
HOMO-3β
(ε=2.298 eV)
HOMO-3α
(ε=1.987 eV)
HOMO-3β
(ε=2.013 eV)
HOMO-4α
(ε=1.974 eV)
HOMO-4β
(ε=1.999 eV)
HOMO-4α
(ε=1.884 eV)
HOMO-4β
(ε=1.920 eV)

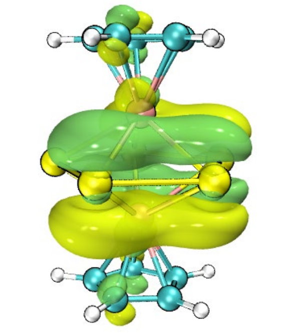

HOMO-5α
(ε=1.964 eV)

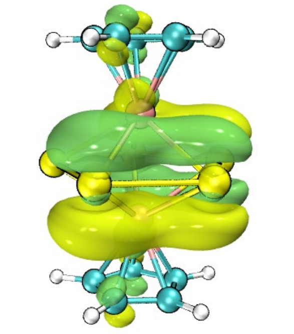

HOMO-5β
(ε=1.991 eV)

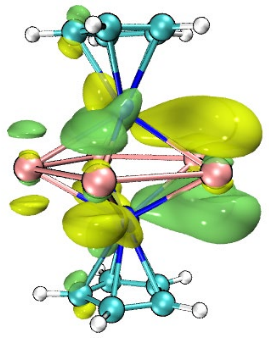

HOMO-5α
(ε=1.878 eV)

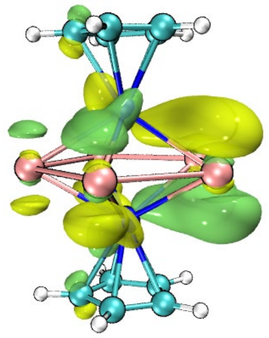

HOMO-5β
(ε=1.911 eV)

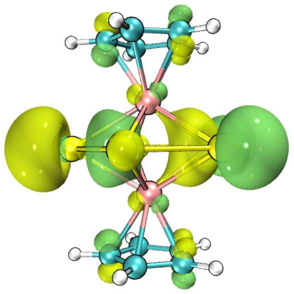

HOMO-6α
(ε=0.884 eV)

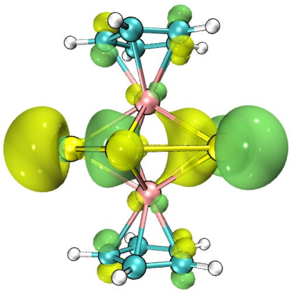

HOMO-6β
(ε=0.935 eV)

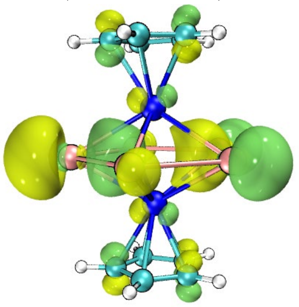

HOMO-6α
(ε=0.838 eV)

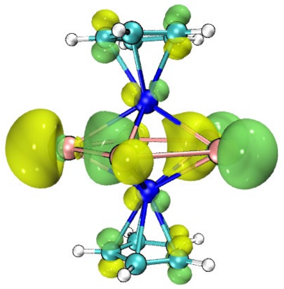

HOMO-6β
(ε=0.898 eV)

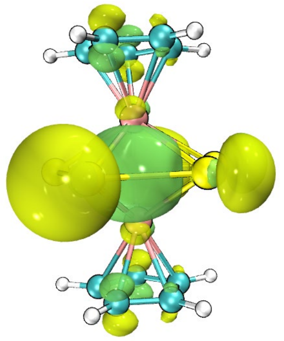

HOMO-7α
(ε=0.876 eV)

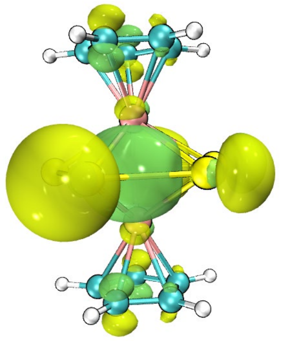

HOMO-7β
(ε=0.932 eV)

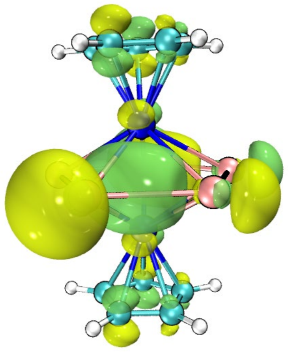

HOMO-7α
(ε=0.828 eV)

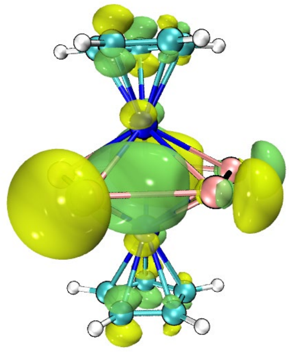

HOMO-7β
(ε=0.896 eV)

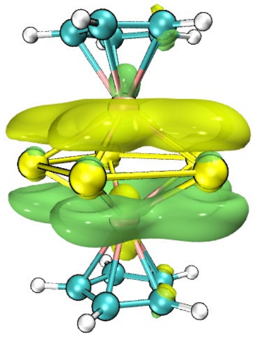

HOMO-8α
(ε=0.626 eV)

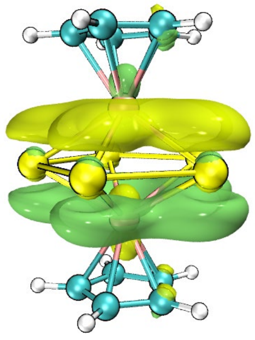

HOMO-8β
(ε=0.675 eV)

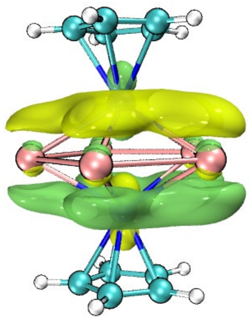

HOMO-8α
(ε=0.530 eV)

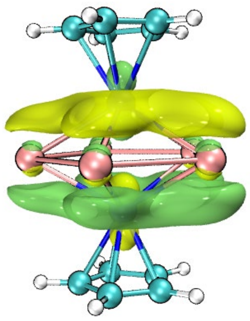

HOMO-8β
(ε=0.595 eV)

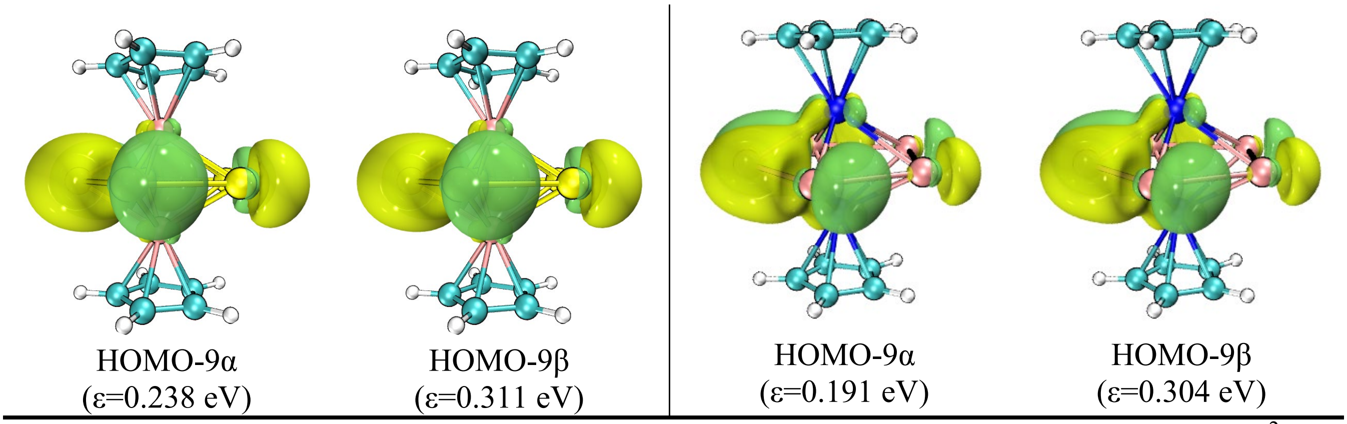

Supplementary Figure 15. Occupied and two lowest vacant MOs of the complexes of $[V_2Cp_2Sb_5]^{2-}$ and $[Nb_2Cp_2Bi_5]^{2-}$ at BP86+(D3BJ)/def2-TZVPP level.

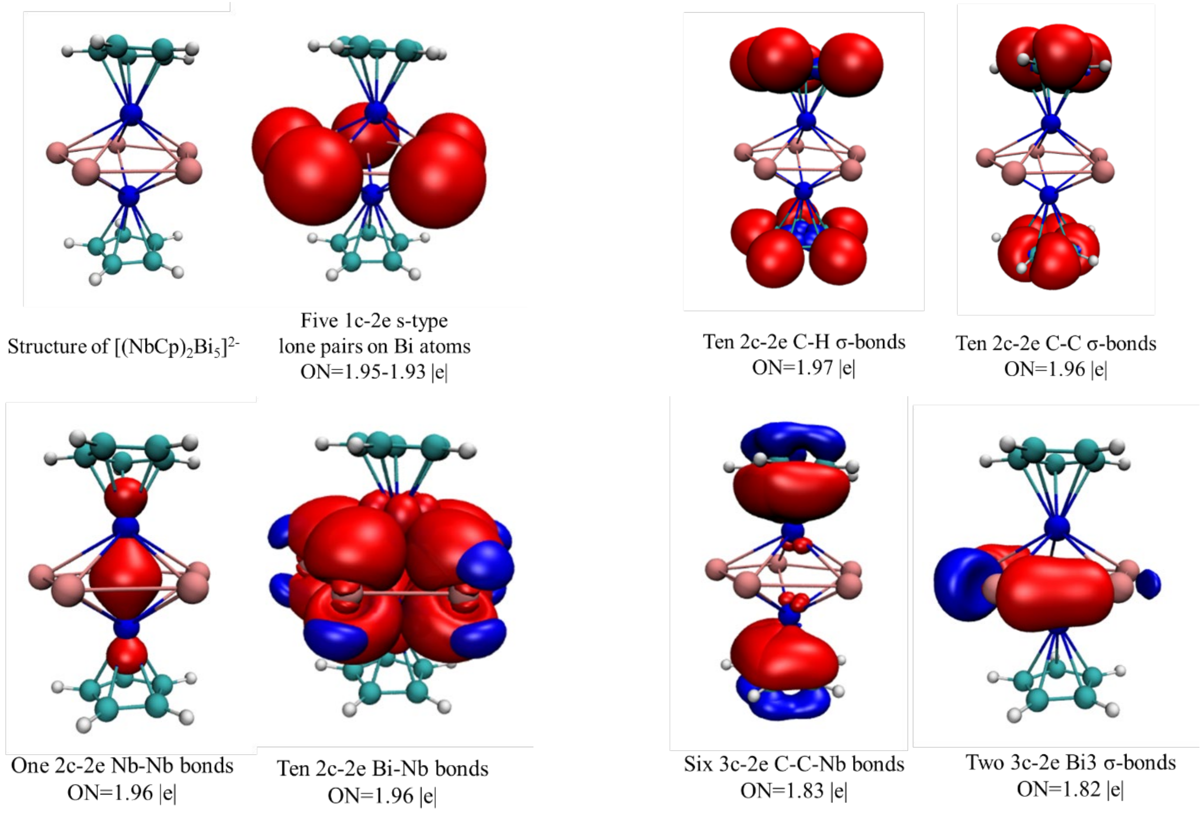

Supplementary Figure 16. Chemical bonding pattern of $[Nb_2Cp_2Bi_5]^{2-}$ from AdNDP analysis. ON denotes occupation numbers (equal to 2.00 |e| in an ideal case). Lines between atoms are presented for visualization and do not necessarily correspond to 2c-2e bonds. Nb-atoms are dark bule, Bi-atoms are pink, C-atoms are cyan and H-atoms are white.

Supplementary Table 3. EDA-NOCV results of $[V_2Cp_2Sb_5]^{2-}$ at the BP86-D3(BJ)/TZ2P level of theory. Fragments are given on table in singlet (S) ([cyclo-Sb_5] and $(VCp)_2$) or Doublet (D) electronic states. Energy values are given in kcal/mol.

Fragments	**[cyclo-Sb_5]$^-$** (S)+ **$(VCp)_2$$^{1-}$** (D)	**[cyclo-Sb_5]$^+$** (S)+ **$(VCp)_2$$^{3-}$** (D)	**[cyclo-Sb_5]$^{3-}$** (D)+ **$(VCp)_2$$^{1+}$** (S)
ΔE_{int}	-308.6	-748.0	-1498.4
ΔE_{Pauli}	682.7	1451.5	793.1
ΔE_{elstat}[a]	-503.8 (50.8 %)	-1290.8 (58.7%)	-809.9 (54.0%)
ΔE_{disp}[a]	-48.9 (4.9%)	-48.9 (2.2%)	-48.9 (3.3%)
ΔE_{orb}[a]	-438.6 (44.3%)	-859.8 (39.1%)	-639.6 (42.7%)
$\Delta E_{orb(1)}$[b]	-89.3 (20.4%)		
$\Delta E_{orb(2)}$[b]	-85.8 (19.6%)		
$\Delta E_{orb(3)}$[b]	-80.7 (18.4%)		
$\Delta E_{orb(4)}$[b]	-65.8 (15.0%)		
$\Delta E_{orb(5)}$[b]	-31.8 (7.3%)		
$\Delta E_{orb(6)}$[b]	-21.7 (4.9%)		
$\Delta E_{orb(7)}$[b]	-21.4 (4.9%)		
$\Delta E_{orb(8)}$[b]	-12.5 (2.8%)		
$\Delta E_{orb(9)}$[b]	-6.2 (1.4%)		
$\Delta E_{orb(10)}$[b]	-6.2 (1.4%)		
$\Delta E_{orb(11)}$[b]	-2.9 (0.7%)		
$\Delta E_{orb(rest)}$[b]	-14.3 (3.2%)		

[a]The values in parentheses give the percentage contribution to the total attractive interactions $\Delta E_{elstat} + \Delta E_{orb} + \Delta E_{disp}$.

[b]The values in parentheses give the percentage contribution to the total orbital interactions ΔE_{orb}.

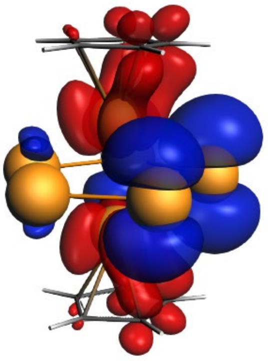

$\Delta E_{orb(1\alpha)}$ = –43.3 (kcal/mol)
$\nu_{1\alpha} = \pm 0.539$

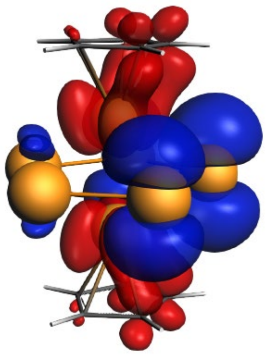

$\Delta E_{orb(1\beta)}$ = –46.0 (kcal/mol)
$\nu_{1\beta} = \pm 0.563$

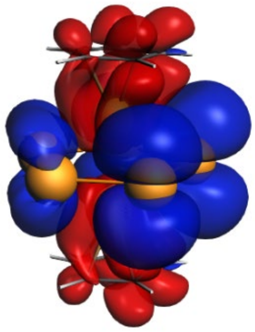

$\Delta E_{orb(1)}$ = –89.3 (kcal/mol)
$\nu_{1} = \pm 1.102$

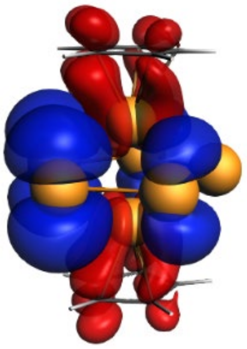

$\Delta E_{orb(2\alpha)}$ = –36.4 (kcal/mol)
$\nu_{2\alpha} = \pm 0.509$

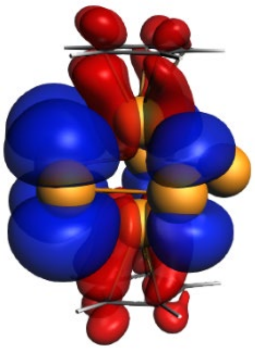

$\Delta E_{orb(2\beta)}$ = –49.4 (kcal/mol)
$\nu_{2\beta} = \pm 0.615$

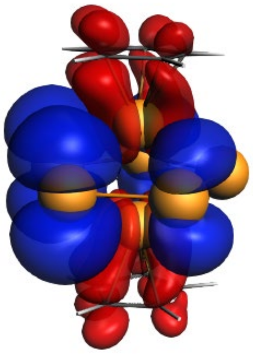

$\Delta E_{orb(2)}$ = –85.8 (kcal/mol)
$\nu_{2} = \pm 1.134$

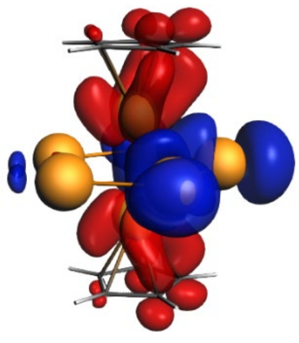

$\Delta E_{orb(3\alpha)} = -39.3$ (kcal/mol)
$\nu_{3\alpha} = \pm 0.551$

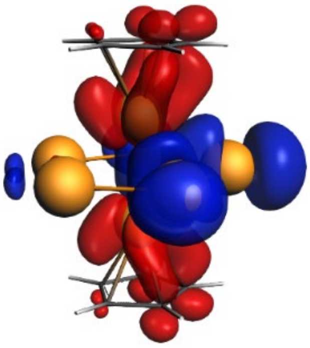

$\Delta E_{orb(3\beta)} = -41.4$ (kcal/mol)
$\nu_{3\beta} = \pm 0.570$

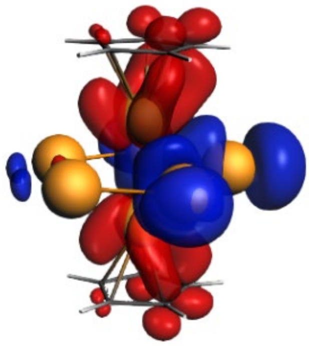

$\Delta E_{orb(3)} = -80.7$ (kcal/mol)
$\nu_{3} = \pm 1.121$

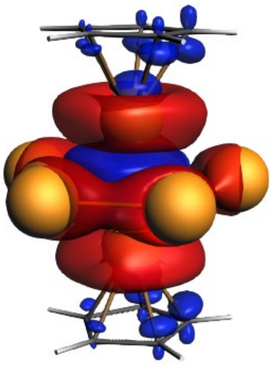

$\Delta E_{orb(4\alpha)} = -32.0$ (kcal/mol)
$\nu_{4\alpha} = \pm 0.411$

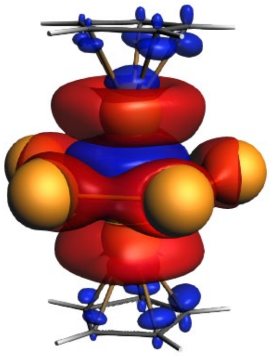

$\Delta E_{orb(4\beta)} = -33.8$ (kcal/mol)
$\nu_{4\beta} = \pm 0.427$

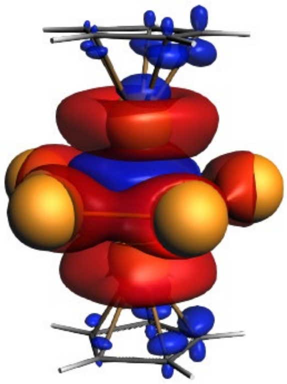

$\Delta E_{orb(4)} = -65.8$ (kcal/mol)
$\nu_{4} = \pm 0.838$

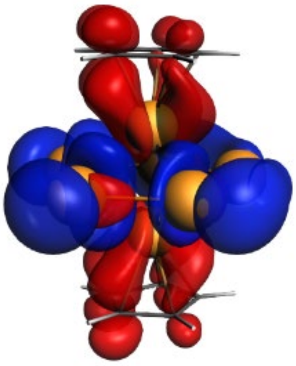

$\Delta E_{orb(5\alpha)} = -31.8$ (kcal/mol)
$\nu_5 = \pm 0.521$

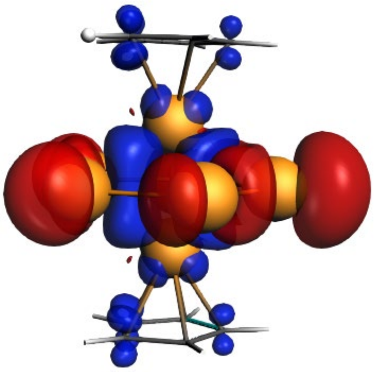

$\Delta E_{orb(6\alpha)} = -11.1$ (kcal/mol)
$\nu_{6\alpha} = \pm 0.344$

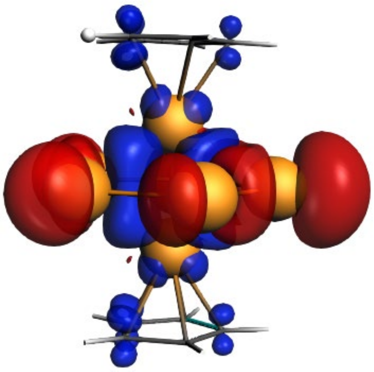

$\Delta E_{orb(6\beta)} = -10.6$ (kcal/mol)
$\nu_{6\beta} = \pm 0.332$

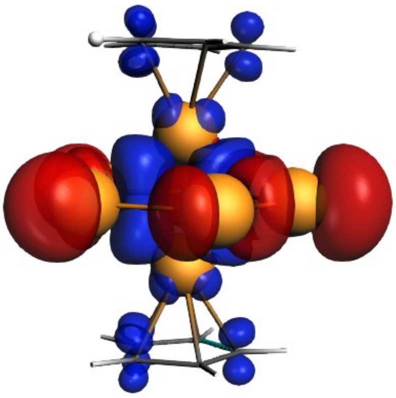

$\Delta E_{orb(6)} = -21.7$ (kcal/mol)
$\nu_6 = \pm 0.676$

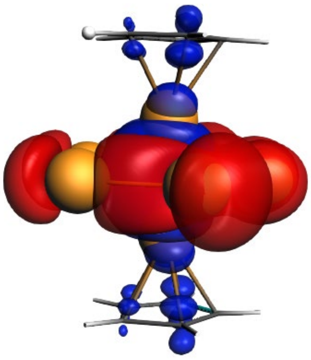

$\Delta E_{orb(7\alpha)} = -10.9$ (kcal/mol)
$\nu_{7\alpha} = \pm 0.345$

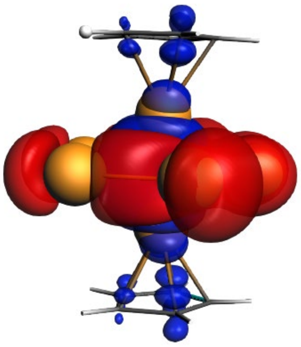

$\Delta E_{orb(7\beta)} = -10.5$ (kcal/mol)
$\nu_{7\beta} = \pm 0.335$

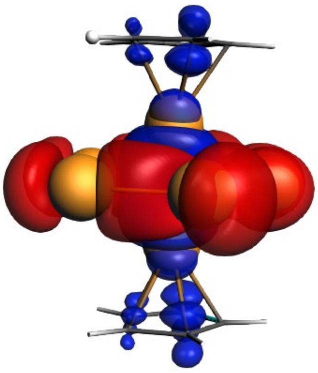

$\Delta E_{orb(7)} = -21.4$ (kcal/mol)
$\nu_{7} = \pm 0.680$

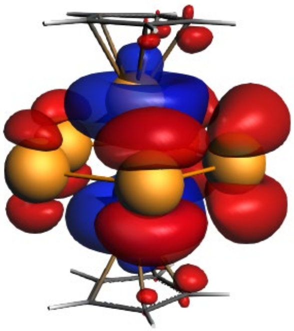

$\Delta E_{orb(8\alpha)} = -6.6$ (kcal/mol)
$\nu_{8\alpha} = \pm 0.328$

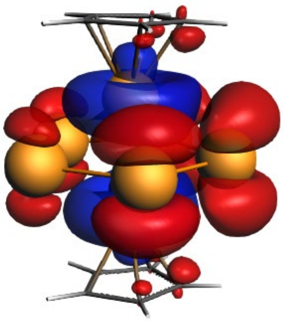

$\Delta E_{orb(8\beta)} = -5.9$ (kcal/mol)
$\nu_{8\beta} = \pm 0.303$

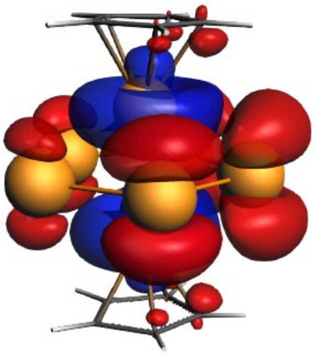

$\Delta E_{orb(8)} = -12.5$ (kcal/mol)
$\nu_{8} = \pm 0.631$

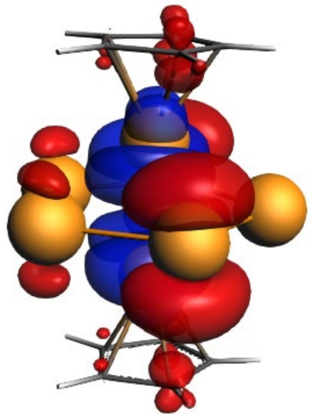

$\Delta E_{orb(9\alpha)} = -3.2$ (kcal/mol)
$\nu_{9\alpha} = \pm 0.244$

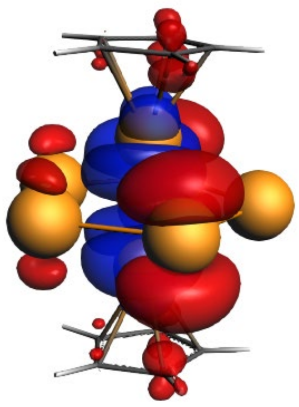

$\Delta E_{orb(9\beta)} = -3.0$ (kcal/mol)
$\nu_{9\beta} = \pm 0.220$

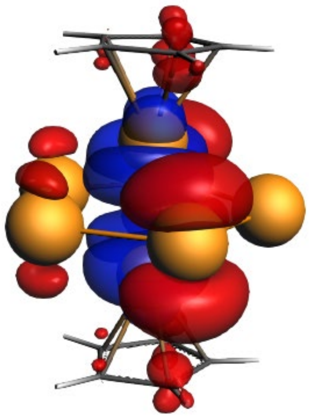

$\Delta E_{orb(9)} = -6.2$ (kcal/mol)
$\nu_{9} = \pm 0.464$

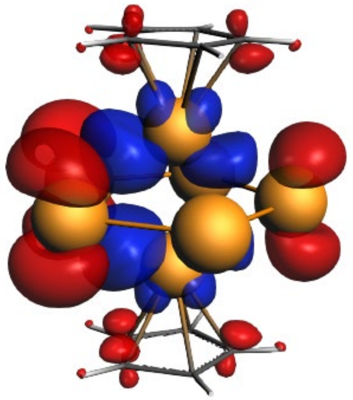

$\Delta E_{orb(10\alpha)} = -3.2$ (kcal/mol)
$\nu_{10\alpha} = \pm 0.240$

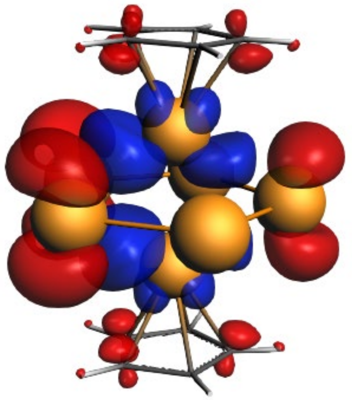

$\Delta E_{orb(10\beta)} = -3.0$ (kcal/mol)
$\nu_{10\beta} = \pm 0.215$

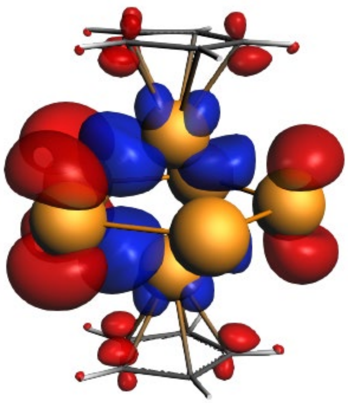

$\Delta E_{orb(10)} = –6.2$ (kcal/mol)
$\nu_{10} = \pm 0.455$

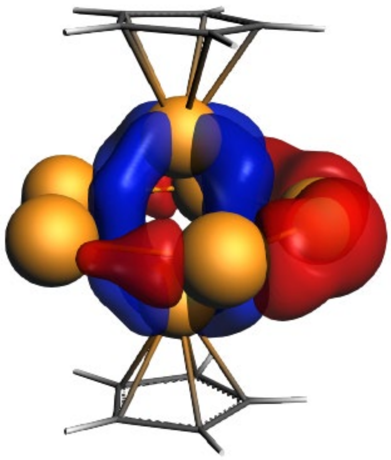

$\Delta E_{orb(11\beta)} = –2.9$ (kcal/mol)
$\nu_{11\beta} = \pm 0.196$

Supplementary Figure 17. Plot of deformation densities $\Delta\rho$ of the pairwise orbital interactions between the two fragments of Sb_5^{1-}(S) and $(VCp)_2^{1-}$(D) in $[V_2Cp_2Sb_5]^{2-}$ together with the associated interaction energies ΔE_{orb} (in kcal/mol). The eigenvalues ν are a measure for the relative amount of charge transfer. The direction of the charge flow is from red to blue.

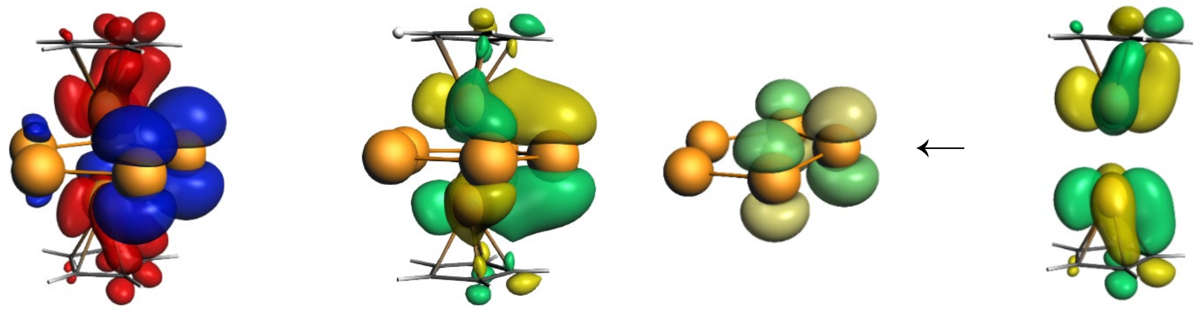

$\Delta E_{orb(1\alpha)} = -43.3$ (kcal/mol)<br>$\nu_{1\alpha} = \pm 0.539$	HOMO-7	**Sb_5^{1-}** (LUMO)<br>($\nu = 0.432$)	**$(VCp)_2^{1-}$** (HOMO-1)<br>($\nu = -0.433$)

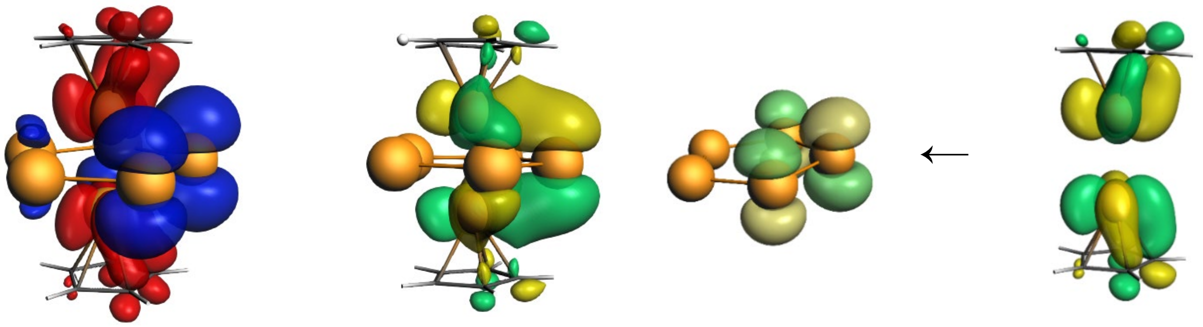

$\Delta E_{orb(1\beta)} = -46.0$ (kcal/mol)<br>$\nu_{1\beta} = \pm 0.563$	HOMO-5	**Sb_5^{1-}** (LUMO)<br>($\nu = 0.463$)	**$(VCp)_2^{1-}$** (HOMO-1)<br>($\nu = -0.463$)

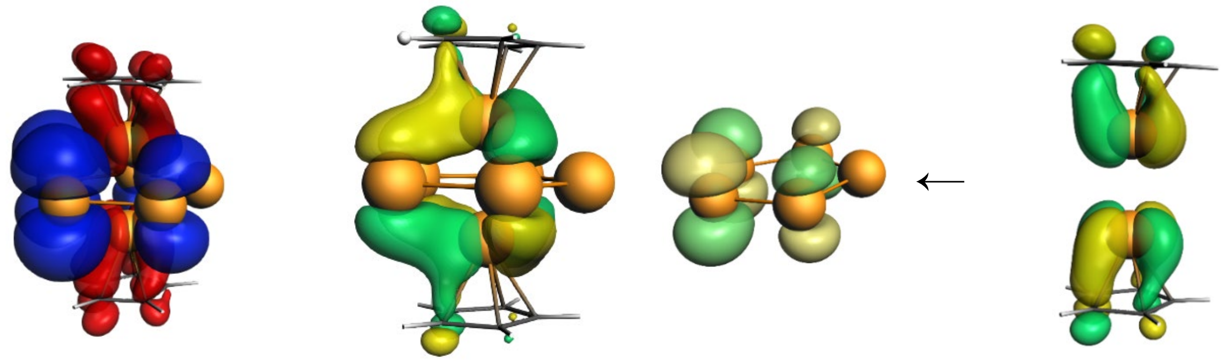

$\Delta E_{orb(2\alpha)} = -36.4$ (kcal/mol)<br>$\nu_{2\alpha} = \pm 0.509$	HOMO-6	**Sb_5^{1-}** (LUMO+1)<br>($\nu = 0.382$)	**$(VCp)_2^{1-}$** (HOMO)<br>($\nu = -0.387$)

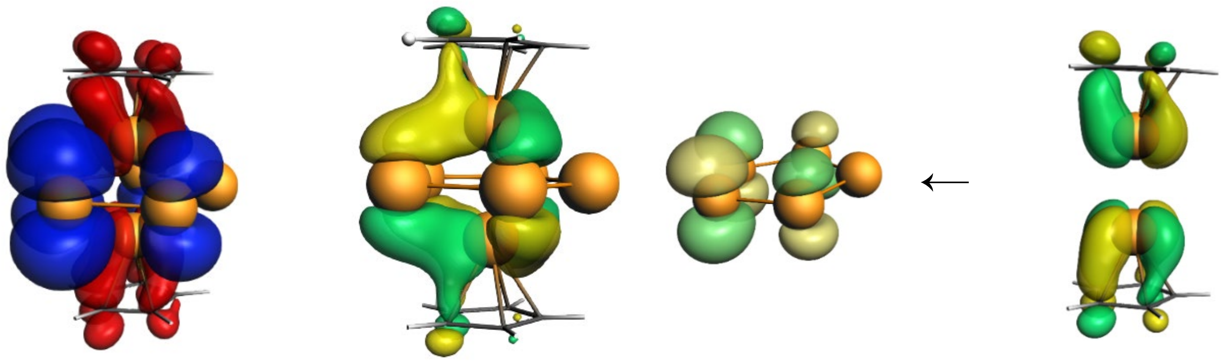

$\Delta E_{orb(2\beta)} = -49.4$ (kcal/mol)<br>$\nu_{2\beta} = \pm 0.615$	HOMO-4	**Sb_5^{1-}** (LUMO+1)<br>($\nu = 0.517$)	**$(VCp)_2^{1-}$** (HOMO)<br>($\nu = -0.516$)

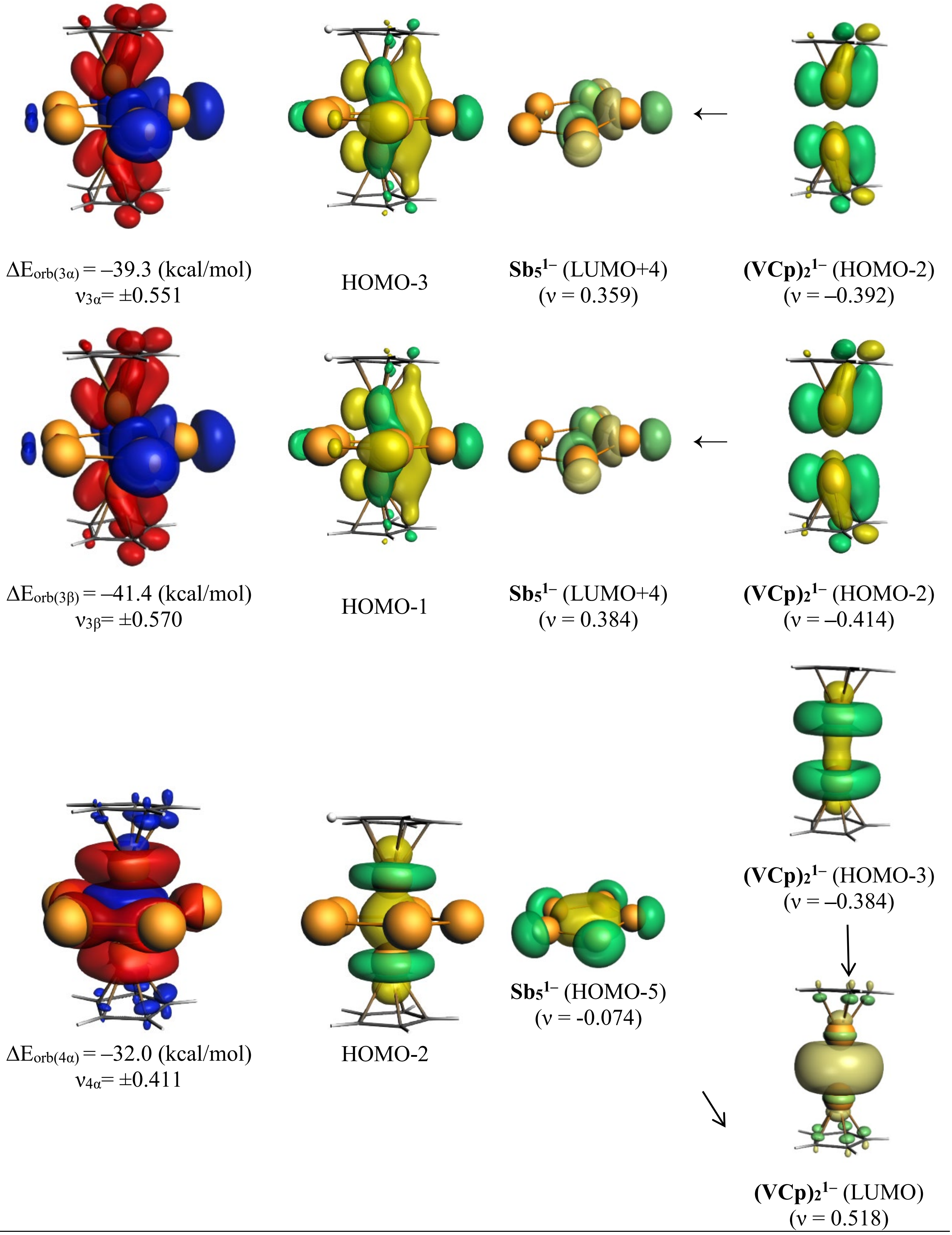
$\Delta E_{orb(3\alpha)}$ = –39.3 (kcal/mol)
$\nu_{3\alpha}$= ±0.551
HOMO-3
Sb$_5$$^{1-}$ (LUMO+4)
(ν = 0.359)
(VCp)$_2$$^{1-}$ (HOMO-2)
(ν = –0.392)
$\Delta E_{orb(3\beta)}$ = –41.4 (kcal/mol)
$\nu_{3\beta}$= ±0.570
HOMO-1
Sb$_5$$^{1-}$ (LUMO+4)
(ν = 0.384)
(VCp)$_2$$^{1-}$ (HOMO-2)
(ν = –0.414)
(VCp)$_2$$^{1-}$ (HOMO-3)
(ν = –0.384)
$\Delta E_{orb(4\alpha)}$ = –32.0 (kcal/mol)
$\nu_{4\alpha}$= ±0.411
HOMO-2
Sb$_5$$^{1-}$ (HOMO-5)
(ν = -0.074)
(VCp)$_2$$^{1-}$ (LUMO)
(ν = 0.518)

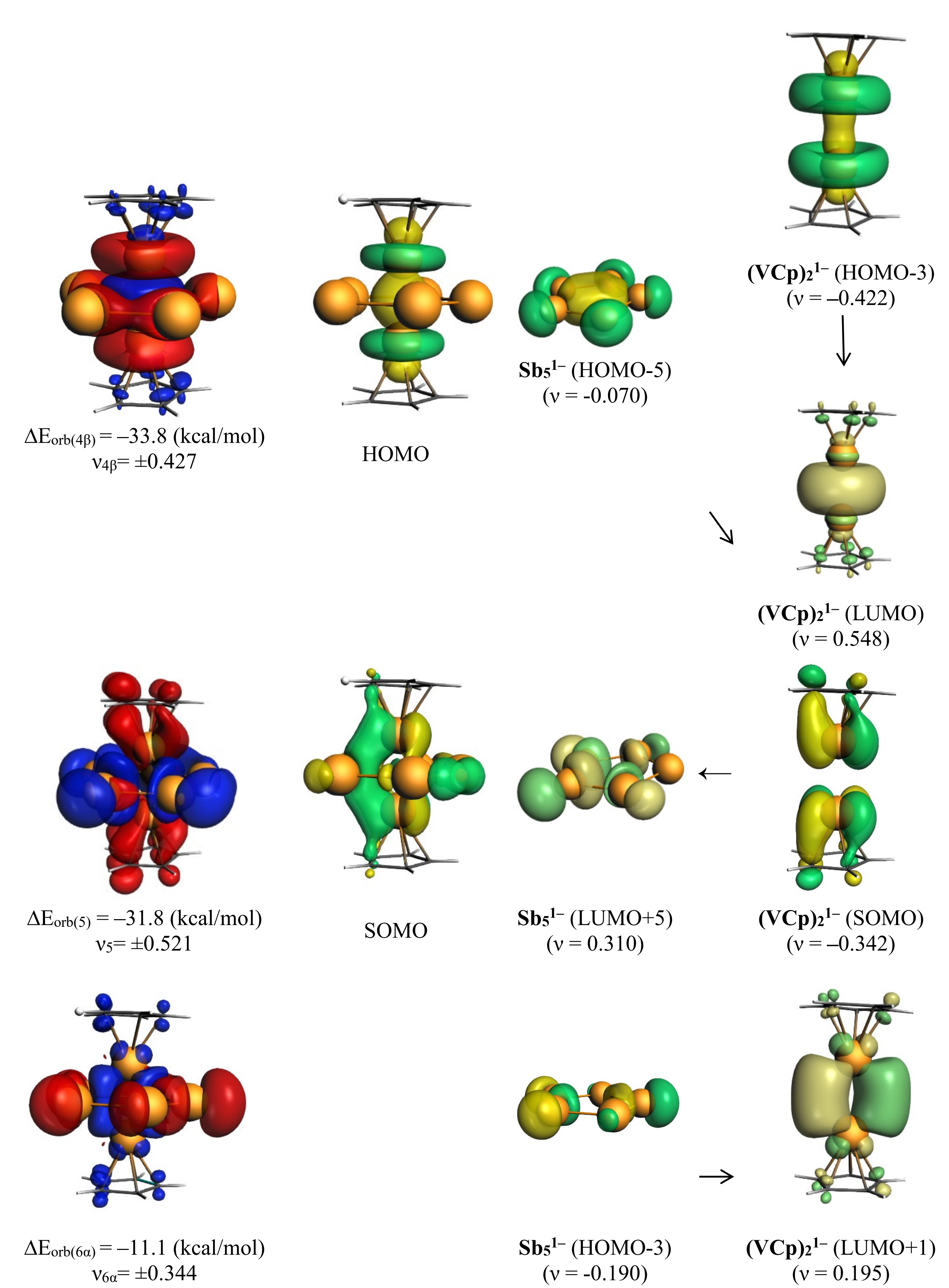
(VCp)2 1– (HOMO-3)
(ν = –0.422)
Sb5 1– (HOMO-5)
(ν = -0.070)
ΔEorb(4β) = –33.8 (kcal/mol)
ν4β= ±0.427
HOMO
(VCp)2 1– (LUMO)
(ν = 0.548)
ΔEorb(5) = –31.8 (kcal/mol)
ν5= ±0.521
SOMO
Sb5 1– (LUMO+5)
(ν = 0.310)
(VCp)2 1– (SOMO)
(ν = –0.342)
ΔEorb(6α) = –11.1 (kcal/mol)
ν6α= ±0.344
Sb5 1– (HOMO-3)
(ν = -0.190)
(VCp)2 1– (LUMO+1)
(ν = 0.195)

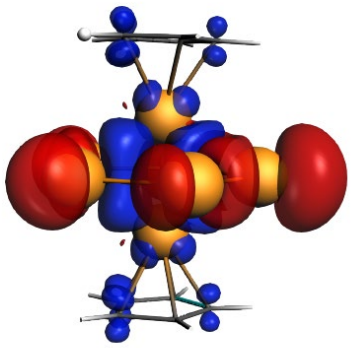

$\Delta E_{orb(6\beta)} = -10.6$ (kcal/mol)
$\nu_{6\beta} = \pm 0.332$

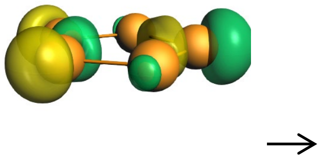

Sb_5^{1-} (HOMO-3)
(ν = -0.175)

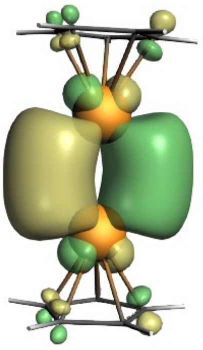

$(VCp)_2^{1-}$ (LUMO+1)
(ν = 0.182)

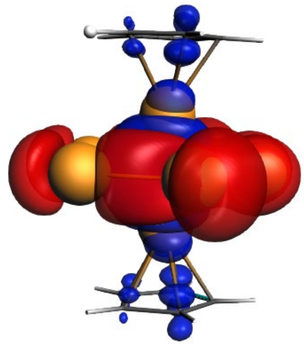

$\Delta E_{orb(7\alpha)} = -10.9$ (kcal/mol)
$\nu_{7\alpha} = \pm 0.345$

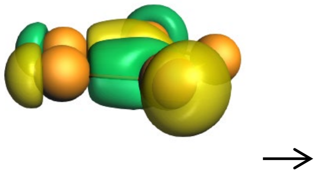

Sb_5^{1-} (HOMO-2)
(ν = -0.190)

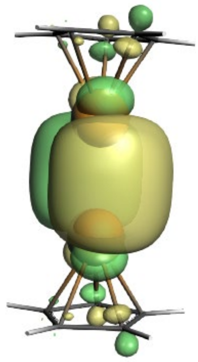

$(VCp)_2^{1-}$ (LUMO+2)
(ν = 0.197)

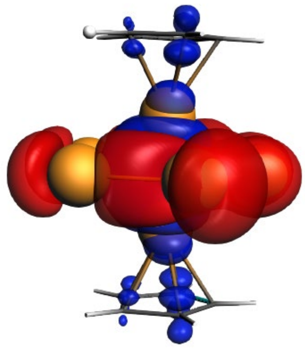

$\Delta E_{orb(7\beta)} = -10.5$ (kcal/mol)
$\nu_{7\beta} = \pm 0.335$

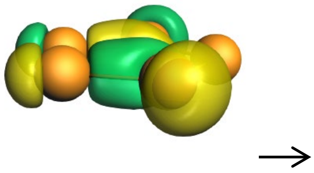

Sb_5^{1-} (HOMO-2)
(ν = -0.178)

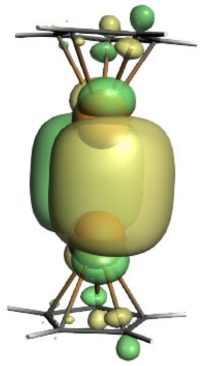

$(VCp)_2^{1-}$ (LUMO+2)
(ν = 0.185)

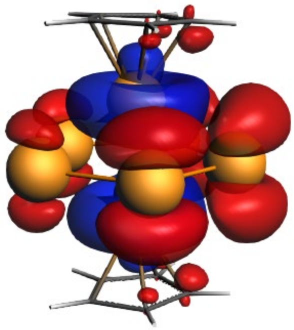

$\Delta E_{orb(8\alpha)} = -6.6$ (kcal/mol)
$\nu_{8\alpha} = \pm 0.328$

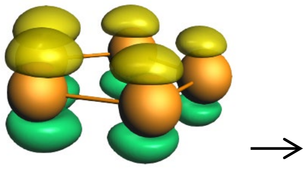

Sb_5^{1-} (HOMO-4)
(ν = -0.231)

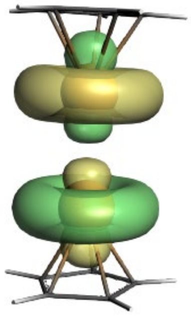

$(VCp)_2^{1-}$ (LUMO+3)
(ν = 0.246)

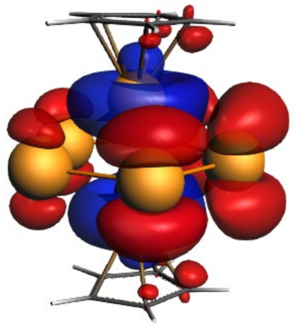	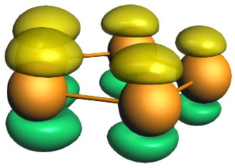 →	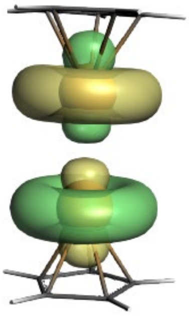
$\Delta E_{orb(8\beta)} = -5.9$ (kcal/mol)<br>$\nu_{8\beta} = \pm 0.303$	**Sb_5^{1-}** (HOMO-4)<br>(ν = -0.204)	**$(VCp)_2^{1-}$** (LUMO+3)<br>(ν = 0.220)
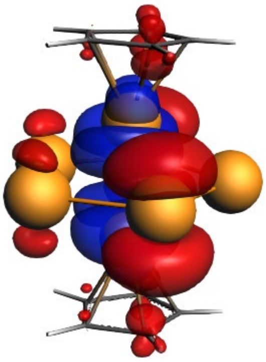	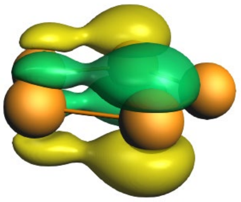 →	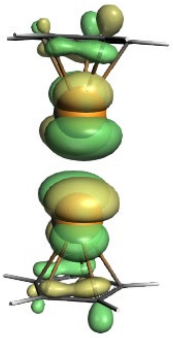
$\Delta E_{orb(9\alpha)} = -3.2$ (kcal/mol)<br>$\nu_{9\alpha} = \pm 0.244$	**Sb_5^{1-}** (HOMO-1)<br>(ν = -0.138)	**$(VCp)_2^{1-}$** (LUMO+5)<br>(ν = 0.114)
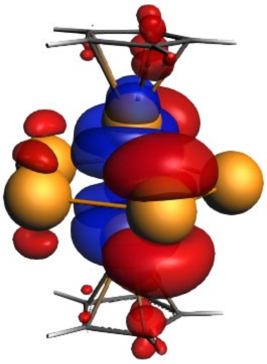	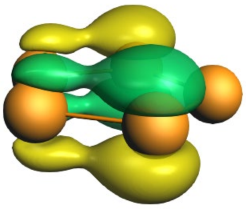 →	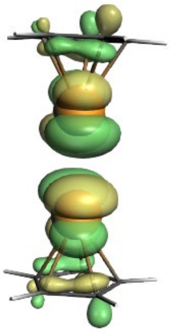
$\Delta E_{orb(9\beta)} = -3.0$ (kcal/mol)<br>$\nu_{9\beta} = \pm 0.220$	**Sb_5^{1-}** (HOMO-1)<br>(ν = -0.119)	**$(VCp)_2^{1-}$** (LUMO+5)<br>(ν = 0.098)
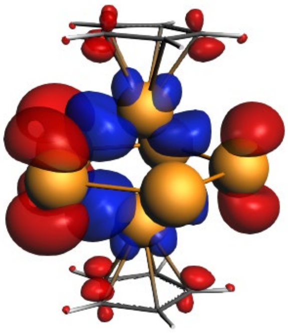	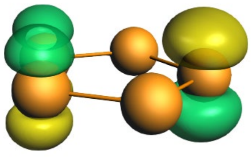 →	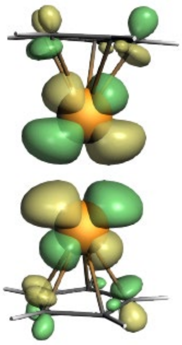
$\Delta E_{orb(10\alpha)} = -3.2$ (kcal/mol)<br>$\nu_{10\alpha} = \pm 0.240$	**Sb_5^{1-}** (HOMO)<br>(ν = -0.122)	**$(VCp)_2^{1-}$** (LUMO+4)<br>(ν = 0.107)

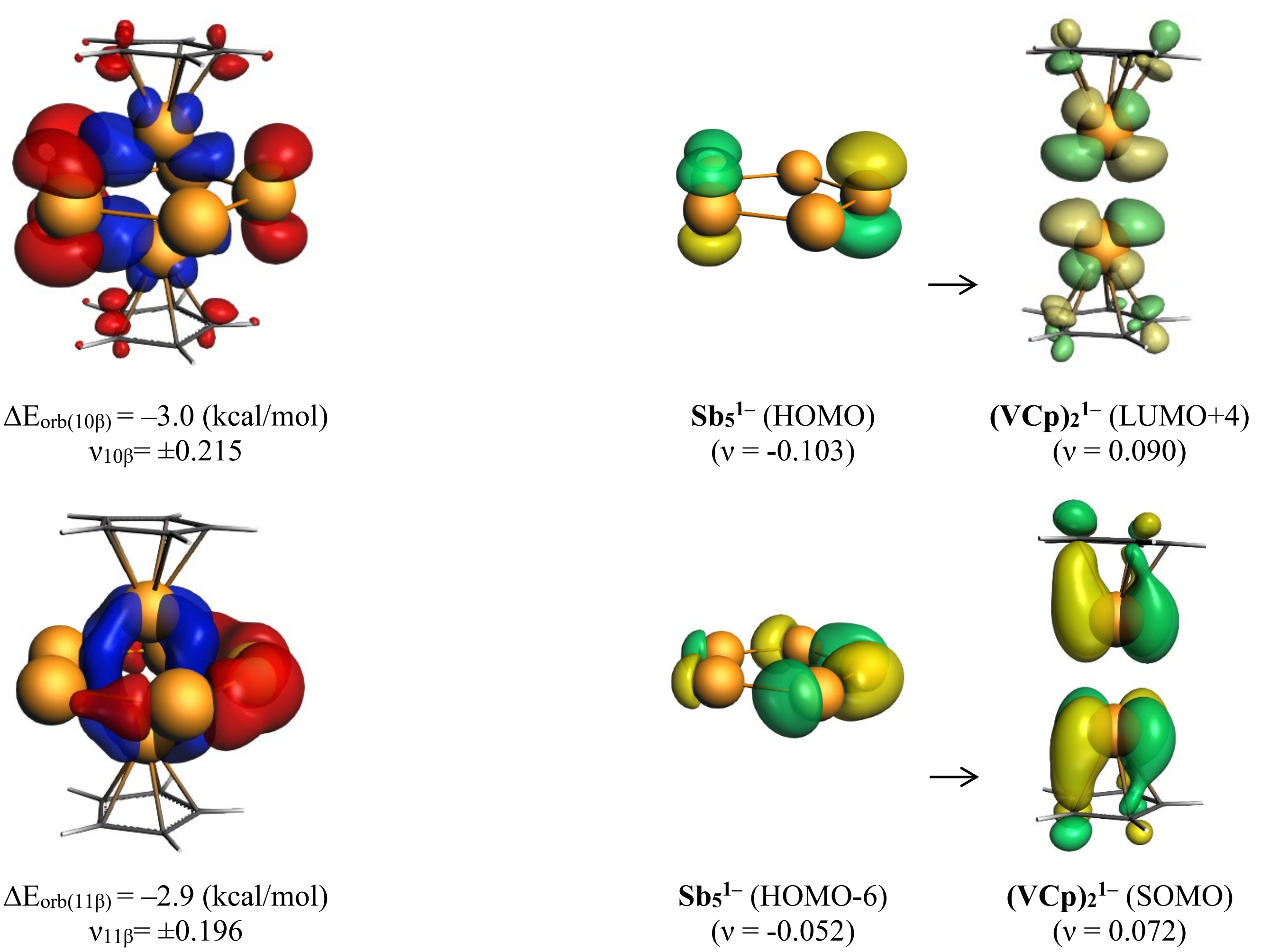

Supplementary Figure 18. Plot of deformation densities $\Delta\rho$ of the pairwise orbital interactions and the associated interaction energies (ΔE_{orb}) between fragments, as well as the shape of the most important interacting MOs of the two fragments Sb_5^{1-} (S) and $(VCp)_2^{1-}$ (D) in $[V_2Cp_2Sb_5]^{2-}$. The direction of the charge flow is red to blue.

Supplementary Table 4. EDA-NOCV results of $[Nb_2Cp_2Bi_5]^{2-}$ at the BP86-D3(BJ)/TZ2P level of theory. Fragments are given on table in singlet (S) or Doublet (D) electronic states. Energy values are given in kcal/mol.

Fragments	**[cyclo-Bi₅]⁻** (S)+ **(NbCp)₂¹⁻** (D)	**[cyclo-Bi₅]⁺** (S)+ **(NbCp)₂³⁻** (D)	**[cyclo-Bi₅]³⁻** (D)+ **(NbCp)₂¹⁺** (S)
ΔE_{int}	–283.4	–760.0	–711.3
ΔE_{Pauli}	1020.1	1167.4	768.7
ΔE_{elstat}[a]	–775.9 (59.5%)	–1182.9 (61.3%)	–876.5 (59.2%)
ΔE_{disp}[a]	–50.0 (3.8%)	–50.0 (2.6%)	–50.0 (3.4%)
ΔE_{orb}[a]	–477.6 (36.7%)	–694.5 (36.1%)	–553.5 (37.4%)
$\Delta E_{orb(1)}$[b]	–90.2 (18.9%)		
$\Delta E_{orb(2)}$[b]	–88.0 (18.4%)		
$\Delta E_{orb(3)}$[b]	–74.8 (15.7%)		
$\Delta E_{orb(4)}$[b]	–70.2 (14.7%)		
$\Delta E_{orb(5)}$[b]	–59.9 (12.5%)		
$\Delta E_{orb(6)}$[b]	–22.5 (4.7%)		
$\Delta E_{orb(7)}$[b]	–19.1 (4.0%)		
$\Delta E_{orb(8)}$[b]	–18.4 (3.9%)		
$\Delta E_{orb(9)}$[b]	–6.6 (1.4%)		
$\Delta E_{orb(10)}$[b]	–6.3 (1.3%)		
$\Delta E_{orb(11)}$[b]	–5.0 (1.0%)		
$\Delta E_{orb(rest)}$[b]	–16.6 (3.5%)		

[a]The values in parentheses give the percentage contribution to the total attractive interactions $\Delta E_{elstat} + \Delta E_{orb} + \Delta E_{disp}$.

[b]The values in parentheses give the percentage contribution to the total orbital interactions ΔE_{orb}.

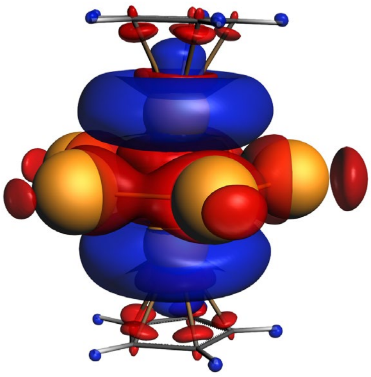

$\Delta E_{orb(1\alpha)}$ = –45.7 (kcal/mol)
$\nu_{1\alpha}$= ±0.590

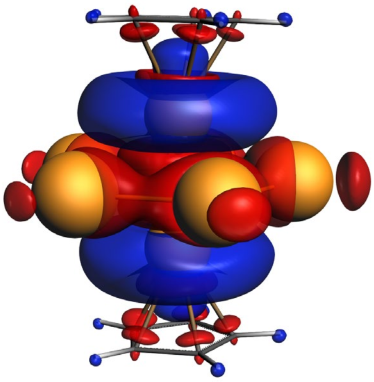

$\Delta E_{orb(1\beta)}$ = –44.5 (kcal/mol)
$\nu_{1\beta}$= ±0.580

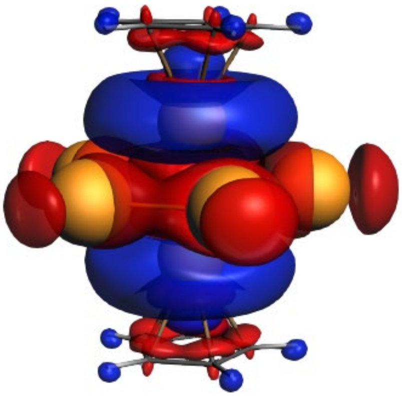

$\Delta E_{orb(1)}$ = –90.2 (kcal/mol)
ν_{1} = ±1.170

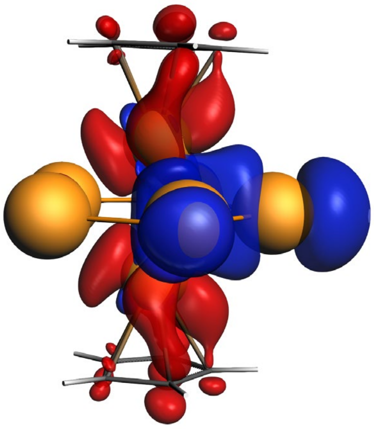

$\Delta E_{orb(2\alpha)}$ = –43.6 (kcal/mol)
$\nu_{2\alpha}$= ±0.675

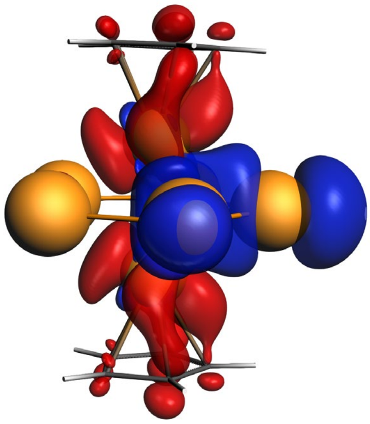

$\Delta E_{orb(2\beta)}$ = –44.4 (kcal/mol)
$\nu_{2\beta}$= ±0.680

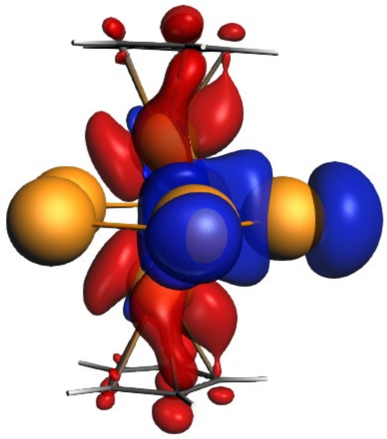

$\Delta E_{orb(2)}$ = –88.0 (kcal/mol)
ν_2 = ±1.355

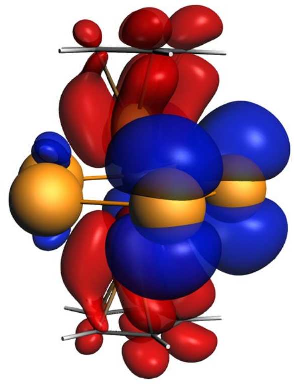

$\Delta E_{orb(3\alpha)}$ = –36.9 (kcal/mol)
$\nu_{3\alpha}$= ±0.540

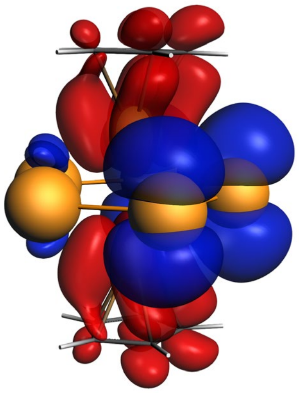

$\Delta E_{orb(3\beta)}$ = –37.9 (kcal/mol)
$\nu_{3\beta}$= ±0.548

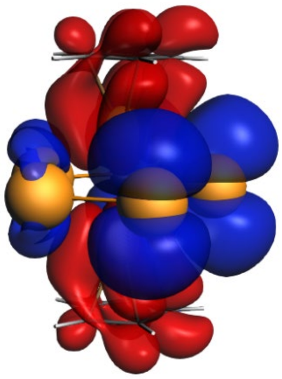

$\Delta E_{orb(3)}$= –74.8 (kcal/mol)
ν_3 = ±1.088

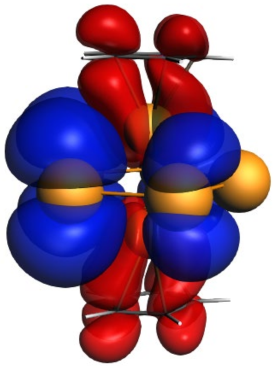

$\Delta E_{orb(4\alpha)} = -32.8$ (kcal/mol)
$\nu_{4\alpha} = \pm 0.528$

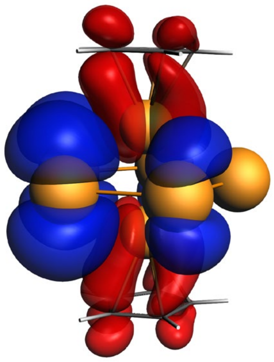

$\Delta E_{orb(4\beta)} = -37.4$ (kcal/mol)
$\nu_{4\beta} = \pm 0.575$

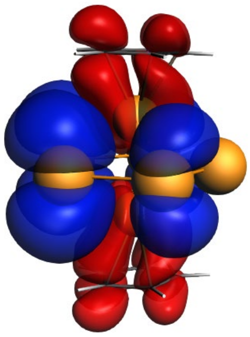

$\Delta E_{orb(4)} = -70.2$ (kcal/mol)
$\nu_{4} = \pm 1.103$

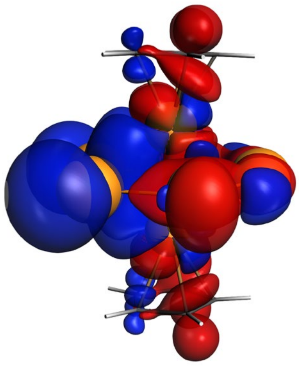

$\Delta E_{orb(5)} = -59.9$ (kcal/mol)
$\nu_{5} = \pm 0.804$

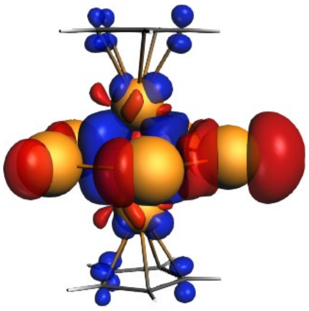

$\Delta E_{orb(6\alpha)} = -11.4$ (kcal/mol)
$\nu_{6\alpha} = \pm 0.341$

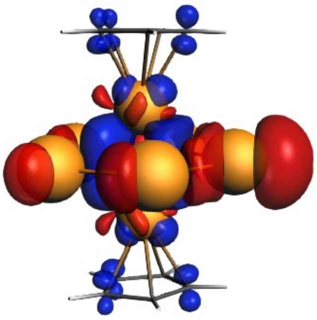

$\Delta E_{orb(6\beta)} = -11.1$ (kcal/mol)
$\nu_{6\beta} = \pm 0.338$

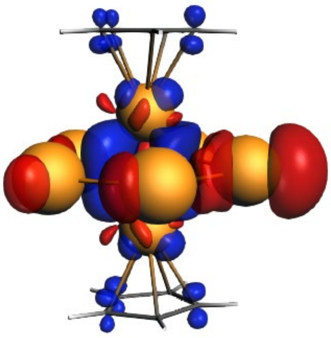

$\Delta E_{orb(6)}$= –22.5 (kcal/mol)
ν_6= ±0.663

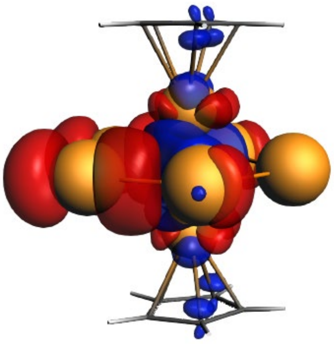

$\Delta E_{orb(7\alpha)}$ = –6.7 (kcal/mol)
$\nu_{7\alpha}$= ±0.265

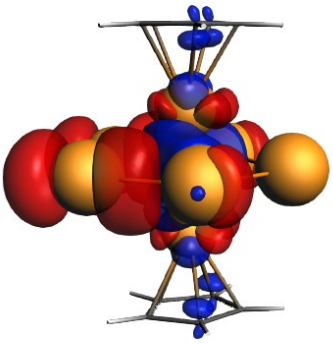

$\Delta E_{orb(7\beta)}$ = –12.4 (kcal/mol)
$\nu_{7\beta}$= ±0.367

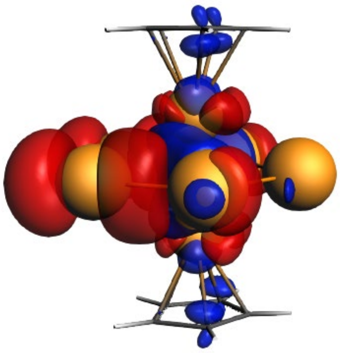

$\Delta E_{orb(7)}$= –19.1 (kcal/mol)
ν_7= ±0.632

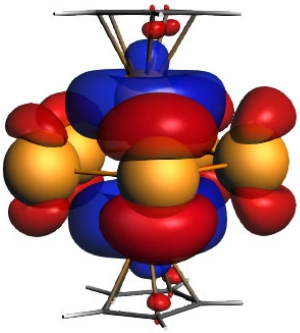

$\Delta E_{orb(8\alpha)}$ = –9.6 (kcal/mol)
$\nu_{8\alpha}$= ±0.322

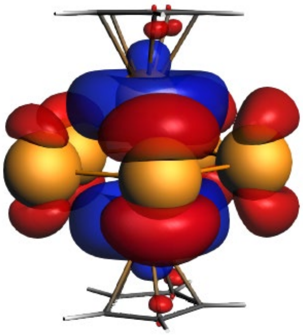

$\Delta E_{orb(8\beta)}$ = –8.8 (kcal/mol)
$\nu_{8\beta}$= ±0.307

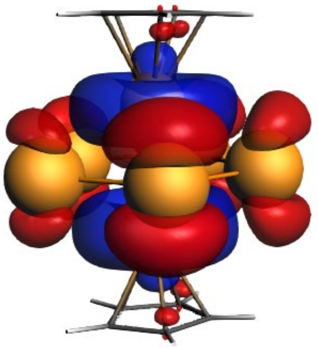

$\Delta E_{orb(8)}$= –18.4 (kcal/mol)
ν_8= ±0.629

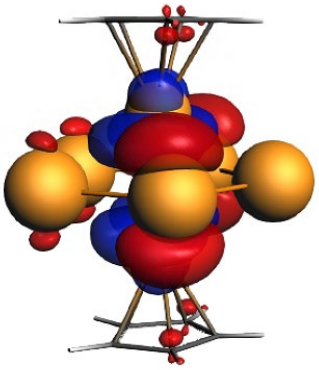

$\Delta E_{orb(9\alpha)}$ = –3.5 (kcal/mol)
$\nu_{9\alpha}$= ±0.183

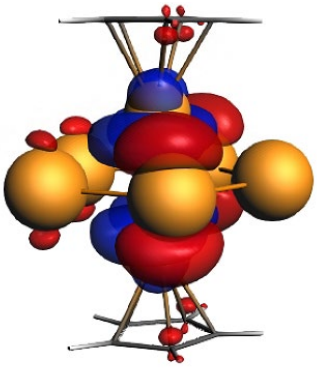

$\Delta E_{orb(9\beta)}$ = –3.1 (kcal/mol)
$\nu_{9\beta}$= ±0.174

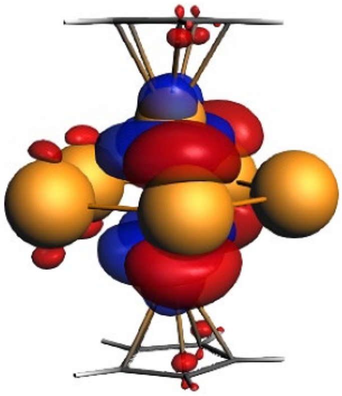

$\Delta E_{orb(9)}$= –6.6 (kcal/mol)
ν_9= ±0.357

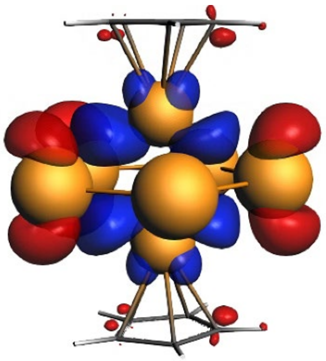

$\Delta E_{orb(10\alpha)}$ = –3.3 (kcal/mol)
$\nu_{10\alpha}$= ±0.182

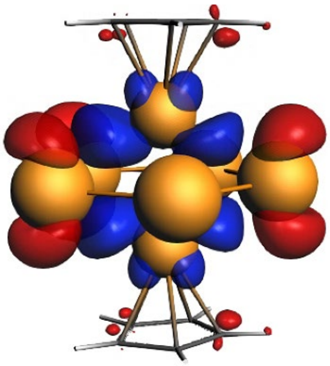

$\Delta E_{orb(10\beta)}$ = –3.0 (kcal/mol)
$\nu_{10\beta}$= ±0.172

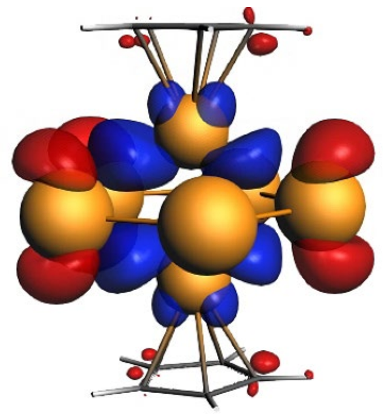

$\Delta E_{orb(10)}$= –6.3 (kcal/mol)
ν_{10}= ±0.354

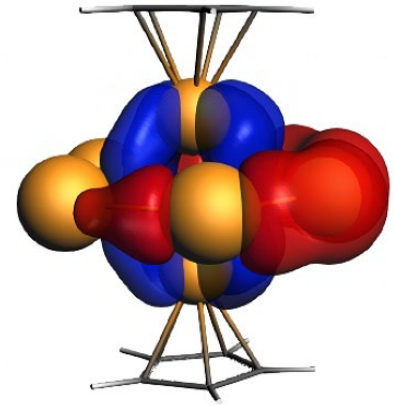

$\Delta E_{orb(11\beta)}$ = –5.0 (kcal/mol)
$\nu_{11\beta}$= ±0.269

Supplementary Figure 19. Plot of deformation densities $\Delta\rho$ of the pairwise orbital interactions between the two fragments of Bi_5^{1-}(S) and $(NbCp)_2^{1-}$(D) in $[Nb_2Cp_2Sb_5]^{2-}$ together with the associated interaction energies ΔE_{orb} (in kcal/mol). The eigenvalues ν are a measure for the relative amount of charge transfer. The direction of the charge flow is from red to blue.

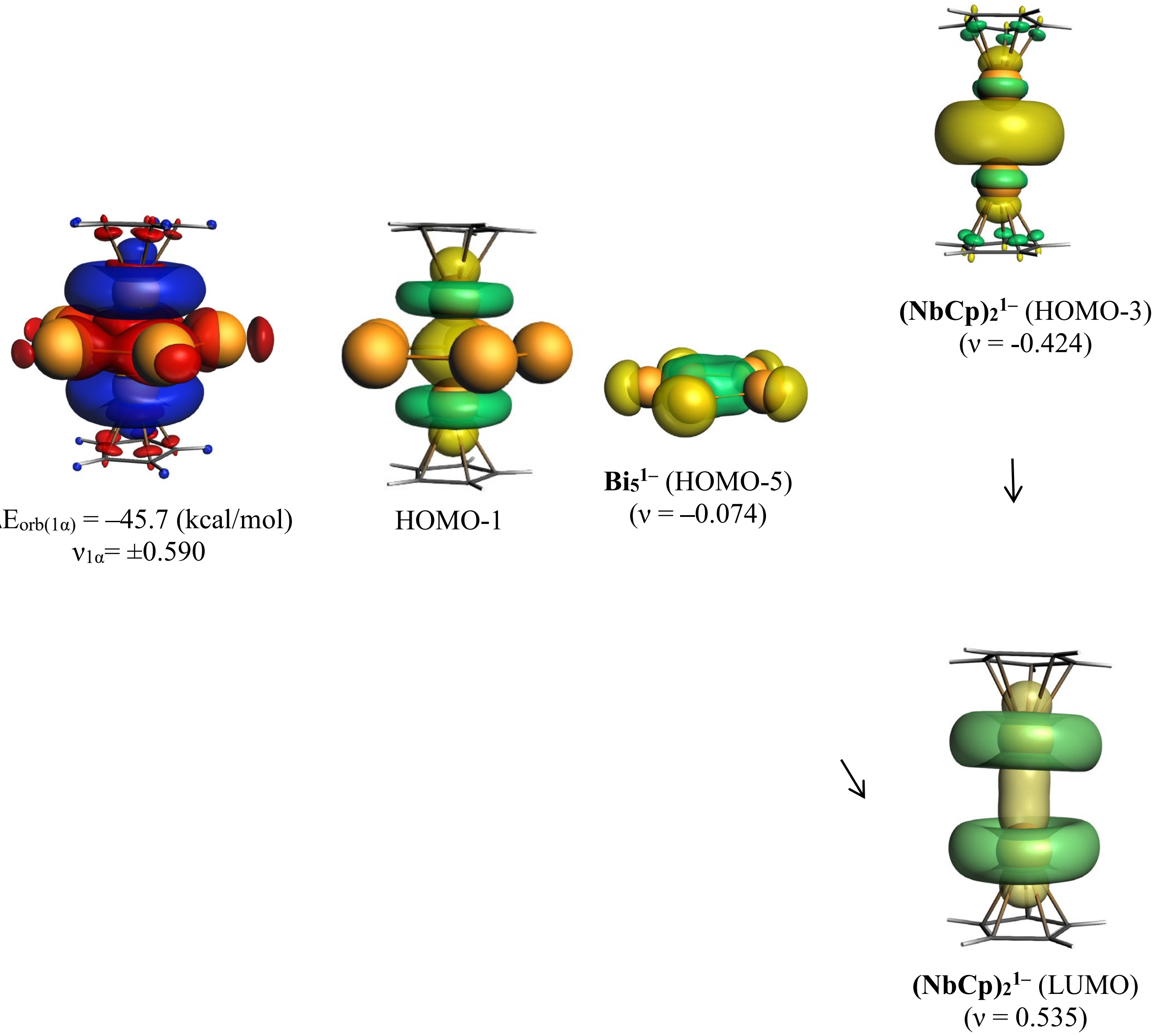
(NbCp)$_2^{1-}$ (HOMO-3)
(ν = -0.424)
$\Delta E_{orb(1\alpha)}$ = –45.7 (kcal/mol)
$\nu_{1\alpha}$= ±0.590
HOMO-1
Bi$_5^{1-}$ (HOMO-5)
(ν = –0.074)
(NbCp)$_2^{1-}$ (LUMO)
(ν = 0.535)

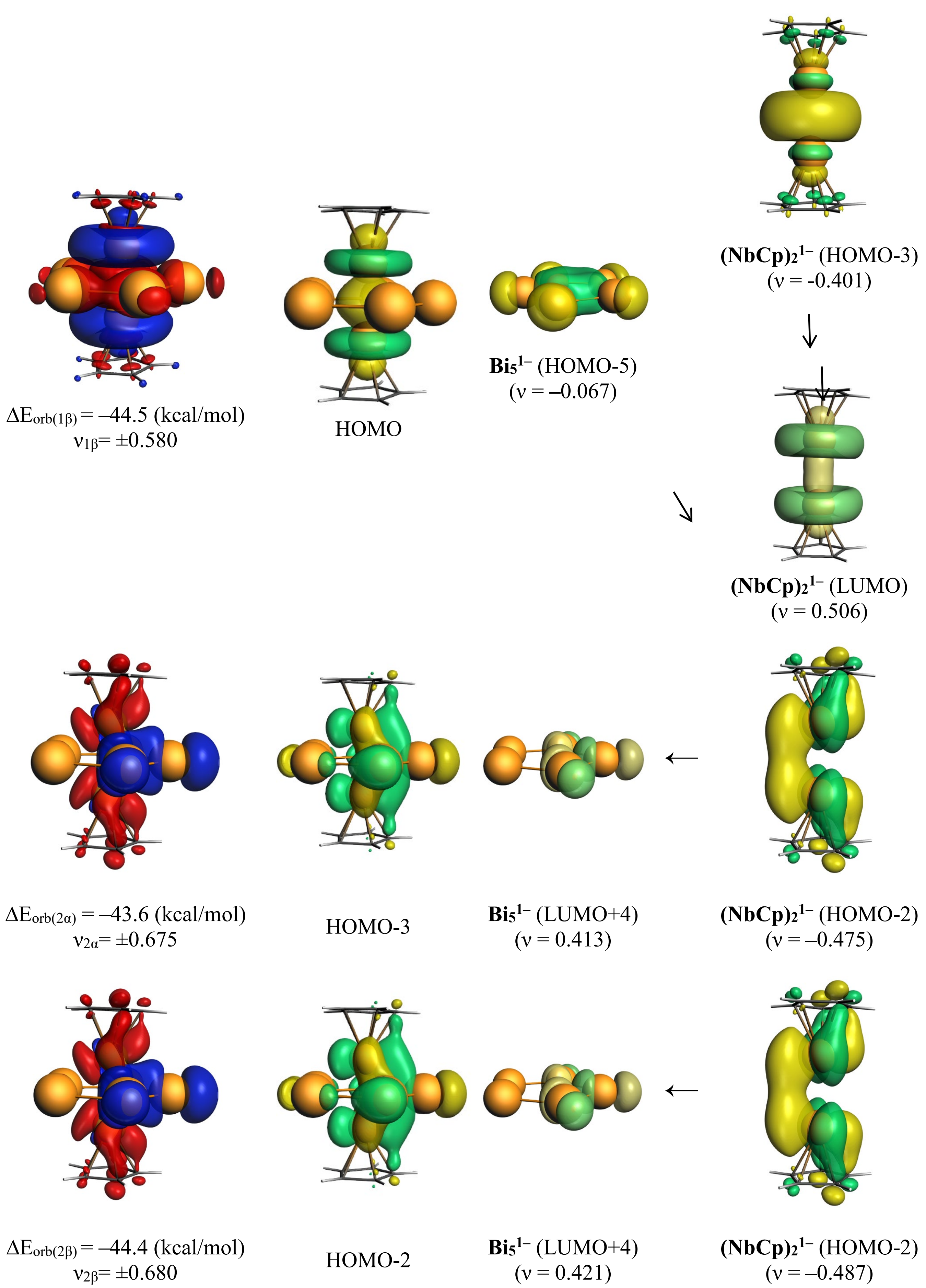

(NbCp)2 1– (HOMO-3)
(ν = -0.401)
ΔEorb(1β) = –44.5 (kcal/mol)
ν1β= ±0.580
HOMO
Bi5 1– (HOMO-5)
(ν = –0.067)
(NbCp)2 1– (LUMO)
(ν = 0.506)
ΔEorb(2α) = –43.6 (kcal/mol)
ν2α= ±0.675
HOMO-3
Bi5 1– (LUMO+4)
(ν = 0.413)
(NbCp)2 1– (HOMO-2)
(ν = –0.475)
ΔEorb(2β) = –44.4 (kcal/mol)
ν2β= ±0.680
HOMO-2
Bi5 1– (LUMO+4)
(ν = 0.421)
(NbCp)2 1– (HOMO-2)
(ν = –0.487)

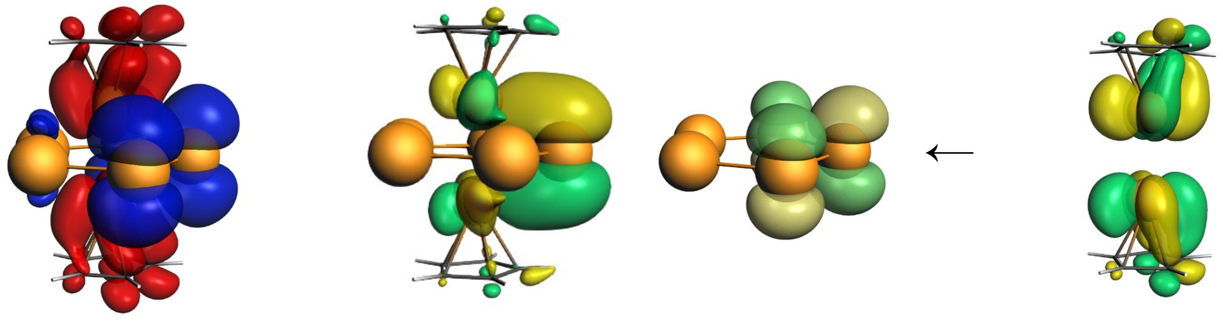

$\Delta E_{orb(3\alpha)}$ = –36.9 (kcal/mol)<br>$\nu_{3\alpha}$= ±0.540	HOMO-7	**Bi$_5$**$^{1-}$ (LUMO)<br>(ν = 0.420)	**(NbCp)$_2$**$^{1-}$ (HOMO-1)<br>(ν = –0.435)

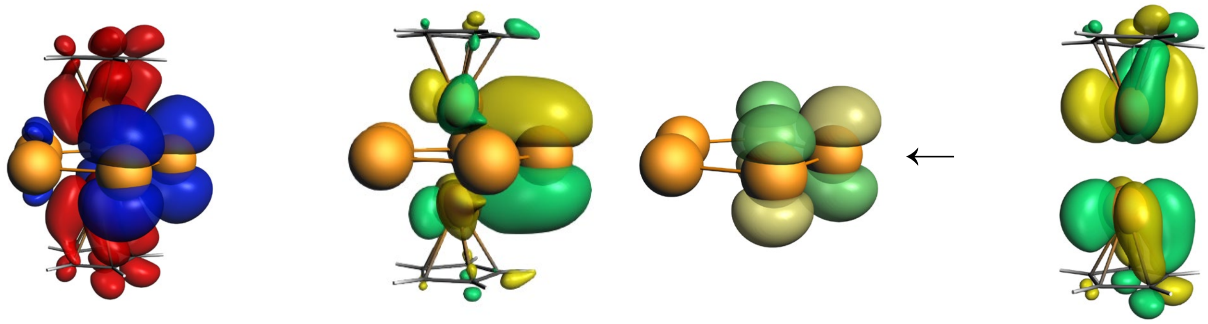

$\Delta E_{orb(3\beta)}$ = –37.9 (kcal/mol)<br>$\nu_{3\beta}$= ±0.548	HOMO-6	**Bi$_5$**$^{1-}$ (LUMO)<br>(ν = 0.431)	**(NbCp)$_2$**$^{1-}$ (HOMO-1)<br>(ν = –0.447)

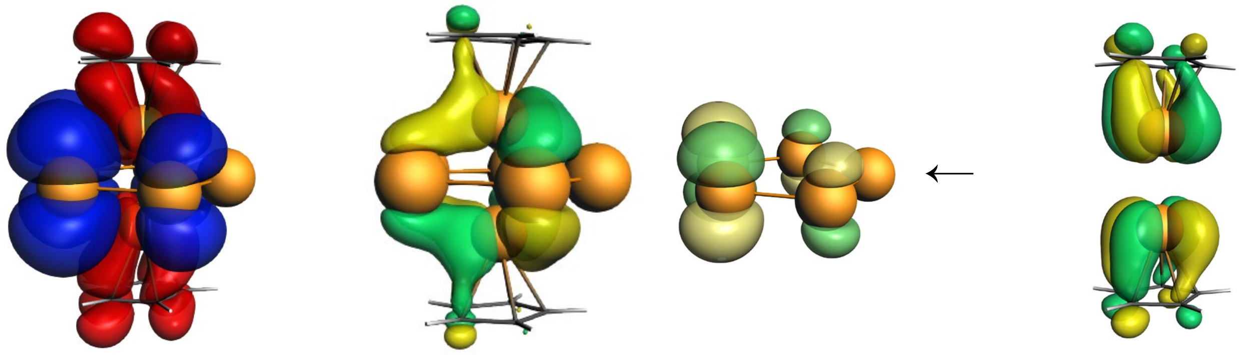

$\Delta E_{orb(4\alpha)}$ = –32.8 (kcal/mol)<br>$\nu_{4\alpha}$= ±0.528	HOMO-5	**Bi$_5$**$^{1-}$ (LUMO+1)<br>(ν = 0.396)	**(NbCp)$_2$**$^{1-}$ (HOMO)<br>(ν = –0.412)

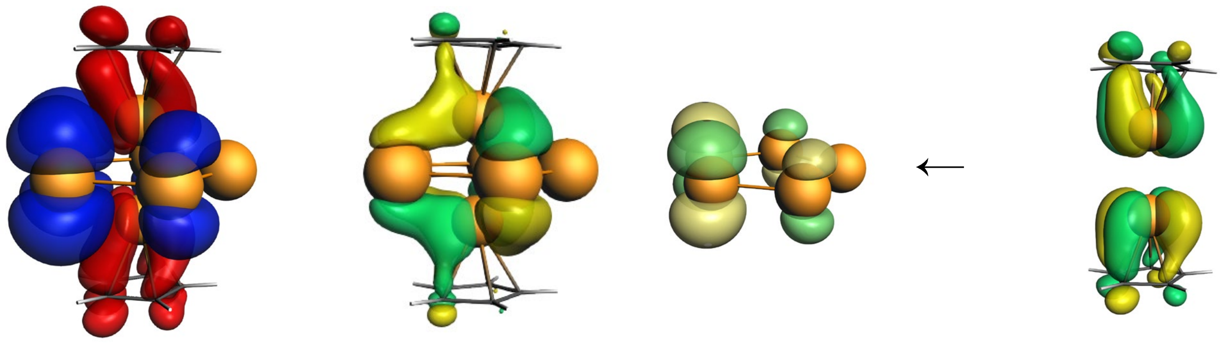

$\Delta E_{orb(4\beta)}$ = –37.4 (kcal/mol)<br>$\nu_{4\beta}$= ±0.575	HOMO-4	**Bi$_5$**$^{1-}$ (LUMO+1)<br>(ν = 0.455)	**(NbCp)$_2$**$^{1-}$ (HOMO)<br>(ν = –0.470)

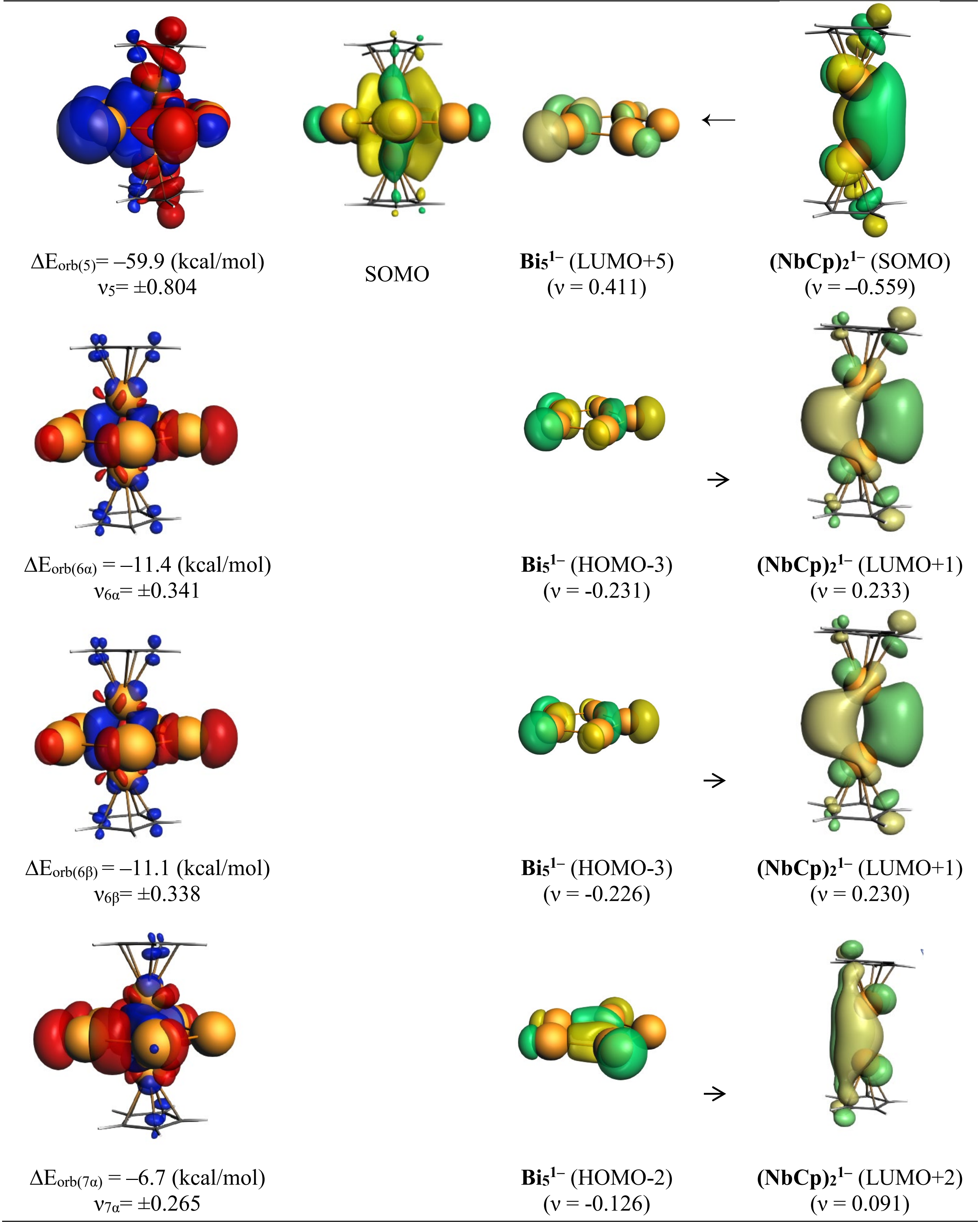

$\Delta E_{orb(5)}$ = –59.9 (kcal/mol)
ν_5 = ±0.804
SOMO
Bi_5^{1-} (LUMO+5)
(ν = 0.411)
←
$(NbCp)_2^{1-}$ (SOMO)
(ν = –0.559)
$\Delta E_{orb(6\alpha)}$ = –11.4 (kcal/mol)
$\nu_{6\alpha}$ = ±0.341
Bi_5^{1-} (HOMO-3)
(ν = -0.231)
→
$(NbCp)_2^{1-}$ (LUMO+1)
(ν = 0.233)
$\Delta E_{orb(6\beta)}$ = –11.1 (kcal/mol)
$\nu_{6\beta}$ = ±0.338
Bi_5^{1-} (HOMO-3)
(ν = -0.226)
→
$(NbCp)_2^{1-}$ (LUMO+1)
(ν = 0.230)
$\Delta E_{orb(7\alpha)}$ = –6.7 (kcal/mol)
$\nu_{7\alpha}$ = ±0.265
Bi_5^{1-} (HOMO-2)
(ν = -0.126)
→
$(NbCp)_2^{1-}$ (LUMO+2)
(ν = 0.091)

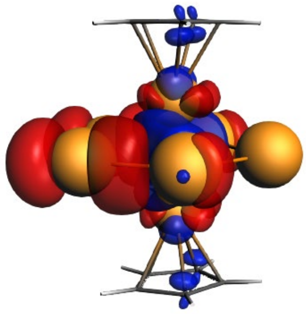	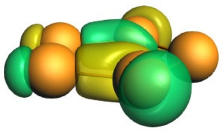	→	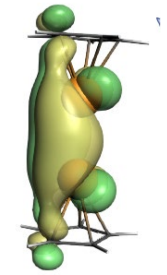
$\Delta E_{orb(7\beta)}$ = –12.4 (kcal/mol)<br>$\nu_{7\beta}$= ±0.367	**$Bi_5$$^{1-}$** (HOMO-2)<br>(ν = -0.287)		**$(NbCp)_2$$^{1-}$** (LUMO+2)<br>(ν = 0.162)
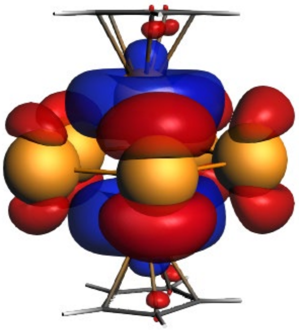	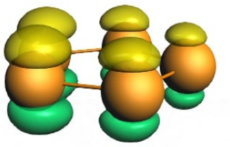	→	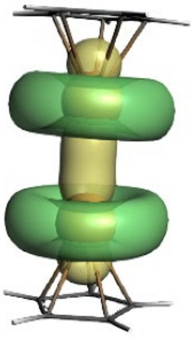
$\Delta E_{orb(8\alpha)}$ = –9.6 (kcal/mol)<br>$\nu_{8\alpha}$= ±0.322	**$Bi_5$$^{1-}$** (HOMO-4)<br>(ν = -0.247)		**$(NbCp)_2$$^{1-}$** (LUMO)<br>(ν = 0.275)
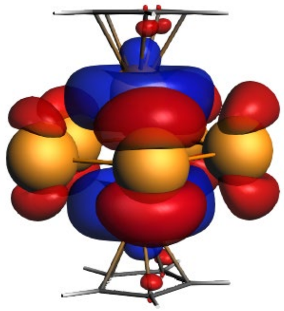	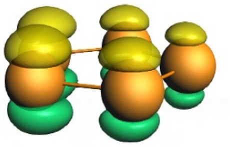	→	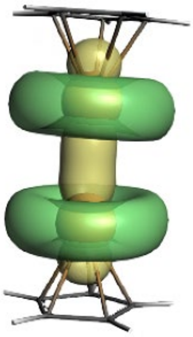
$\Delta E_{orb(8\beta)}$ = –8.8 (kcal/mol)<br>$\nu_{8\beta}$= ±0.307	**$Bi_5$$^{1-}$** (HOMO-4)<br>(ν = -0.228)		**$(NbCp)_2$$^{1-}$** (LUMO)<br>(ν = 0.258)
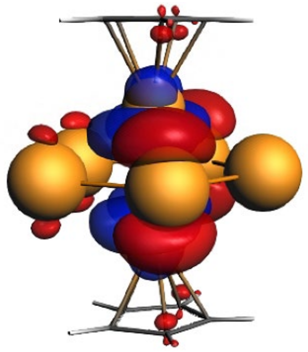	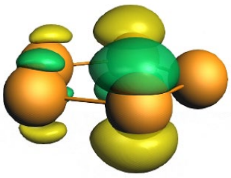	→	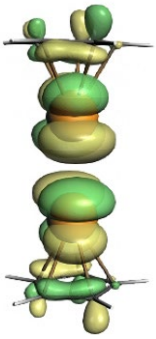
$\Delta E_{orb(9\alpha)}$ = –3.5 (kcal/mol)<br>$\nu_{9\alpha}$= ±0.183	**$Bi_5$$^{1-}$** (HOMO-1)<br>(ν = -0.139)		**$(NbCp)_2$$^{1-}$** (LUMO+6)<br>(ν = 0.077)

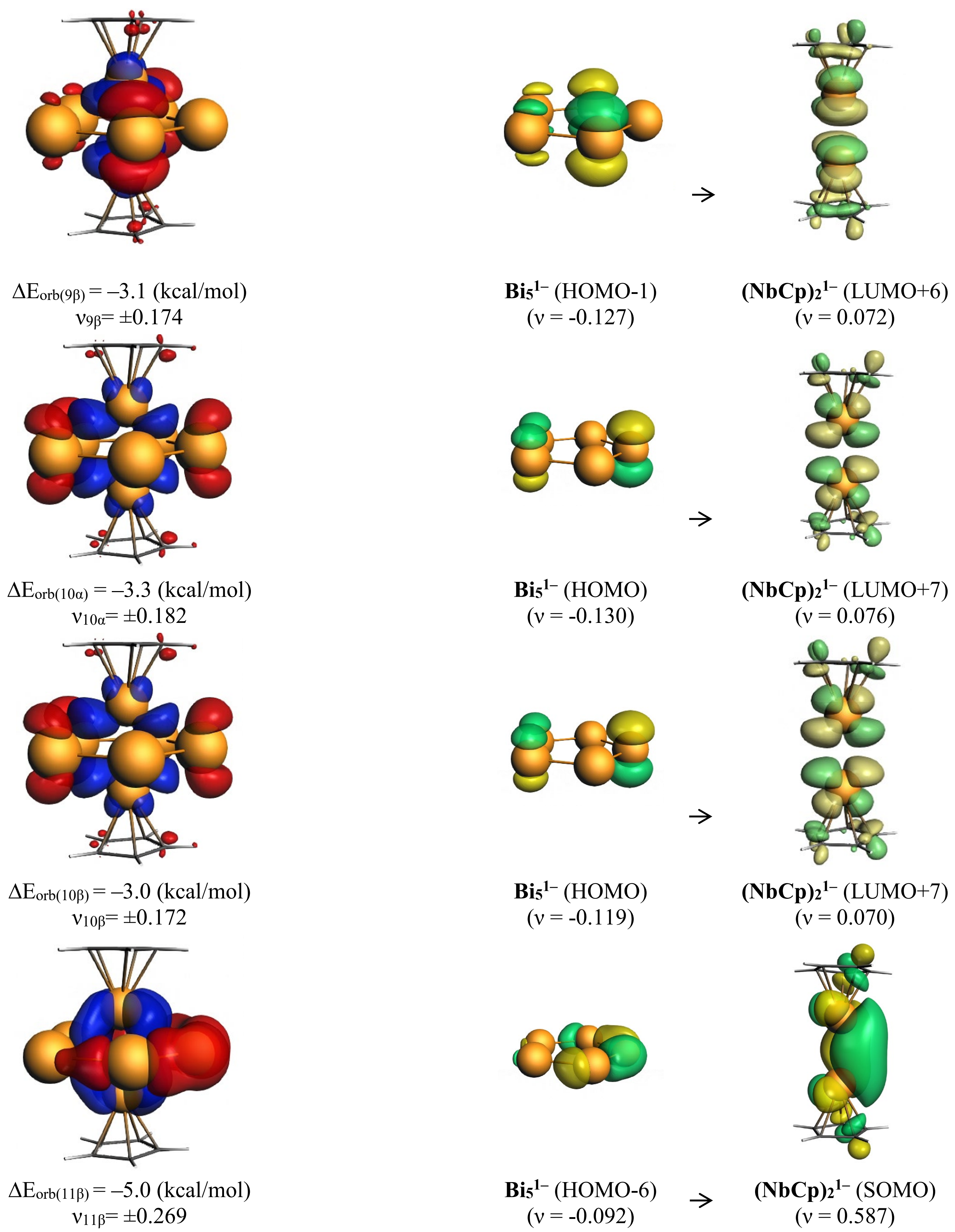

Supplementary Figure 20. Plot of deformation densities $\Delta\rho$ of the pairwise orbital interactions and the associated interaction energies (ΔE_{orb}) between fragments, as well as the shape of the most important interacting MOs of the two fragments Bi_5^{1-}(S) and $(NbCp)_2^{1-}$(D) in $[Nb_2Cp_2Bi_5]^{2-}$. The direction of the charge flow is red to blue.

Supplementary Table 5. NICS(0) and NICS(1) above and below the ring of $[V_2Cp_2Sb_5]^{2-}$ or $[Nb_2Cp_2Bi_5]^{2-}$ at the BP86/def2-TZVPP level.

Model	NICS	NICS(0)	$NICS(1)_{ZZ}$	$NICS(-1)_{ZZ}$
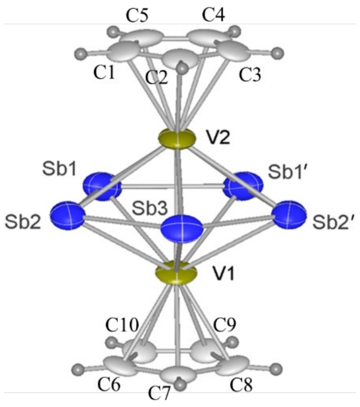	C1-C5	-14.5	-14.3	315.7
	Sb1-Sb5	18.1	1714.2	1714.2
	C6-C10	-14.5	315.7	-14.3

Model	NICS	NICS(0)	$NICS(1)_{ZZ}$	$NICS(-1)_{ZZ}$
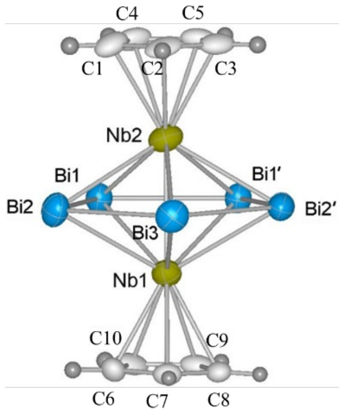	C1-C5	-14.9	-18.9	104.2
	Bi1-Bi5	12.3	461.5	461.5
	C6-C10	-14.9	104.2	-18.9

Supplementary Table 6. Coordinates and energies (in hartree) of the calculated $[V_2Cp_2Sb_5]^{2-}$ and $[Nb_2Cp_2Bi_5]^{2-}$ in doublet (D) states at the BP86+D3(BJ)/def2-TZVPP level of theory.

$[(VCp)_2Sb_5]^{2-}$ (D)

Enery = -3477.943483

Sb	2.416521	0.000000	0.000000
Sb	0.687292	-2.313332	0.000000
Sb	0.687292	2.313332	0.000000
V	0.061765	0.000001	1.411057
V	0.061765	0.000001	-1.411057
Sb	-1.980475	1.394488	0.000000
C	-0.898933	0.713203	3.351650
C	-0.898933	-0.713203	3.351650
C	0.457822	1.156021	3.322901
C	1.299150	0.000000	3.304658
C	0.457822	-1.156021	3.322900
C	0.457822	1.156021	-3.322901
C	-0.898933	-0.713203	-3.351650
C	-0.898933	0.713203	-3.351650
C	1.299150	0.000000	-3.304658
C	0.457822	-1.156021	-3.322900
H	-1.777209	1.349055	3.319270
H	0.790281	2.187603	-3.284592
H	-1.777209	1.349055	-3.319270
H	0.790281	2.187603	3.284592
H	2.382722	0.000000	3.262550
H	2.382722	0.000000	-3.262550
Sb	-1.980474	-1.394489	0.000000
H	-1.777209	-1.349055	3.319269
H	0.790281	-2.187603	3.284590
H	0.790281	-2.187603	-3.284590
H	-1.777209	-1.349055	-3.319269

$[(NbCp)_2Bi_5]^{2-}$ (D)

Enery = -1575.911287

Bi	0.000000	0.000000	2.610240
Bi	2.486791	0.000000	0.736096
Bi	-2.486791	0.000000	0.736096
Nb	0.000000	1.474706	0.073112
Nb	0.000000	-1.474706	0.073112
Bi	1.489665	0.000000	-2.120799
Bi	-1.489665	0.000000	-2.120799
C	1.157733	3.543324	0.478362
C	0.000000	3.524248	1.320564
C	0.714125	3.573676	-0.880343
C	-0.714125	3.573676	-0.880343
C	-1.157733	3.543324	0.478362
C	-0.714125	-3.573676	-0.880343
C	-1.157733	-3.543324	0.478362
C	1.157733	-3.543324	0.478362
C	0.000000	-3.524248	1.320564
C	0.714125	-3.573676	-0.880343
H	2.189587	3.505288	0.811121
H	-1.350266	-3.549094	-1.759168
H	0.000000	3.478425	2.404254
H	1.350266	3.549094	-1.759168
H	-1.350266	3.549094	-1.759168
H	-2.189587	-3.505288	0.811121
H	2.189587	-3.505288	0.811121
H	0.000000	-3.478425	2.404254
H	1.350266	-3.549094	-1.759168
H	-2.189587	3.505288	0.811121
