# Supplementary material for: Synthesis of triple-decker sandwich compounds featuring a M–M bond through cyclo-Bi5 and cyclo-Sb5 rings
Source: Nat Chem. 2025 Mar 18;17(4):556–63. doi: 10.1038/s41557-025-01765-4 (PMC11964928; doi:10.1038/s41557-025-01765-4)
Supplement: Supplementary file 4 — Coordinates for compound 1. [file 41557_2025_1765_MOESM4_ESM.pdf]

Sb	2.416521	0.000000	0.000000
Sb	0.687292	-2.313332	0.000000
Sb	0.687292	2.313332	0.000000
V	0.061765	0.000001	1.411057
V	0.061765	0.000001	-1.411057
Sb	-1.980475	1.394488	0.000000
C	-0.898933	0.713203	3.351650
C	-0.898933	-0.713203	3.351650
C	0.457822	1.156021	3.322901
C	1.299150	0.000000	3.304658
C	0.457822	-1.156021	3.322900
C	0.457822	1.156021	-3.322901
C	-0.898933	-0.713203	-3.351650
C	-0.898933	0.713203	-3.351650
C	1.299150	0.000000	-3.304658
C	0.457822	-1.156021	-3.322900
H	-1.777209	1.349055	3.319270
H	0.790281	2.187603	-3.284592
H	-1.777209	1.349055	-3.319270
H	0.790281	2.187603	3.284592
H	2.382722	0.000000	3.262550
H	2.382722	0.000000	-3.262550
Sb	-1.980474	-1.394489	0.000000
H	-1.777209	-1.349055	3.319269
H	0.790281	-2.187603	3.284590
H	0.790281	-2.187603	-3.284590
H	-1.777209	-1.349055	-3.319269
