# Supplementary material for: Synthesis of triple-decker sandwich compounds featuring a M–M bond through cyclo-Bi5 and cyclo-Sb5 rings
Source: Nat Chem. 2025 Mar 18;17(4):556–63. doi: 10.1038/s41557-025-01765-4 (PMC11964928; doi:10.1038/s41557-025-01765-4)
Supplement: Supplementary file 5 — Coordinates for compound 2. [file 41557_2025_1765_MOESM5_ESM.pdf]

Bi	0.000000	0.000000	2.610240
Bi	2.486791	0.000000	0.736096
Bi	-2.486791	0.000000	0.736096
Nb	0.000000	1.474706	0.073112
Nb	0.000000	-1.474706	0.073112
Bi	1.489665	0.000000	-2.120799
Bi	-1.489665	0.000000	-2.120799
C	1.157733	3.543324	0.478362
C	0.000000	3.524248	1.320564
C	0.714125	3.573676	-0.880343
C	-0.714125	3.573676	-0.880343
C	-1.157733	3.543324	0.478362
C	-0.714125	-3.573676	-0.880343
C	-1.157733	-3.543324	0.478362
C	1.157733	-3.543324	0.478362
C	0.000000	-3.524248	1.320564
C	0.714125	-3.573676	-0.880343
H	2.189587	3.505288	0.811121
H	-1.350266	-3.549094	-1.759168
H	0.000000	3.478425	2.404254
H	1.350266	3.549094	-1.759168
H	-1.350266	3.549094	-1.759168
H	-2.189587	-3.505288	0.811121
H	2.189587	-3.505288	0.811121
H	0.000000	-3.478425	2.404254
H	1.350266	-3.549094	-1.759168
H	-2.189587	3.505288	0.811121
